# Design, Synthesis, and Biological Evaluation of Benzimidazole Derivatives as Potential Lassa Virus Inhibitors

**DOI:** 10.3390/molecules28041579

**Published:** 2023-02-07

**Authors:** Jinwei Chen, Likun Xu, Baogang Wang, Dongna Zhang, Liangliang Zhao, Zhuchun Bei, Yabin Song

**Affiliations:** 1 School of Pharmacy, Anhui Medical University, Hefei 230032, China; 2 State Key Laboratory of Pathogen and Biosecurity, Beijing Institute of Microbiology and Epidemiology, Beijing 100071, China

**Keywords:** Lassa virus, glycoprotein complex, benzimidazole derivatives, surface plasmon resonance, pseudovirus

## Abstract

The Lassa virus (LASV) causes Lassa fever, a highly infectious and lethal agent of acute viral hemorrhagic fever. At present, there are still no effective treatments available, creating an urgent need to develop novel therapeutics. Some benzimidazole compounds targeting the arenavirus envelope glycoprotein complex (GPC) are promising inhibitors of LASV. In this study, we synthesized two series of LASV inhibitors based on the benzimidazole structure. Lentiviral pseudotypes bearing the LASV GPC were established to identify virus entry inhibitors. Surface plasmon resonance (SPR) was further used to verify the binding activities of the potential compounds. Compounds **7d−Z**, **7h−Z**, **13c**, **13d**, and **13f** showed relatively excellent antiviral activities with IC_50_ values ranging from 7.58 to 15.46 nM and their SI values above 1251. These five representative compounds exhibited stronger binding affinity with low equilibrium dissociation constants (K_D_ < 8.25 × 10^−7^ M) in SPR study. The compound **7h−Z** displayed the most potent antiviral activity (IC_50_ = 7.58 nM) with a relatively high SI value (2496), which could be further studied as a lead compound. The structure–activity relationship indicated that the compounds with lipophilic and spatially larger substituents might possess higher antiviral activity and a much larger safety margin. This study will provide some good guidance for the development of highly active compounds with a novel skeleton against LASV.

## 1. Introduction

The Lassa virus (LASV), which belongs to the Mammarenavirus of the Arenaviridae family, causes severe viral hemorrhagic fever (HF) in humans and represents a serious public health problem. It has been reported that 58 million people are at risk of contracting LASV, with an estimated 100,000 to 300,000 cases and 5000 deaths per year in West Africa [1]. However, these numbers are likely to be underestimated due to the lack of appropriate diagnosis in poor areas such as rural areas and the non-specific febrile symptoms of Lassa fever [2]. Hospitalized LASV HF patients have a mortality rate of 15–20%, and survivors often suffer permanent damage to their bilateral hearing after surviving the infection [3,4,5,6]. Currently, there are still no licensed vaccines or drugs to prevent and treat LASV infection. According to the Centers for Disease Control and Prevention (CDC), LASV is considered a Category A pathogen due to its high mortality rates and limited therapeutic options [7]. The World Health Organization (WHO) has made the disease the top priority of research and development [8].

As viral invasion is a crucial step in the viral life cycle, the related protein promises one of the most important targets for antiviral drug design. During LASV entry, the glycoprotein complex (GPC) plays a crucial role. GPC is first synthesized as a single polypeptide and then cleaved by signal peptidase and subtilisin/kexin-isoenzyme-1/site-1 proteases (SKI-1/S1P) into three segments: the receptor-binding subunit GP1, the membrane fusion subunit GP2, and the stable signal peptide (SSP) [9,10]. Following the interaction of GP1 with the cellular receptor α-cystine (α-DG) and lysosome-associated membrane protein 1 (LAMP1), viral endocytosis occurs, and GP2 is subjected to a pH-dependent conformational rearrangement, prompting the fusion of viral and endosomal membranes [8,9,11,12,13]. By interacting with GP2 subunits, SSP promotes GPC-mediated membrane fusion at pH and promotes GPC maturation [14,15]. As a category A reagent, LASV requires Biosafety Level 4 (BSL-4) containment, which presents significant obstacles and safety challenges to anti-infective drug discovery for LASV [16]. Therefore, the surrogate assays of LASV GPC incorporation into lentiviral pseudotypes as an antiviral drug screen method is an accepted alternative [17,18,19].

**Figure 1:**
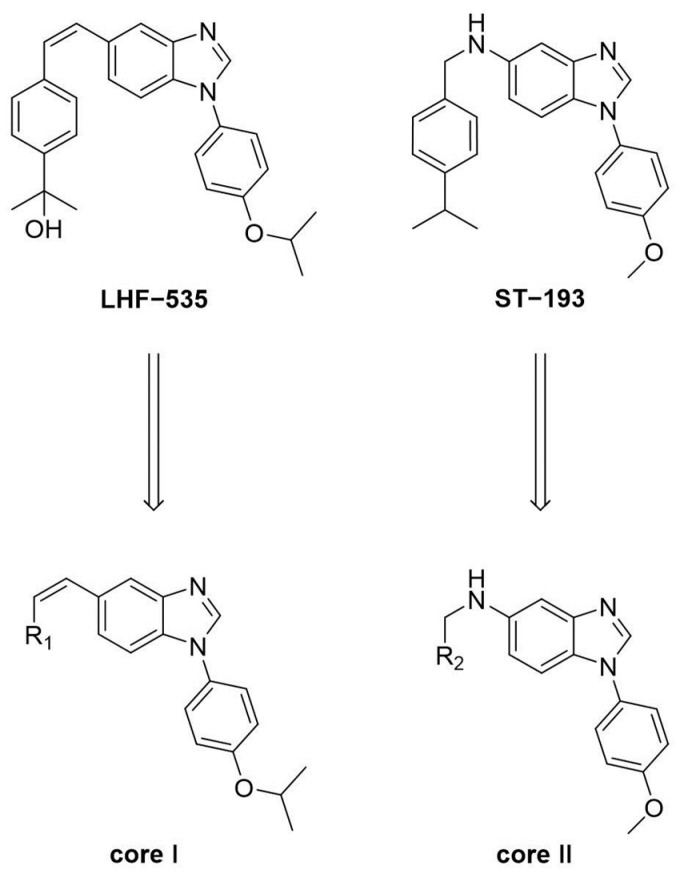
The structure of **LHF−535** and **ST−193** and two kinds of substituted benzimidazole structures.

Currently, the therapeutic strategies for Lassa fever are limited to the off-label use of broad-spectrum antiviral ribavirin, which is usually used in the early stages of the disease [20,21]. As we know, drug repurposing is a promising strategy to identify new uses from approved or experimental drugs, which has also been used in searching for LASV inhibitors [22]. Favipiravir (T−705), an RNA polymerase inhibitor, gave better results than ribavirin in treating LASV infection [23,24]. Losmapimod, a drug used clinically in cardiovascular disease and chronic obstructive pulmonary disease, was identified as an inhibitor of LASV infection [25]. Lacidipine, an anti-hypertension drug, was screened from an FDA-approved drug library as a compound with anti-LASV activity [26]. The antifungal isavuconazole was demonstrated to inhibit the entry of LASV by targeting the stable signal peptide-GP2 subunit interface of LASV GP [27]. However, the antiviral activities of these old drugs are not very satisfactory. Benzimidazole derivatives display a variety of biological activities in many diseases through various mechanisms, such as antiviral [28,29], antimicrobial [30,31], antiproliferative [32,33], and anticancer activities [34,35]. Benzimidazole derivatives are special lead compounds for antiviral drugs, attracting interest from many chemists [18,36,37,38,39]. **LHF−535** and **ST−193**, derivatives of benzimidazoles, are being investigated as promising anti-LASV entry inhibitors (Figure 1) [8,40]. As a small-molecule compound targeting the GPC of LASV, **LHF−535** has broad-spectrum activity against different lineages of LASV and related arenaviruses causing HF disease in South America [39]. **ST−193** with a benzimidazole scaffold also showed potent inhibition of lentiviral pseudotyped viruses expressing an enveloped GPC assay against LASV [18]. Currently, these small molecules are still in the early stage of drug development, and there are no clinically specific therapeutic agents for LASV. Therefore, it is an urgent task to discover new antiviral agents against LASV.

In this study, inspired by these facts, we designed and synthesized two new series of benzimidazole derivatives with high antiviral activities using **LHF−535** and **ST−193** as lead structures (Figure 1). An in vitro infection model of LASV pseudovirus containing firefly luciferase (Fluc) gene and enhanced green fluorescent protein (EGFP) gene was established and used to evaluate the antiviral activities of the target compounds. In addition, we analyzed the binding activities of the target compounds to GP2 protein using the SPR technique and calculated their kinetic parameters. We determined five compounds (**7d−Z**, **7h−Z**, **13c**, **13d**, and **13f**) that inhibit LASV entry, exhibit excellent antiviral activities and low cytotoxicity, as well as better binding affinity to target proteins. **7h−Z** displayed optimal antiviral activity (IC_50_ = 7.58 nM), and SI value is almost twice that of **LHF−535**.

## 2. Results

### 2.1. Chemistry

#### 2.1.1. Synthesis of 5-vinyl-benzo[d]imidazole Derivatives **7a−7h(E/Z)**

**Scheme 1:**
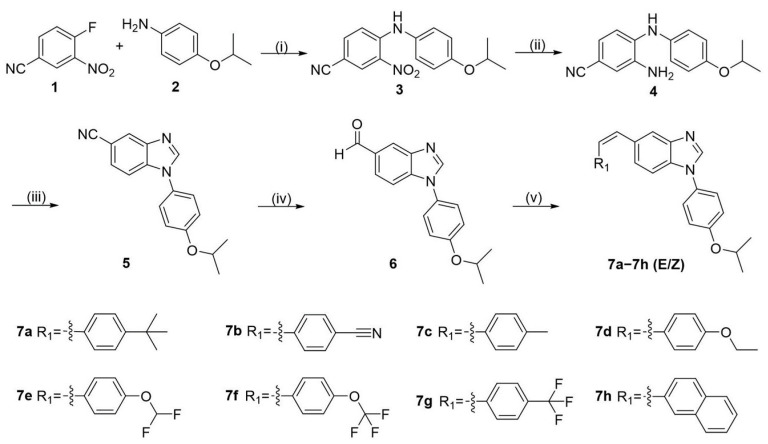
Synthesis of **7a−7h Z/E** (Z: cis structure, E: trans structure). Reagents and conditions: (i) Et_3_N, CH_3_CN, 90 °C; (ii) SnCl_2_·2H_2_O, EtOAc, 50 °C; (iii) Formamidine acetate, EtOH, 88 °C; (iv) Raney Nickel, formic acid, 100 °C; (v) Wittig reagents, *n*-BuLi, THF, −80 °C.

The series I of the target compounds was designed by introducing different substituents R_1_ into the benzimidazole scaffold. The preparation of 5-vinyl-benzo[d]imidazole derivatives **7a−7h(E/Z)** has been accomplished as described in Scheme 1 [41,42]. Initially, compound **3** was synthesized by the substitution reaction using commercially available 4-fluoro-3-nitrobenzonitrile (**1**) and 4-isopropoxyaniline (**2**) as starting materials in the presence of triethylamine. Using tin (II) chloride dihydrate as a reductant, compound **3** was reduced to produce compound **4**. Then, compound **4** was treated with formamidine acetate and subjected to a cycloaddition reaction to give compound **5**. Subsequently, the cyano group was reduced by adding Raney Nickel in the presence of 75% formic acid to obtain compound **6**. Finally, the aldehyde group of compound **6** reacted with suitable Wittig reagents to give the title compounds **7a−7h (Z/E)**. In the reaction, *n*-BuLi was used as a strong base to obtain the Wittig reagents, which then reacted with aldehydes at room temperature to form the corresponding cis-trans isomers. Products with cis structures usually have higher yields.

#### 2.1.2. Synthesis of 5-amino-benzo[d]imidazole Derivatives **13a−13j**

**Scheme 2:**
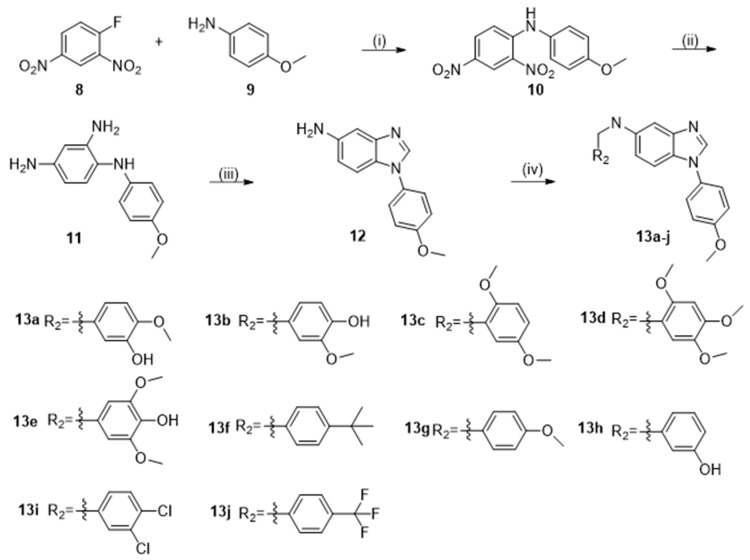
Synthesis of derivatives **13a−13j**. Reagents and conditions: (i) Cesium carbonate, THF, 48 °C; (ii) Sodium hydrosulfite, EtOH, rt; (iii) 4.0 M hydrochloric acid, formic acid, 100 °C; (iv) aromatic aldehydes, DCM, Na(AcO)_3_BH, rt.

The reaction route of compounds **13a−13j** was outlined in Scheme 2 [43]. Firstly, 1-fluoro-2, 4-dinitrobenzene (**8**) was reacted with 4-methoxyaniline (**9**) under the presence of cesium carbonate in THF to obtain compound **10**. Subsequently, compound **10** was reduced to obtain compound **11**, using sodium hydrosulfite as the catalytic reagent for hydrogenation. Compound **11** underwent a cycloaddition reaction in the presence of hydrochloric acid (4.0 M) and formic acid to obtain compound **12**. Lastly, the amino group of compound **12** underwent dehydration condensation and reduction reactions with suitable aromatic aldehydes to give compounds **13a−13j**.

The synthesis methods are simple and feasible and can be used as a general method for synthesizing a series of bioactive benzimidazole derivatives. The structures of these synthesized compounds were further confirmed by NMR. The data were collected in the Supplemental Materials Figures S1–S60.

### 2.2. Biochemical Assays

**Figure 2:**
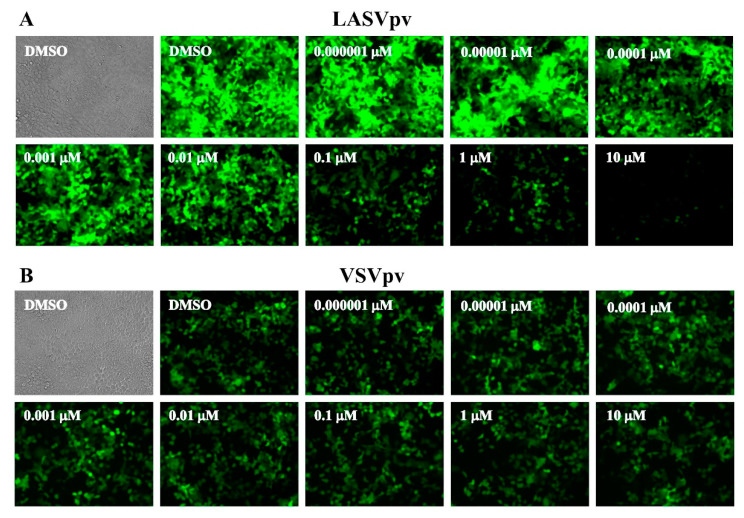
Expression of EGFP in pseudovirus-infected HEK-293 cells. (**A**) The positive drug **LHF−535** inhibited LASVpv infection in a concentration-dependent manner. (**B**) The positive drug **LHF−535** shows little or no effect on pseudotyped viruses expressing the VSVpv.

**Figure 3:**
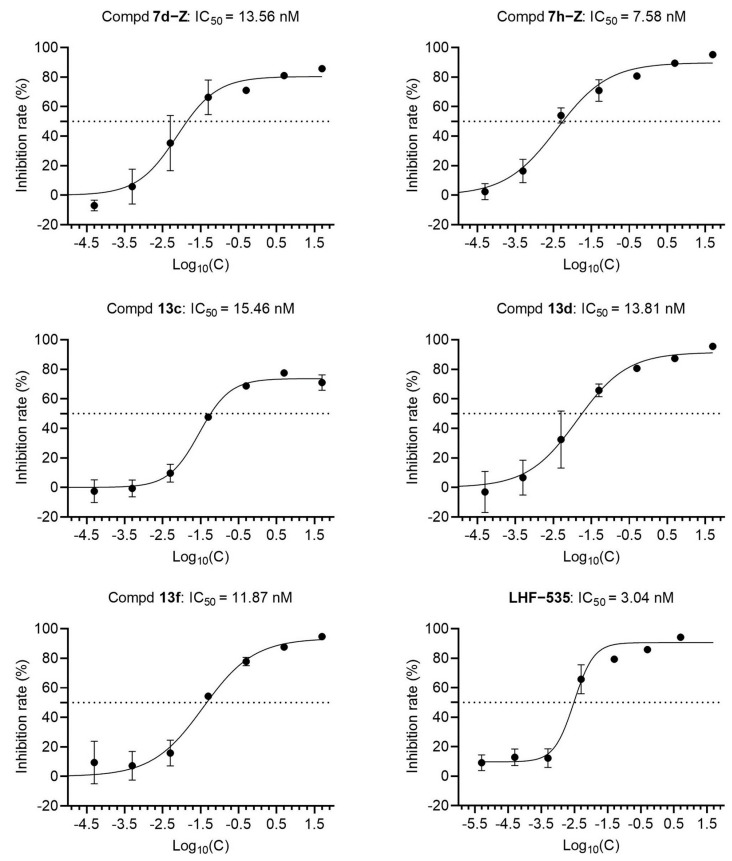
Concentration–response curves of representative compounds **7d−Z**, **7h−Z**, **13c**, **13d**, **13f,** and **LHF−535** to LASVpv.

The LASV is classified as a category A agent, and BSL-4 facilities are required to conduct experiments with live viruses, which restricts the use of live viruses for experiments. In this study, we used HIV-1 pseudotyped viruses bearing the GPC of LASV to establish a validated cell-based pseudovirus infection model (LASVpv) to evaluate the pharmacological activities of the compounds. In this model, VSV pseudotyped lentivirus (VSVpv) constructed with the G protein of the vesicular stomatitis virus was used as a specific control. Based on the pseudoviral model, the antiviral activities of the compounds were determined in HEK-293 cells. **LHF−535** was used as a positive drug to validate the reliability of the model. The results showed that the positive drug did not exhibit an antiviral effect against VSVpv infection in the experimental concentration range (Figure 2B). However, the inhibition of LASVpv infection for the positive drug was concentration-dependent (Figure 2A) with an IC_50_ value of 3.04 nM (Figure 3), which corresponded to the data in the reported literature [39]. Therefore, the model can be used to evaluate the antiviral activities of title compounds. All synthesized compounds exhibit low nanomolar IC_50_ activities against LASVpv, as shown in Table 1 and Table 2. The concentration–response curves of the representative compounds and the positive drug to LASVpv are shown in Figure 3. The IC_50_ values of **7d−Z**, **7h−Z**, **13c**, **13d**, and **13f** were 13.56 nM, 7.58 nM, 15.46 nM, 13.81 nM, and 11.87 nM, respectively, which were closer to the IC_50_ of positive drugs (3.04 nM), and, notably, they had higher SI values.

### 2.3. Initial Inhibitory and Structural Analysis of Benzimidazole Derivatives

Initially, exploring the structure–activity relationships (SAR) of the R_1_ showed that R_1_ with electron-withdrawing groups might reduce the activities of compounds against LASVpv. For example, compounds substituted with 4-(difluoromethoxy)phenyl group (**7e−Z**), 4-(trifluoromethoxy)phenyl (**7f−Z**), and trifluoromethyl phenyl (**7g−Z**) showed a 9 to 11 fold reduction in activities against LASVpv compared to hydroxyisopropyl phenyl. Several other lipophilic groups retained low nanomolar activities of the compounds (**7a−Z**, **7d−Z**, and **7h−Z**, IC_50_ ≤ 15 nM) except the spatially small substituent 4-methylphenyl (**7c−Z**, IC_50_ = 36.89 nM). Among them, compound **7h−Z**, with a more lipophilic naphthyl group, was more active than compounds with other substituents. Compounds with cis configuration showed submicromolar IC_50_ values, while the compounds with trans configuration showed relatively low activities efficiency (IC_50_ > 50.0 nM). This result is consistent with previous studies [41].

For 5-amino-benzo[d]imidazole derivatives **13a−13j**, the replacement of isopropyl (in **ST−193**) with electron-withdrawing groups of dichloromethyl (**13i**) and trifluoromethyl (**13j**) resulted in a decrease in activity. The substitution of R_2_ by the more lipophilic tert-butylphenyl group (**13−f**) increased potency. Compound **13g**, which has a less lipophilic 4-methoxyphenyl group, reduced the activity against LASVpv, except for compound **13c** (IC_50_ = 15.47 nM), which has a spatially larger substituent. However, compound **13d** with large lipophilic 2,4,5-trimethoxyphenyl showed a relative increase in antiviral activity. The introduction of polar hydroxyphenyl (**13a**, **13b**, **13e**, **13h**) led to a significant decrease in activities against LASVpv. Especially, the substitution of 3-hydroxyphenyl (**13h**, IC_50_ > 100 nM) led to the loss of activity.

### 2.4. SPR-Based Binding Assay for Compounds to GP2

Surface plasmon resonance (SPR) is a powerful tool for monitoring interactions between small molecules and target proteins. This interaction was characterized by determining kinetic parameters and affinities [44]. The association rate constant (ka), the dissociation rate constant (k_d_), and the equilibrium dissociation constant (K_D_) can be determined using SPR-based binding assays. Commonly, active compounds bind to ligand proteins with K_D_ values ranging from 10^−7^ to 10^−4^ M [45]. Lower K_D_ values indicate stronger binding.

**Figure 4:**
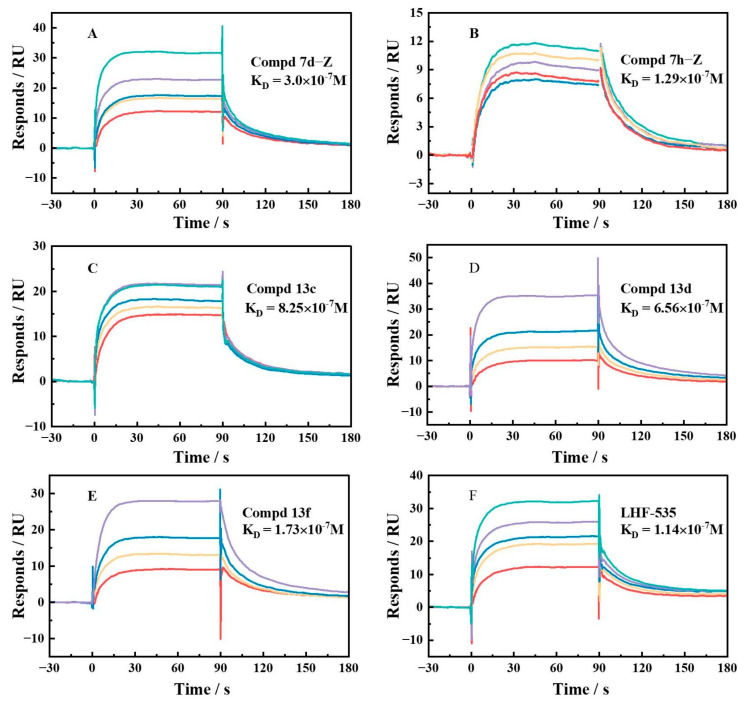
Binding sensory plots of representative compounds (**A**) **7d−Z**, (**B**) **7h−Z**, (**C**) **13c**, (**D**) **13d**, (**E**) **13f**, and (**F**) **LHF−535** interacting with immobilized GP2.

To our knowledge, the evaluation binding affinity of different small compounds towards LASV GP2 using SPR is reported for the first time in our study. Immobilization of GP2 on the SPR sensor surface is achieved by amino coupling, resulting in a stable surface with an immobilization level of approximately 15,783 response units (RU). The binding parameters of title compounds were determined with the chip at concentrations between 0.19 μM and 50.00 μM. Based on Biacore software’s 1:1 binding fitting model, the binding kinetic was analyzed. The K_D_ values of all the tested title compounds binding to GP2 are shown in Table 3. For **7d−Z**, **7h−Z**, **13c**, **13d**, and **13f**, they are nearly within the same quantitative grade compared with **LHF−535** (The positive drug), and for other analytes, their K**_D_** values (>10^–6^ M) are higher. The five representative compounds and the positive drug bound to GP2 with a clear association and dissociation phase (Figure 4). These compounds exhibit distinctive fast-binding and slow-dissociation curves, resulting in a significant and dose-dependent increase in RU. Representative compounds exhibited strong binding activities to GP2 with dissociation constants K_D_ < 8.25 × 10^−7^ M. These results indicated that compounds **7d−Z**, **7h−Z**, **13c**, **13d**, and **13f** had a long duration of drug efficacy and excellent inhibitory potency, which is consistent with the results of LASVpv inhibition assays shown in Table 1 and Table 2.

## 3. Materials and Methods

### 3.1. Chemistry

#### 3.1.1. Chemicals and Instruments

All solvents and chemicals were purchased from commercial suppliers and used without further purification if not indicated. Melting points (Mp) were determined in capillary tubes on a Jiahang melting point JH70L apparatus. The progress of all reactions was routinely monitored by TLC silica gel glass plates (UV wavelength: 254 and 365 nm). The synthesized products were further purified by a Biotage^@^Selekt automated flash purification system. Proton (^1^H) NMR spectra were obtained at 600 MHz in Bruker AVANCE 600 spectrometer using DMSO-*d*6 or CDCl_3_ as the solvent. Carbon (^13^C) NMR spectra were recorded at 151 MHz using the same instrument and solvent conditions. Chemical shifts are marked as parts per million (ppm). High-resolution mass spectroscopy (HRMS) data were analyzed in Agilent 1290II-6460.

#### 3.1.2. Synthesis of 5-vinyl-benzo[d]imidazole Derivatives

*4-((4-isopropoxyphenyl)amino)-3-nitrobenzonitrile(****3****)*. 4-fluoro-3-nitrobenzonitrile **1** (20.00 g, 0.12 mol), 4-isopropoxyaniline **2** (22.76 g, 0.15 mol), and triethylamine (19.46 mL, 0.14 mol) were combined in acetonitrile (100 mL). The mixture was stirred at 90 °C for 24 h. The organic phase was dried with anhydrous sodium sulfate and concentrated under reduced pressure. The crude product was purified by slurrying with tert-butylmethylether and filtered to afford compound **3** (35.08 g, 98%) as a red solid. Mp 130–132 °C. ^1^H NMR (600 MHz, Chloroform-*d*): δ 9.71 (s, 1H), 8.52 (d, *J* = 2.1 Hz, 1H), 7.47 (dd, *J* = 9.1, 2.2 Hz, 1H), 7.16 (d, *J* = 8.9 Hz, 2H), 7.02 (d, *J* = 9.1 Hz, 1H), 6.96 (d, *J* = 8.9 Hz, 2H), 4.61–4.52 (m, 1H), 1.37 (d, *J* = 6.1 Hz, 6H). ^13^C NMR (151 MHz, Chloroform-*d*): δ 157.38, 146.91, 137.26, 132.12, 131.71, 129.01, 127.56, 117.94, 117.16, 116.93, 99.47, 70.43, 22.07. HRMS (ESI) m/z: (M + H)^+^ calcd for C_16_H_15_N_3_O_3_ 298.1113; found 298.1187.

*3-amino-4-((4-isopropoxyphenyl)amino)benzonitrile (**4**)*. A solution of compound **3** (24.00 g, 0.08 mol) in EtOAc (500 mL) was heated to 50 °C, and tin (II) chloride dihydrate (63.75 g 0.28 mol) was added in portions. The mixture was heated for 3 h at 60–62 °C. The reaction mixture was cooled to room temperature, and the pH was adjusted to alkaline by adding aqueous sodium bicarbonate, and this mixture was stirred for 1 h. The reaction mixture was filtered through a pad of Celite. The filtrate was washed with water and EtOAc. The organic phase was dried over anhydrous sodium sulfate overnight and concentrated; the residue product was purified using an automated chromatography system to afford compound **4** as a brownish-red solid (20.50, 95%). Mp 146−147 °C. ^1^H NMR (600 MHz, Chloroform-*d*): δ 7.05 (dd, *J* = 8.3, 2.0 Hz, 1H), 7.02 (s, 1H), 6.97 (d, *J* = 8.9 Hz, 2H), 6.93 (d, *J* = 8.3 Hz, 1H), 6.87 (d, *J* = 8.8 Hz, 2H), 5.53 (s, 1H), 4.55–4.45 (m, 1H), 4.07–3.28 (m, 2H), 1.34 (d, *J* = 6.1 Hz, 6H). ^13^C NMR (151 MHz, Chloroform-*d*): δ 154.50, 139.27, 135.21, 134.03, 125.78, 122.92, 120.11, 120.09, 117.32, 115.24, 102.85, 70.66, 22.24. HRMS (ESI) m/z: (M + H)^+^ calcd for C_16_H_17_N_3_O 268.1372; found 268.1446.

*1-(4-isopropoxyphenyl)-1H-benzo[d]imidazole-5-carbonitrile (**5**).* A mixture of compound **4** (20.00 g, 0.07 mol), formamidine acetate (10.13 g, 0.10 mol), and ethanol (500 mL) was stirred at 88 °C for 9 h. After the mixed solution was cooled to room temperature and stirred overnight, the solvent was removed under reduced pressure; the residue was diluted with water, stirred for 30 min, and filtered. Then, the residue was purified by slurrying with methanol and filtered to afford compound **5** (20.13 g, 97%) as an orange solid. Mp 193−195 °C. ^1^H NMR (600 MHz, Chloroform-*d*): δ 8.22 (s, 1H), 8.21 (d, *J* = 1.7 Hz, 1H), 7.57 (dd, *J* = 8.4, 1.5 Hz, 1H), 7.52 (d, *J* = 8.4 Hz, 1H), 7.39–7.35 (d, *J* = 8.9 Hz, 2H), 7.07 (d, *J* = 8.9 Hz, 2H), 4.67–4.60 (m, 1H), 1.40 (d, *J* = 6.1 Hz, 6H). ^13^C NMR (151 MHz, Chloroform-*d*): δ 158.59, 145.02, 143.02, 137.01, 127.53, 127.13, 126.10, 125.65, 119.71, 117.16, 111.84, 106.37, 70.67, 22.07. HRMS (ESI) m/z: (M + H)^+^ calcd for C_17_H_15_N_3_O 278.1215; found 278.1292.

*1-(4-isopropoxyphenyl)-1H-benzo[d]imidazole-5-carbaldehyde (**6**)*. The mixture of compound **5** (8.40 g, 0.03 mol) and Raney Ni (8.40 g) in 75% formic acid (168 mL) was stirred at 100 °C for 1–2 h. The reaction mixture was filtered through a pad of Celite. The filtrate was washed with water and EtOAc. The organic phase was separated and dried over anhydrous sodium sulfate overnight. It was then filtered and concentrated; the residue was purified using an automated chromatography system to afford compound **6** (8.15 g, 96%) as a white solid. Mp 130–131 °C. ^1^H NMR (600 MHz, Chloroform-*d*): δ 10.09 (s, 1H), 8.34 (d, *J* = 1.5 Hz, 1H), 8.17 (s, 1H), 7.89 (dd, *J* = 8.4, 1.5 Hz, 1H), 7.54 (d, *J* = 8.4 Hz, 1H), 7.38 (d, *J* = 8.9 Hz, 2H), 7.06 (d, *J* = 8.9 Hz, 2H), 4.67–4.58 (m, 1H), 1.39 (d, *J* = 6.1 Hz, 6H). ^13^C NMR (151 MHz, Chloroform-*d*): δ 191.99, 158.39, 144.88, 143.75, 138.58, 132.26, 128.01, 126.03, 124.89, 123.99, 117.11, 111.27, 70.64, 22.07. HRMS (ESI) m/z: (M + H)^+^ calcd for C_17_H_16_N_2_O_2_ 281.1212; found 281.1287.

#### 3.1.3. General Procedure for Synthesis of Compounds **7a−7h(E/Z)**

The corresponding Wittig reagents (1.00 eq.) were added to a stirring solution of tetrahydrofuran (5.00 mL) at −80 °C. Sodium butyllithium (2.50 M in hexanes, 1.05 eq.) was added and stirred for 1 h. Subsequently, compound **6** (1.00 eq.) was added, and the mixed solution was warmed to room temperature slowly. After 12 h, the reaction mixture was concentrated under reduced pressure and purified by flash column chromatography to obtain the products **7a−7h(E/Z)**.

*(Z)-5-(4-(tert-butyl)styryl)-1-(4-isopropoxyphenyl)-1H-benzo[d]imidazole (**7a*****−*Z****)* [41]. Compound **7a−Z** was synthesized according to the general procedure, replacing the corresponding Wittig reagents with (4-(tert-butyl)benzyl)triphenylphosphonium. Compound **7a−Z** was obtained as a light yellow solid in 45% yield. Mp 172–174 °C. ^1^H NMR (600 MHz, Chloroform-*d*): δ 8.06 (s, 1H), 7.84 (s, 1H), 7.42 (d, J = 8.9 Hz, 2H), 7.35 (d, J = 8.4 Hz, 1H), 7.30 (d, J = 8.4 Hz, 1H), 7.28–7.23 (m, 4H), 7.07 (d, J = 8.9 Hz, 2H), 6.74 (d, J = 12.3 Hz, 1H), 6.60 (d, J = 12.2 Hz, 1H), 4.64 (p, J = 6.1 Hz, 1H), 1.43 (d, J = 6.1 Hz, 6H), 1.32 (s, 9H). ^13^C NMR (151 MHz, Chloroform-*d*): δ 157.85, 150.64, 144.46, 143.28, 134.91, 133.96, 132.84, 128.88, 128.50, 127.62, 126.25, 125.73, 122.38, 118.52, 116.99, 110.63, 70.55, 34.74, 31.44, 22.12. HRMS (ESI) m/z: (M + H)^+^ calcd for C_28_H_30_N_2_O 411.2358; found 411.2422.

*(E)-5-(4-(tert-butyl)styryl)-1-(4-isopropoxyphenyl)-1H-benzo[d]imidazole (**7a*****−*E****)* [41]. Compound **7a−E** was synthesized according to the general procedure, replacing the corresponding Wittig reagents with (4-(tert-butyl)benzyl)triphenylphosphonium. Compound **7a−E** was obtained as a white solid in a yield of 23%. Mp 161–162 °C. ^1^H NMR (600 MHz, Chloroform-*d*): δ 8.06 (s, 1H), 7.97 (s, 1H), 7.47 (ddd, *J* = 59.1, 21.1, 8.9 Hz, 8H), 7.25 (d, *J* = 16.1 Hz, 1H), 7.14 (d, *J* = 16.2 Hz, 1H), 7.06 (d, *J* = 8.5 Hz, 2H), 4.63 (p, *J* = 6.5 Hz, 1H), 1.41 (d, *J* = 6.3 Hz, 6H), 1.35 (s, 9H). ^13^C NMR (151 MHz, Chloroform-*d*): δ 157.79, 150.14, 144.06, 143.01, 134.46, 133.43, 132.46, 129.92, 129.50, 128.94, 128.72, 125.70, 125.26, 125.04, 120.69, 116.96, 110.14, 70.54, 34.67, 31.42, 22.12. HRMS (ESI) m/z: (M + H)^+^ calcd for C_28_H_30_N_2_O 411.2358; found 411.2420.

*(Z)-4-(2-(1-(4-isopropoxyphenyl)-1H-benzo[d]imidazol-5-yl) vinyl) benzonitrile (**7b*****−*Z****)* [41]. Compound **7b−Z** was synthesized according to the general procedure, replacing the corresponding Wittig reagents with (4-cyanobenzyl) triphenylphosphonium. Compound **7b−Z** was obtained as a yellow solid in a yield of 36%. Mp 165–166 °C. ^1^H NMR (600 MHz, Chloroform-*d*): δ 8.03 (s, 1H), 7.71 (s, 1H), 7.47 (d, *J* = 8.4 Hz, 2H), 7.37 (d, *J* = 8.8 Hz, 2H), 7.34 (d, *J* = 8.2 Hz, 2H), 7.31 (d, *J* = 8.4 Hz, 1H), 7.12 (dd, *J* = 8.4, 1.7 Hz, 1H), 7.04 (d, *J* = 8.9 Hz, 2H), 6.91 (d, *J* = 12.2 Hz, 1H), 6.58 (d, *J* = 12.1 Hz, 1H), 4.65–4.57 (m, 1H), 1.39 (d, *J* = 6.1 Hz, 6H). ^13^C NMR (151 MHz, Chloroform-*d*): δ 157.90, 144.12, 143.37, 142.47, 133.91, 133.76, 132.16, 130.97, 129.69, 128.62, 127.75, 125.69, 124.76, 120.92, 119.11, 116.97, 110.50, 110.40, 70.53, 22.07. HRMS (ESI) m/z: (M + H)^+^ calcd for C_25_H_21_N_3_O 380.1685; found 380.1747.

*(E)-4-(2-(1-(4-isopropoxyphenyl)-1H-benzo[d]imidazol-5-yl) vinyl) benzonitrile (**7b*****−*E****)* [41]. Compound **7b−E** was synthesized according to the general procedure, replacing the corresponding Wittig reagents with (4-cyanobenzyl) triphenylphosphonium. Compound **7b−E** was obtained as a yellow solid in a yield of 20%. Mp 161–162 °C. ^1^H NMR (600 MHz, Chloroform-*d*): δ 8.07 (s, 1H), 7.99 (s, 1H), 7.63 (d, *J* = 8.4 Hz, 2H), 7.60 (d, *J* = 8.4 Hz, 2H), 7.53 (d, *J* = 8.4 Hz, 1H), 7.45 (d, *J* = 8.4 Hz, 1H), 7.41–7.34 (m, 3H), 7.11 (d, *J* = 16.2 Hz, 1H), 7.05 (d, *J* = 8.9 Hz, 2H), 4.68–4.59 (m, 1H), 1.40 (d, *J* = 6.1 Hz, 6H). ^13^C NMR (151 MHz, Chloroform-*d*): δ 157.97, 144.40, 143.66, 142.21, 134.70, 133.03, 132.59, 131.49, 128.59, 126.83, 125.74, 125.72, 122.67, 119.33, 119.26, 117.00, 110.91, 110.31, 70.55, 22.09. HRMS (ESI) m/z: (M + H)^+^ calcd for C_25_H_21_N_3_O 380.1685; found 380.1747.

*(Z)-1-(4-isopropoxyphenyl)-5-(4-methylstyryl)-1H-benzo[d]imidazole (**7c*****−*Z****)*. Compound **7c−Z** was synthesized according to the general procedure, replacing the corresponding Wittig reagents with (4-methylbenzyl)triphenylphosphonium. Compound **7c−Z** was obtained as a yellow solid in a yield of 25%. Mp 168–169 °C. ^1^H NMR (600 MHz, Chloroform-*d*): δ 8.01 (s, 1H), 7.77 (s, 1H), 7.37 (d, *J* = 8.5 Hz, 2H), 7.29 (d, *J* = 8.5 Hz, 1H), 7.23 (d, *J* = 8.5 Hz, 1H), 7.18 (d, *J* = 7.9 Hz, 2H), 7.07–6.94 (m, 4H), 6.71 (d, *J* = 12.2 Hz, 1H), 6.58 (d, *J* = 12.2 Hz, 1H), 4.66–4.57 (m, 1H), 2.30 (s, 3H), 1.39 (d, *J* = 6.2 Hz, 6H). ^13^C NMR (151 MHz, Chloroform-*d*): δ 157.75, 144.06, 142.97, 136.83, 134.54, 133.39, 132.30, 129.95, 129.59, 129.06, 128.90, 125.64, 125.04, 120.77, 116.93, 110.06, 70.51, 22.09, 21.34. HRMS (ESI) m/z: (M + H)^+^ calcd for C_25_H_24_N_2_O 369.1889; found 369.1955.

*(E)-1-(4-isopropoxyphenyl)-5-(4-methylstyryl)-1H-benzo[d]imidazole (**7c*****−*E****)*. Compound **7c−E** was synthesized according to the general procedure, replacing the corresponding Wittig reagents with (4-methylbenzyl)triphenylphosphonium. Compound **7c−E** was obtained as a yellow solid in a yield of 15%. Mp 168–170 °C. ^1^H NMR (600 MHz, Chloroform-*d*): δ 8.04 (s, 1H), 7.96 (s, 1H), 7.52 (d, *J* = 8.7 Hz, 1H), 7.49–7.36 (m, 5H), 7.23 (d, *J* = 16.3 Hz, 1H), 7.18 (d, *J* = 7.9 Hz, 2H), 7.12 (d, *J* = 16.3 Hz, 1H), 7.05 (d, *J* = 8.8 Hz, 2H), 4.67–4.57 (m, 1H), 2.37 (s, 3H), 1.40 (d, *J* = 6.1 Hz, 6H). ^13^C NMR (151 MHz, Chloroform-*d*): δ 157.84, 144.47, 143.26, 137.35, 134.88, 133.95, 132.80, 129.51, 128.87, 128.25, 127.72, 126.43, 125.70, 122.35, 118.50, 116.98, 110.63, 70.54, 22.11, 21.38. HRMS (ESI) m/z: (M + H)^+^ calcd for C_25_H_24_N_2_O 369.1889; found 369.1953.

*(Z)-5-(4-ethoxystyryl)-1-(4-isopropoxyphenyl)-1H-benzo[d]imidazole (**7d*****−*Z****)*. Compound **7d−Z** was synthesized according to the general procedure, replacing the corresponding Wittig reagents with 4-ethoxybenzyl)triphenylphosphonium. Compound **7d−Z** was obtained as a yellow solid in a yield of 50%. Mp 161–162 °C. ^1^H NMR (600 MHz, Chloroform-*d*): δ 8.05 (s, 1H), 7.82 (s, 1H), 7.41 (d, *J* = 8.9 Hz, 2H), 7.33 (d, *J* = 8.4 Hz, 1H), 7.27 (d, *J* = 8.5 Hz, 1H), 7.24 (d, *J* = 6.9 Hz, 2H), 7.06 (d, *J* = 6.9 Hz, 2H), 6.77 (d, *J* = 6.7 Hz, 2H), 6.69 (d, *J* = 12.1 Hz, 1H), 6.58 (d, *J* = 12.2 Hz, 1H), 4.64 (dtq, *J* = 9.2, 5.8, 3.2, 2.6 Hz, 1H), 4.02 (dt, *J* = 7.3, 2.4 Hz, 2H), 1.42 (dt, *J* = 5.0, 2.5 Hz, 9H). ^13^C NMR (151 MHz, Chloroform-*d*): δ 158.08, 157.72, 144.06, 142.92, 133.30, 132.45, 130.21, 129.75, 129.24, 129.04, 128.88, 125.60, 124.99, 120.66, 116.91, 114.25, 110.07, 70.48, 63.38, 22.07, 14.94. HRMS (ESI) m/z: (M + H)^+^ calcd for C_26_H_26_N_2_O_2_, 399.1994; found 399.2057.

*(E)-5-(4-ethoxystyryl)-1-(4-isopropoxyphenyl)-1H-benzo[d]imidazole(**7d*****−*E****)*. Compound **7d−E** was synthesized according to the general procedure, replacing the corresponding Wittig reagents with 4-ethoxybenzyl)triphenylphosphonium. Compound **7d−E** was obtained as a yellow solid in a yield of 30%. Mp 152–153 °C. ^1^H NMR (600 MHz, Chloroform-*d*): δ 8.04 (s, 1H), 7.94 (s, 1H), 7.59–7.36 (m, 6H), 7.18–7.00 (m, 4H), 6.89 (d, *J* = 6.2 Hz, 2H), 4.67–4.56 (m, 1H), 4.10–4.01 (m, 2H), 1.51–1.35 (m, 9H). ^13^C NMR (151 MHz, Chloroform-*d*): δ 158.62, 157.81, 144.44, 143.20, 133.79, 132.98, 130.33, 128.88, 127.69, 127.38, 127.05, 125.68, 122.26, 118.24, 116.97, 114.79, 110.60, 70.53, 63.60, 22.10, 14.98. HRMS (ESI) m/z: (M + H)^+^ calcd for C_26_H_26_N_2_O_2_ 399.1994; found 399.2060.

*(Z)-5-(4-(difluoromethoxy)styryl)-1-(4-isopropoxyphenyl)-1H-benzo[d]imidazole (**7e*****−*Z****)*. Compound **7e−Z** was synthesized according to the general procedure, replacing the corresponding Wittig reagents with (4-(difluoromethoxy)benzyl)triphenylphosphonium. Compound **7e−Z** was obtained as a yellow solid in a yield of 30%. Mp 173–174 °C. ^1^H NMR (600 MHz, Chloroform-*d*): δ 8.02 (s, 1H), 7.74 (d, *J* = 1.6 Hz, 1H), 7.37 (d, *J* = 8.9 Hz, 2H), 7.31 (d, *J* = 8.4 Hz, 1H), 7.26 (d, *J* = 8.7 Hz, 2H), 7.18 (dd, *J* = 8.4, 1.7 Hz, 1H), 7.03 (d, *J* = 8.9 Hz, 2H), 6.94 (d, *J* = 8.6 Hz, 2H), 6.76 (d, *J* = 12.1 Hz, 1H), 6.56 (d, *J* = 12.1 Hz, 1H), 6.48 (t, *J* = 74.1 Hz, 1H), 4.65–4.56 (m, 1H), 1.39 (d, *J* = 6.1 Hz, 6H). ^13^C NMR (151 MHz, Chloroform-*d*): δ 157.83, 150.20 (t, *J* = 2.7 Hz), 144.09, 143.14, 134.75, 133.57, 131.75, 131.06, 130.50, 128.79, 128.31, 125.67, 124.90, 120.77, 119.20, 116.96, 116.08 (t, *J* = 259.0 Hz), 110.29, 70.53, 22.08. HRMS (ESI) m/z: (M + H)^+^ calcd for C_25_H_22_F_2_N_2_O_2_ 421.1649; found 421.1712.

*(E)-5-(4-(difluoromethoxy)styryl)-1-(4-isopropoxyphenyl)-1H-benzo[d]imidazole (**7e*****−*E****)*. Compound **7e−E** was synthesized according to the general procedure, replacing the corresponding Wittig reagents with (4-(difluoromethoxy)benzyl)triphenylphosphonium. Compound **7e−E** was obtained as a yellow solid in a yield of 26%. Mp 173–174 °C. ^1^H NMR (600 MHz, Chloroform-*d*): δ 8.05 (s, 1H), 7.96 (s, 1H), 7.56–7.48 (m, 3H), 7.43 (d, *J* = 8.4 Hz, 1H), 7.38 (d, *J* = 8.8 Hz, 2H), 7.21 (d, *J* = 16.3 Hz, 1H), 7.14–7.07 (m, 3H), 7.05 (d, *J* = 8.8 Hz, 2H), 6.52 (t, *J* = 74.0 Hz, 1H), 4.67–4.58 (m, 1H), 1.40 (d, *J* = 6.1 Hz, 6H). ^13^C NMR (151 MHz, Chloroform-*d*): δ 157.87, 150.46 (t, *J* = 2.7 Hz), 144.48, 143.40, 135.12, 134.17, 132.29, 129.59, 128.78, 127.76, 126.37, 125.70, 122.39, 119.84, 118.70, 116.98, 116.08 (t, *J* = 260.0 Hz), 110.72, 70.54, 22.10. HRMS (ESI) m/z: (M + H)^+^ calcd for C_25_H_22_F_2_N_2_O_2_ 421.1649; found 421.1713.

*(Z)-1-(4-isopropoxyphenyl)-5-(4-(trifluoromethoxy)styryl)-1H-benzo[d]imidazole (**7f*****−*Z****)*. Compound **7f−Z** was synthesized according to the general procedure, replacing the corresponding Wittig reagents with triphenyl(4-(trifluoromethoxy)benzyl)phosphonium. Compound **7f−Z** was obtained as a yellow solid in a yield of 30%. Mp 161–162 °C. ^1^H NMR (600 MHz, Chloroform-*d*): δ 8.03 (s, 1H), 7.75 (d, *J* = 1.7 Hz, 1H), 7.37 (d, *J* = 8.9 Hz, 2H), 7.31 (d, *J* = 8.4 Hz, 1H), 7.28 (d, *J* = 8.5 Hz, 2H), 7.18 (dd, *J* = 8.5, 1.7 Hz, 1H), 7.07–7.00 (m, 4H), 6.80 (d, *J* = 12.2 Hz, 1H), 6.56 (d, *J* = 12.1 Hz, 1H), 4.65–4.56 (m, 1H), 1.39 (d, *J* = 6.1 Hz, 6H). ^13^C NMR (151 MHz, Chloroform-*d*): δ 157.87, 148.10 (q, *J* = 1.5 Hz), 144.12, 143.20, 136.18, 133.66, 131.62, 131.57, 130.42, 128.79, 128.08, 125.70, 124.84, 120.85, 120.76, 120.57 (q, *J* = 257.2 Hz), 116.98, 110.35, 70.55, 22.10. HRMS (ESI) m/z: (M + H)^+^ calcd for C_25_H_21_F_3_N_2_O_2_ 439.1555; found 439.1619.

*(E)-1-(4-isopropoxyphenyl)-5-(4-(trifluoromethoxy)styryl)-1H-benzo[d]imidazole (**7f*****−*E****)*. Compound **7f−E** was synthesized according to the general procedure, replacing the corresponding Wittig reagents with triphenyl(4-(trifluoromethoxy)benzyl)phosphonium. Compound **7f−E** was obtained as a yellow solid in a yield of 10%. Mp 160–162 °C. ^1^H NMR (600 MHz, Chloroform-*d*): δ 8.05 (s, 1H), 7.97 (d, *J* = 1.7 Hz, 1H), 7.55 (d, *J* = 8.8 Hz, 2H), 7.52 (dd, *J* = 8.5, 1.7 Hz, 1H), 7.44 (d, *J* = 8.4 Hz, 1H), 7.39 (d, *J* = 8.9 Hz, 2H), 7.25–7.19 (m, 3H), 7.11 (d, *J* = 16.2 Hz, 1H), 7.05 (d, *J* = 8.8 Hz, 2H), 4.66–4.57 (m, 1H), 1.40 (d, *J* = 6.1 Hz, 6H). ^13^C NMR (151 MHz, Chloroform-*d*): δ 157.93, 148.41, 144.44, 143.45, 136.49, 134.28, 132.17, 130.30, 128.76, 127.65, 126.17, 125.74, 122.47, 121.33, 120.63 (q, *J* = 257.2 Hz), 118.81, 117.01, 110.77, 70.56, 22.10. HRMS (ESI) m/z: (M + H)^+^ calcd for C_25_H_21_F_3_N_2_O_2_ 439.1555; found 439.1619.

*(Z)-1-(4-isopropoxyphenyl)-5-(4-(trifluoromethyl)styryl)-1H-benzo[d]imidazole (**7g*****−*Z****)*. Compound **7g−Z** was synthesized according to the general procedure, replacing the corresponding Wittig reagents with triphenyl(4-(trifluoromethyl)benzyl)phosphonium. Compound **7g−Z** was obtained as a yellow solid in a yield of 35%. Mp 165–166 °C. ^1^H NMR (600 MHz, Chloroform-*d*): δ 8.03 (s, 1H), 7.74 (s, 1H), 7.45 (d, *J* = 8.3 Hz, 2H), 7.39–7.33 (m, 4H), 7.30 (dd, *J* = 8.5 Hz, 1H), 7.16 (d, *J* = 8.4 Hz, 1H), 7.06–7.00 (m, 2H), 6.86 (d, *J* = 12.2 Hz, 1H), 6.60 (d, *J* = 12.2 Hz, 1H), 4.66–4.55 (m, 1H), 1.41–1.35 (m, 6H). ^13^C NMR (151 MHz, Chloroform-*d*): δ 157.87, 144.10, 143.25, 141.25, 133.76, 132.72, 131.33, 129.27, 128.90 (q, *J* = 32.0 Hz), 128.72, 128.09, 125.68, 125.29 (q, *J* = 3.9 Hz), 124.85, 124.29 (q, *J* = 272.3 Hz), 120.89, 116.97, 110.39, 70.53, 22.07. HRMS (ESI) m/z: (M + H)^+^ calcd for C_25_H_21_F_3_N_2_O 423.1606; found 423.1667.

*(E)-1-(4-isopropoxyphenyl)-5-(4-(trifluoromethyl)styryl)-1H-benzo[d]imidazole (**7g*****−*E****)*. Compound **7g−E** was synthesized according to the general procedure, replacing the corresponding Wittig reagents with tri-phenyl(4-(trifluoromethyl)benzyl)phosphonium. Compound **7g−E** was obtained as a yellow solid in a yield of 20%. Mp 166–167 °C. ^1^H NMR (600 MHz, Chloroform-*d*): δ 8.06 (s, 1H), 7.99 (s, 1H), 7.69–7.57 (m, 4H), 7.53 (d, *J* = 8.5 Hz, 1H), 7.45 (d, *J* = 5.2 Hz, 1H), 7.39 (d, *J* = 5.2 Hz, 2H), 7.35 (d, *J* = 16.1 Hz, 1H), 7.15 (d, *J* = 16.2 Hz, 1H), 7.06 (d, *J* = 5.4 Hz, 2H), 4.66–4.57 (m, 1H), 1.44–1.35 (m, 6H). ^13^C NMR (151 MHz, Chloroform-*d*): δ 157.97, 144.44, 143.55, 141.18, 134.50, 131.89, 131.81, 129.08 (q, *J* = 32.0 Hz), 128.71, 126.57, 126.18, 125.76, 125.73, 124.41 (q, *J* = 271.5 Hz), 122.61, 119.10, 117.02, 110.84, 70.58, 22.11. HRMS (ESI) m/z: (M + H)^+^ calcd for C_25_H_21_F_3_N_2_O 423.1606; found 423.1668.

*(Z)-1-(4-isopropoxyphenyl)-5-(2-(naphthalene-2-yl)vinyl)-1H-benzo[d]imidazole (**7h*****−*Z****)*. Compound **7h−Z** was synthesized according to the general procedure, replacing the corresponding Wittig reagents with (naphthalen-2-ylmethyl)triphenylphosphonium. Compound **7h−Z** was obtained as a brown solid in a yield of 26%. Mp 170–171 °C. ^1^H NMR (600 MHz, Chloroform-*d*): δ 8.02 (s, 1H), 7.80 (s, 1H), 7.77 (s, 1H), 7.74–7.69 (m, 1H), 7.73–7.69 (m, 1H), 7.62 (d, *J* = 8.5 Hz, 1H), 7.43–7.40 (m, 2H), 7.39–7.35 (m, 3H), 7.28–7.22 (m, 2H), 7.02 (d, *J* = 8.9 Hz, 2H), 6.84 (d, *J* = 12.1 Hz, 1H), 6.78 (d, *J* = 12.2 Hz, 1H), 4.65–4.56 (m, 1H), 1.38 (d, *J* = 6.1 Hz, 6H). ^13^C NMR (151 MHz, Chloroform-*d*): δ 157.79, 143.99, 143.07, 135.23, 133.65, 133.53, 132.67, 132.16, 131.02, 129.56, 128.83, 128.11, 128.02, 127.73, 127.65, 127.10, 126.06, 125.91, 125.65, 125.12, 120.99, 116.95, 110.13, 70.52, 22.09. HRMS (ESI) m/z: (M + H)^+^ calcd for C_28_H_24_N_2_O 405.1889; found 405.1955.

*(E)-1-(4-isopropoxyphenyl)-5-(2-(naphthalen-2-yl)vinyl)-1H-benzo[d]imidazole (**7h*****−*E****)*. Compound **7h−Z** was synthesized according to the general procedure, replacing the corresponding Wittig reagents with (naphthalen-2-ylmethyl)triphenylphosphonium. Compound **7h−E** was obtained as a yellow solid in a yield of 15%. Mp 172–173 °C. ^1^H NMR (600 MHz, Chloroform-*d*): δ 8.06 (s, 1H), 8.03 (s, 1H), 7.88 (s, 1H), 7.85–7.80 (m, 3H), 7.79 (d, *J* = 8.5 Hz, 1H), 7.58 (d, *J* = 8.4 Hz, 1H), 7.48–7.38 (m, 6H), 7.31 (d, *J* = 16.2 Hz, 1H), 7.06 (d, *J* = 8.8 Hz, 2H), 4.68–4.59 (m, 1H), 1.41 (d, *J* = 6.1 Hz, 6H). ^13^C NMR (151 MHz, Chloroform-*d*): δ 157.86, 144.46, 143.34, 135.17, 134.12, 133.88, 133.05, 132.63, 129.60, 128.81, 128.40, 128.08, 127.84, 127.81, 126.49, 126.41, 125.88, 125.72, 123.65, 122.48, 118.69, 116.98, 110.73, 70.54, 22.11. HRMS (ESI) m/z: (M + H)^+^ calcd for C_28_H_24_N_2_O 405.1889; found 405.1951.

#### 3.1.4. General Procedure for Synthesis of Compounds **13a−13j**

To a mixture of compound **12** (1.00 eq.) and relatively aromatic aldehyde (R_2_-CHO, 1.00 eq.) in 1, 2-dichloroethane (3 mL) was added the Na(AcO)_3_BH (1.50 eq.) and stirred at room temperature for 6 h. After the mixture was stirred for 6 h, TLC monitors showed the complete consumption of compound **12**. Following cooling, the mixture was divided between EtOAc and H_2_O, and the layers were separated, with the aqueous layer extracted with EtOAc and the organic layer washed with H_2_O. The organic phase was dried with anhydrous sodium sulfate and concentrated under reduced pressure. The residue was purified by flash column chromatography to obtain the products **13a−13j**.

*2-methoxy-5-(((1-(4-methoxyphenyl)-1H-benzo[d]imidazol-5-yl)amino)methyl)phenol (**13a**)*. Compound **13a** was synthesized according to the general procedure, replacing the corresponding aromatic aldehyde with isovanillin. Compound **13a** was obtained as a white solid in a yield of 87%. Mp 182.1–183.3 °C. ^1^H NMR (600 MHz, Chloroform-*d*): δ 7.892 (s, 1H), 7.372 (t, 1H, *J*1 = 4.8, *J*2 = 1.8), 7.357 (t, 1H, *J*1 = 2.4, *J*2 = 3.0), 7.228 (d, 1H, *J* = 10.4) 7.034 (s, 2H), 7.019 (t, 1H, *J*1 = 2.4, *J*2 = 3.6), 6.978 (d, 1H, *J* = 1.8), 6.876 (m, 1H), 6.805 (dd, 1H, *J* = 10.4), 6.689 (dd, 1H) 5.666 (s, 1H), 4.274 (s, 2H), 3.852 (m, 6H). ^13^C NMR (151 MHz, DMSO-*d*6): δ 158.72, 146.89, 146.82, 145.65, 145.44, 142.60, 133.41, 129.78, 126.11, 125.15, 118.27, 115.52, 115.02, 112.92, 112.61, 110.91, 100.66, 56.13, 55.95, 47.28, 40.35, 40.21, 40.07, 39.94, 39.80, 39.66, 39.52. HRMS (ESI) m/z: (M + H)^+^ calcd for C_22_H_21_N_3_O_3_ 376.1656; found 376.1654.

*2-methoxy-4-(((1-(4-methoxyphenyl)-1H-benzo[d]imidazol-5-yl)amino)methyl)phenol (**13b**)*. Compound **13b** was synthesized according to the general procedure, replacing the corresponding aromatic aldehyde with vanillin. Compound **13b** was obtained as a yellow solid in a yield of 91%. Mp 157.6–159.0 °C. ^1^H NMR (600 MHz, Chloroform-*d*): δ7.908 (s, 1H), 7.374 (t, 1H, *J*1 = *J*2 = 2.4), 7.367 (t, 1H, *J*1 = 2.4, *J*2 = 3.6), 7.227 (d, 1H, *J* = 7.8), 7.066 (d, 1H), 7. 039 (t, 1H, *J* = 2.4), 7. 027 (t, 1H, *J* = 1.8), 6.932 (d, 1H, *J* = 1.8), 6.891 (dd, 1H, *J* = 8.4), 6.874 (d, 1H, *J* = 7.8), 6.703 (dd, 1H, *J* = 6.6), 5.598 (s, 1H), 4.283 (s, 2H), 3.868 (d, 6H, *J* = 3.0). ^13^C NMR (151 MHz, DMSO-*d*6): δ 158.71, 147.98, 145.74, 145.66, 145.44, 142.61, 131.50, 129.79, 126.16, 125.15, 120.12, 115.63, 115.53, 112.99, 112.05, 110.88, 100.78, 56.01, 55.96, 47.74. HRMS (ESI) m/z: (M + H)^+^ calcd for C_22_H_21_N_3_O_3_ 376.1656; found 376.1652.

*N-(2, 4-dimethoxybenzyl)-1-(4-methoxyphenyl)-1H-benzo[d]imidazol-5-amine (**13c**)*. Compound **13c** was synthesized according to the general procedure, replacing the corresponding aromatic aldehyde with 2, 4-dimethoxybenzaldehyde. Compound **13c** was obtained as a yellow solid in a yield of 88%. Mp 143.1–145.2 °C. ^1^H NMR (600 MHz, Chloroform-*d*): δ 8.517 (s, 1H), 7.385–7.412 (m, 3H), 7.112 (s, 1H), 7.097 (s, 1H), 7.063 (s, 1H), 6.924 (d, 1H, *J* = 7.8), 6.845 (s, 1H), 6.829 (s, 1H), 6.815 (s, 1H), 6.776 (d, 1H, *J* = 3.0), 4.377 (s, 2H), 3.908 (s, 3H), 3.897 (s, 3H), 3.733 (s, 3H). ^13^C NMR (151 MHz, DMSO-*d*6): δ 160.58, 141.36, 139.40, 131.50, 131.06, 129.73, 129.53, 127.99, 126.88, 126.87, 115.86, 115.68, 113.83, 56.48, 56.19, 45.82. HRMS (ESI) m/z: (M + H)^+^ calcd for C_23_H_23_N_3_O_3_ 390.1812; found 390.1810.

*1-(4-methoxyphenyl)-N-(2, 4,5-trimethoxybenzyl)-1H-benzo[d]imidazol-5-amine (**13d**)*. Compound **13d** was synthesized according to the general procedure, replacing the corresponding aromatic aldehyde with 2, 4,5-trimethoxybenzaldehyde. Compound **13d** was obtained as an orange solid in a yield of 88%. Mp 167.8–169.8 °C. ^1^H NMR (600 MHz, Chloroform-*d*): δ 7.894 (s, 1H), 7.374 (t, 1H, *J*1 = 3.6, *J*2 = 1.8), 7.360 (t, 1H, *J*1 = 1.8, *J*2 = 3.6), 7.226 (d, 1H, *J* = 8.4), 7.094 (d, 1H, *J* = 1.8), 7. 033 (t, 1H, *J*1 = 3.6, *J*2 = 1.8), 7. 019 (t, 1H, *J*1 = 1.8, *J*2 = 3.6), 6.920 (s, 1H), 6.716 (dd, 1H, *J* = 6.6), 6.536 (s, 1H), 4.304 (s, 2H), 3.870 (s, 3H), 3.860 (s, 3H), 3.839 (s, 3H), 3.779 (s, 3H). ^13^C NMR (151 MHz, DMSO-*d*6): δ 203.62, 158.72, 156.07, 151.76, 148.86, 145.73, 145.46, 142.98, 142.64, 125.15, 119.33, 115.53, 114.17, 112.89, 110.93, 100.64, 98.73, 56.92, 56.68, 56.34, 55.96, 42.08. HRMS (ESI) m/z: (M + H)^+^ calcd for C_24_H_25_N_3_O_4_ 420.1918; found 420.1920.

*2, 6-dimethoxy-4-(((1-(4-methoxyphenyl)-1H-benzo[d]imidazol-5-yl)amino)methyl)phenol (**13e**)*. Compound **13e** was synthesized according to the general procedure, replacing the corresponding aromatic aldehyde with Syringaldehyde. Compound **13e** was obtained as an orange solid in a yield of 95%. Mp 115.7–118.1 °C. ^1^H NMR (600 MHz, Chloroform-*d*): δ 7.917 (d, 1H, *J* = 3.6), 7.377 (t, 1H, *J*1 = *J*2 = 1.8), 7.366 (t, 1H, *J*1 = 1.8, *J*2 = 3.6), 7.244 (d, 1H, *J* = 6.0), 7.068 (d, 1H, *J* = 2.4), 7. 037 (t, 1H, *J*1 = 1.8, *J*2 = 1.2), 7. 025 (t, 1H, *J*1 = 2.4, *J*2 = 3.0), 6.708 (dd, 1H, *J* = 6.0), 6.651 (s, 2H), 6.482 (s, 1H),5.407 (d, 1H), 4.528 (s, 1H), 4.284 (s, 2H), 3.864 (s, 9H). ^13^C NMR (151 MHz, DMSO-*d*6): δ 158.72, 148.50, 148.41, 134.63, 125.79, 125.15, 115.62, 115.53, 113.03, 110.90, 105.28, 105.04, 100.85, 56.41, 55.96, 48.21. HRMS (ESI) m/z: (M + H)^+^ calcd for C_23_H_23_N_3_O_4_ 406.1761; found 406.1762.

*N-(4-(tert-butyl)benzyl)-1-(4-methoxyphenyl)-1H-benzo[d]imidazol-5-amine (**13f**)*. Compound **13f** was synthesized according to the general procedure, replacing the corresponding aromatic aldehyde with p-t-butylbenzaldehyde. Compound **13f** was obtained as a yellow solid in a yield of 95%. Mp 175.8–176.9 °C. ^1^H NMR (600 MHz, Chloroform-*d*): δ 7.897 (s, 1H), 7.326–7.3776 (m, 6H), 7.240 (d, 1H, *J* = 9.0), 7.054 (d, 1H, *J* = 2.4), 7. 036 (t, 1H, *J* = 2.4), 7. 025 (t, 1H, *J* = 2.4), 6.706 (dd, 1H, *J* = 6.6), 4.343 (s, 2H), 3.862 (s, 3H), 1.303 (s, 9H). ^13^C NMR (151 MHz, DMSO-*d*6): δ 158.73, 149.33, 145.61, 145.46, 142.63, 137.81, 129.78, 127.49, 126.17, 125.46, 125.16, 115.53, 112.94, 110.97, 100.62, 55.96, 47.34, 34.60, 31.67. HRMS (ESI) m/z: (M + H)^+^ calcd for C_25_H_27_N_3_O 386.2227; found 386.2330.

*N-(4-methoxybenzyl)-1-(4-methoxyphenyl)-1H-benzo[d]imidazol-5-amine (**13g**)*. Compound **13g** was synthesized according to the general procedure, replacing the corresponding aromatic aldehyde with 4-methoxybenzaldehyde. Compound **13g** was obtained as a yellow solid in a yield of 90%. Mp 128.0–129.3 °C. ^1^H NMR (600 MHz, Chloroform-*d*): δ 7.897 (s, 1H), 7.374 (t, 1H, *J*1 = 3.0, *J*2 = 2.4), 7.359 (t, 1H, *J*1 = 2.4, *J*2 = 3.6), 7.325 (d, 1H, *J* = 3.0), 7.311 (d, 1H, *J* = 3.0), 7.235 (d, 1H, *J* = 8.4), 7. 054 (d, 1H, *J* = 2.4), 7.035 (t, 1H, *J*1 = 3.0, *J*2 = 2.4), 7.020 (t, 1H, *J*1 = 2.4, *J*2 = 3.6), 6.871 (t, 1H, *J*1 = 3.0, *J*2 = 1.8), 6.857 (t, 1H, *J*1 = 1.8, *J*2 = 3.0), 6.695 (dd, 1H, *J* = 6.6), 4.303 (s, 2H), 3.861 (s, 3H), 3.785 (s, 3H). ^13^C NMR (151 MHz, DMSO-*d*6): δ 158.73, 158.50, 145.57, 145.46, 142.62, 132.63, 129.78, 128.88, 126.18, 125.15, 115.53, 114.14, 112.98, 110.93, 100.76, 55.96, 55.47, 47.13. HRMS (ESI) m/z: (M + H)^+^ calcd for C_22_H_21_N_3_O_2_ 360.1707; found 360.1705.

*3-(((1-(4-methoxyphenyl)-1H-benzo[d]imidazol-5-yl)amino)methyl)phenol (**13h**)*. Compound **13h** was synthesized according to the general procedure, replacing the corresponding aromatic aldehyde with 3-hydroxybenzaldehyde. Compound **13h** was obtained as a yellow solid in a yield of 91%. Mp 182.9–184.3 °C. ^1^H NMR (600 MHz, Chloroform-*d*): ^1^H NMR (600 MHz, Chloroform-*d*): δ 7.926 (s, 1H), 7.365 (t, 1H, *J*1 = 3.6, *J*2 = 2.4), 7.350 (t, 1H, *J*1 = 2.4, *J*2 = 3.6), 7.226 (d, 1H, *J* = 8.4), 7.185 (t, 1H, *J*1 = *J*2 =7.8), 7. 035 (t, 1H, *J*1 = *J*2 = 2.4), 7. 020 (t, 1H, *J*1 = 3.6, *J*2 = 2.4), 6.928 (d, 1H, *J* = 7.2), 6.781 (dd, 1H, *J* = 6.0), 4.295 (s, 2H), 3.826 (s, 3H). ^13^C NMR (151 MHz, DMSO-*d*6): δ 158.73, 157.88, 145.60, 145.44, 142.64, 142.51, 129.77, 129.67, 126.16, 125.17, 118.23, 115.53, 114.34, 113.93, 112.86, 110.96, 100.62, 55.96, 47.62. HRMS (ESI) m/z: (M + H)^+^ calcd for C_21_H_19_N_3_O_2_ 346.1550; found 346.1547.

*N-(3, 4-dichlorobenzyl)-1-(4-methoxyphenyl)-1H-benzo[d]imidazol-5-amine (**13i**)*. Compound **13i** was synthesized according to the general procedure, replacing the corresponding aromatic aldehyde with 3, 4-dichlorobenzaldehyde. Compound **13i** was obtained as a yellow solid in a yield of 89%. Mp 134.1–135.8 °C. ^1^H NMR (600 MHz, Chloroform-*d*): δ 8.926 (s, 1H), 7.455 (t, 1H, *J*1 = 3.0, *J*2 = 1.8), 7.444 (d, 1H, *J* = 1.8), 7.435 (d, 1H, *J* = 1.2), 7.365 (d, 1H, *J* = 8.4), 7.265 (d, 1H, *J* = 9.0), 7. 220 (dd, 1H, *J* = 7.2), 7.096 (t, 1H, *J*1 = *J*2 = 3.6), 7.081 (t, 1H, *J*1 = 2.4, *J*2 = 3.0), 6.897 (d, 1H, *J* = 9.6), 4.356 (s, 2H), 3.878 (s, 3H). ^13^C NMR (151 MHz, DMSO-*d*6): δ 158.76, 146.23, 145.43, 145.11, 142.77, 129.72, 128.28, 127.82, 127.62, 126.38, 125.79, 125.59, 125.21, 115.53, 112.87, 111.14, 100.75, 55.96, 47.11. HRMS (ESI) m/z: (M + H)^+^ calcd for C_21_H_17_C_l2_N_3_O 398.0821; found 398.0824.

*1-(4-methoxyphenyl)-N-(4-(trifluoromethyl)benzyl)-1H-benzo[d]imidazol-5-amine (**13j**)*. Compound **13j** was synthesized according to the general procedure, replacing the corresponding aromatic aldehyde with 4-(trifluoromethyl)benzaldehyde. Compound **13j** was obtained as a white solid in a yield of 90%. Mp 198.3–200.5 °C. ^1^H NMR (600 MHz, Chloroform-*d*): δ 7.895 (s, 1H), 7.557 (d, 1H, *J* = 8.4), 7.518 (d, 1H, *J* = 7.8), 7.365 (t, 1H, *J*1 = *J*2 = 2.4), 7.350 (t, 1H, *J*1 = 2.4, *J*2 = 3.0), 7.245 (d, 1H, *J* = 8.4), 7.034 (t, 1H, *J*1 = 3.6, *J*2 = 1.8), 7.020 (t, 1H, *J*1 = 1.8, *J*2 = 3.6), 6.974 (d, 1H, *J* = 1.8), 6.695 (dd, 1H, *J* = 6.6), 4.463 (s, 2H), 4.160 (s, 1H), 3.860 (s, 3H). ^13^C NMR (151 MHz, DMSO-*d*6): δ 160.62, 153.55, 151.59, 133.35, 126.92, 126.84, 116.42, 115.69, 115.22, 113.81, 112.29, 112.05, 56.20, 55.74. HRMS (ESI) m/z: (M + H)^+^ calcd for C_22_H_18_F_3_N_3_O 398.1475; found 398.1480.

### 3.2. Determining the Antiviral Activities of Compounds

#### 3.2.1. Cells, Viruses, and Compounds

HEK-293 cells (ATCC, CRL-1573) and 293T cells (ATCC, CRL-3216) were cultured under 5% CO_2_ at 37 °C in Dulbecco’s modified Eagle’s medium with 10% heat inactivated fetal bovine serum (FBS). LASV pseudotyped virus (LASVpv) based on HIV-1 expression system with enhanced green fluorescent protein (EGFP) and firefly luciferase (Luc) encoded in LASV GPC (Josiah strain) were produced by VectorBuilder (Vector Builder Inc., Guangzhou, China). The titers of LASVpv were 8 × 10^8^ TU/mL. Benzimidazole Compounds disclosed herein were synthesized as described in our research.

#### 3.2.2. Cytotoxicity Evaluation

The title compounds were dissolved by DMSO into 20mM solution as stock solution and were stored at −80 °C until being used. The working solutions of the 8 different concentrations (200, 100, 50, 25, 12.5, 6.25, 3.12, 1.56 μM) for the test compounds were serially diluted in the culture medium. HEK-293 cells were inoculated in clear 96-well plates at an approximate density of 2 × 10^4^ cells/well and cultured for 24 h at 37 °C with 5% CO_2_. Then, the working solution of the test compounds was added to each well. PBS was used to wash the dead cells after the plates had been cultured for 4 days.

In this study, the Cell Counting Kit-8 (CCK-8) assay was used to determine the viability of cells, and the optical density (OD) data were used to estimate the survival rate of the cells. The absorbance was measured at 450 nm with a microplate reader. CC_50_ value (50% cytotoxicity Concentration) was calculated using log (inhibitor) vs. response–variable slope in GraphPad Prism 8 software (San Diego, CA, USA).

#### 3.2.3. LASVpv Infections and Inhibition Assays

To test antiviral activities, HEK-293 cells were cultured in opaque white 96-well plates. The assay medium consisted of phenol red-free Dulbecco’s Modified Eagle’s Medium, 2% FBS, 25 mM HEPES, 4.5 g/L D-glucose, and L-glutamine. Cell density and MOI (Multiplicity of Infection) were optimized for the antiviral activity assay in preliminary studies. HEK-293 cells at different densities (5 × 10^3^ to 3 × 10^4^ cells/well) were infected at MOI from 0.5 to 10. 0. The optimized cell density (2 × 10^4^ cells per well) and the dose for LASVpv (MOI = 5) were selected for our assay. A negative control was the addition of only culture medium containing 0.5% DMSO; a positive control was adding virus without compound. 

HEK-293 cells were cultured at a density of 2 × 10^4^ cells per well in opaque white 96-well plates. After incubating overnight, cells were treated with compound solutions of different concentrations (50, 5, 0.5, 0.05, 0.005, 0.0005, 0.00005, 0.000005 μM). Subsequently, HEK-293 cells were infected with LASVpv, and firefly luciferase activity was assayed at 48 h post-infection.

Luciferase signals were detected with Luciferase Assay System (Promega, Madison, WI, USA). A SpectraMax i3X microplate reader (Molecular Devices, San Jose, CA, USA) was used to quantify luminescence using a 1 s read time per well. Visualization and counting of EGFP-positive cells were carried out using the FLoid^®^ Cell Imaging Station. IC_50_ value (50% growth inhibitory concentration) was calculated also using log (inhibitor) vs. response–variable slope in GraphPad Prism 8 software.

### 3.3. Surface Plasmon Resonance (SPR) Studies

Analyses of SPR interactions were performed on a Biacore T200 optical biosensor (GE Healthcare Life Sciences, Chicago, IL, USA). Data were analyzed using BiacoreT200 evaluation software ver 3.2.1 (Chicago, IL, USA). The curves were plotted using Origin 2022 software (Paris, France). The expressing vector pET-22b-His-GST-GP2 was constructed, and GP2 protein was produced by Abiocenter Biotechnology Co., Ltd. (Beijing, China). The phosphate-buffered saline composed of 0.2 M phosphate buffer, 27 mM KCl, 27 mM KCl, 1.37 M NaCl, and 0.5% surfactant P20 were used as the base buffer. Diluted the base buffer 10 times and added 5% DMSO when pH was adjusted to 7.4 as running buffer. The running buffers were used as blank injections, and solvents were used to correct for volume effects.

#### 3.3.1. Ligand Protein Immobilization

The ligand protein GP2 was immobilized on a CM5 chip by standard amine coupling. Using a 1:1 mixture of d 100 mM N-hydroxysuccinimide (NHS) and 100 mM Nethyl-N′-(dimethylaminopropyl)-carbodiimide (EDC), the flow cell surface was activated for 400 s at a flow rate of 10 μL/min. Then, GP2 (50 μg/mL) dissolved in 0.1 M sodium acetate buffer pH 4.0 was immobilized on a CM5 chip for 600 s until the desired immobilization level of 15,783 RU. Finally, the activated carboxyl groups remaining on the surface were blocked by a 400 s injection of methanolamine (pH 8.5).

#### 3.3.2. Screening and Kinetic Analysis

All the binding experiments were performed at 25 °C at a continuous flow rate 10 μL/min with 20 mM phosphate-buffered saline. Compounds were dissolved with DMSO to make 20 mM stock solution and then diluted with the prepared PBS-P^+^ running buffer to fit the concentration (50, 25, 12.5, 6.25, 3.12, 1.56, 0.78, 0.39, 0.19 μM) for the assay. Analytes solutions with a series of increasing concentrations (0.19–50 µM) were applied to channel F1 in SPR-binding buffer at a 10 µL/min flow rate at 25 °C. For reference purposes, one of the flow channels F2 was left untouched (immobilized blankly). The contact and dissociation times were kept at 90 s, and data were double-referenced with both reference cell RU values and zero concentration (5% DMSO) signals.

All equilibrium dissociation constant (K_D_) analyses were performed using Biacore T200 evaluation software version 3.2.1, and the data were fitted to a 1:1 binding.

## 4. Conclusions

Viral hemorrhagic fever caused by LASV is an infectious disease that seriously threatens human health. No specific anti-LASV drug is currently licensed for clinical use. GPC, the glycoprotein complex of LASV, plays a key role in its invasion process and can be used as a target for anti-LASV drugs. Research on envelope glycoprotein inhibitors could help to discover effective therapeutic agents against LASV.

In this study, we prepared two series of novel benzimidazole derivatives targeted at the LASV GPC. Their antiviral activities were tested through pseudovirus bioassays and SPR technical analysis, in which some compounds showed submicromolar anti-LASV activities. Preliminary structure–activity relationship studies found that although 5-amino-benzo[d]imidazole derivatives have lower antiviral activities than series I compounds, their cytotoxicity is also lower in HEK-293 cells, resulting in a higher SI and better safety profile. The presence of subamine bonds in series II compounds generally leads to their higher stability relative to the carbon–carbon double bonds in series I compounds. Substituents with lipophilicity and those that are spatially larger, such as naphthylphenyl, ethoxyphenyl, tert-butylphenyl, 3, 6-dimethoxyphenyl, and 2, 4, 5-trimethoxyphenyl, resulted in the better inhibitory activities of the compounds against LASV, indicating that the antiviral activity is closely related to the lipophilic nature of the para-substituted phenyl group. Additionally, the R_1_ site in series I and the R_2_ site in series II can accommodate larger substituents, which indicates that the group with larger spatial site resistance is a better choice at this position. As a result, potent antiviral compounds were identified. In particular, the IC_50_ values of **7d−Z**, **7h−Z**, **13c**, **13d**, and **13f** showed activities comparable to the positive control compound, **LHF−535**, against LASVpv. However, the cytotoxicities of five representative compounds was investigated on the human embryonic kidney cell line (HEK-293), and the results showed that these compounds had lower toxicities in this study. The SI values of these compounds (1251, 2494, 4798, 9761, and 8602, respectively) were all higher than **LHF−535** (1208). Among them, it is worth pointing out that compound **7h−Z**, with a large conjugated system by introducing naphthalenyl groups, showed the best antiviral activity (IC_50_ = 7.58 nM) with a 2-fold increase in SI, which can be further investigated as a lead compound. Moreover, in the SPR study, we further determined the kinetic parameters of the title compounds. These five representative compounds showed low k_d_ values (<0.03 s^−1^) and low K_D_ (<8.25 × 10^−7^ M), which expose their long residence time (t = 1/K_d_) on their targets, slower dissociation behavior, and strong binding affinity. In conclusion, introducing lipophilicity and spatially larger substituents was very important for prolonging the residence time and enhancing the binding affinities of the target compound on its target; this may give a larger safety margin. Further investigations are underway to modify the benzimidazole derivatives, aiming to improve the antiviral activity against LASV.

To design and synthesize compounds with potent activities against LASV, the influence of substituents needs to be fully considered. This paper provides some references for the design and synthesis of highly active compounds against LASV. In the future, we will further optimize the molecular structure to obtain excellent candidates for clinical use.

## Figures and Tables

**Table 1:** Inhibition of LASVpv pseudotyped viruses by synthetic compounds **7a−7h(Z/E)**.

**Core**	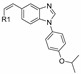			
**Compd.**	**R_1_**	**CC_50_ (μM) ^b^**	**IC_50_ (nM) ^a^**	**SI (CC_50_/IC_50_) ^c^**
**LHF−535**	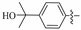	3.68	3.04	1208
**7a−Z**	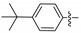	3.01	3.91	770
**7a−E**	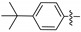	>100	>50	-
**7b−Z**	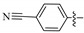	23.58	>50	49
**7b−E**	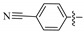	>100	>50	-
**7c−Z**	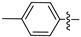	20.93	36.89	567
**7c−E**	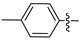	>100	>50	-
**7d−Z**	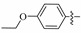	16.97	13.56	1251
**7d−E**	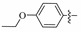	>100	>50	-
**7e−Z**	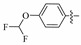	17.88	34.39	520
**7e−E**	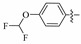	>100	>50	-
**7f−Z**	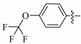	9.47	33.89	279
**7f−E**	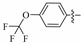	>100	>50	-
**7g−Z**	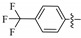	24.38	29.09	838
**7g−E**	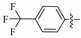	>100	>50	-
**7h−Z**	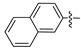	18.92	7.58	2494
**7h−E**	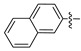	>100	>50	-

^a^ Activity is provided as IC_50_ (nM). ^b^ Cytotoxicity in HEK-293 cells is shown as CC_50_ (μM). ^c^ The selection index is given as SI.

**Table 2:** Inhibition of LASVpv pseudotyped viruses by synthetic compounds **13a−13j**.

**Core**	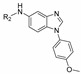			
**Compd.**	**R_2_**	**CC_50_ (μM) ^b^**	**IC_50_ (nM) ^a^**	**SI (CC_50_/IC_50_) ^c^**
**ST−193**	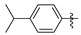	56.29	44.49	1265
**13a**	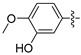	62.60	47.58	1316
**13b**	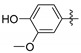	87.28	65.75	1327
**13c**	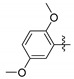	74.18	15.46	4798
**13d**	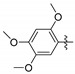	134.80	13.81	9761
**13e**	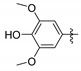	88.78	50.49	1758
**13f**	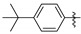	102.10	11.87	8602
**13g**	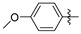	99.96	55.39	1805
**13h**	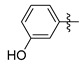	40.34	>100	152
**13i**	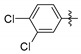	81.73	53.15	1538
**13j**	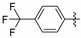	108.80	63.78	1706

^a^ Activity is provided as IC_50_ (nM). ^b^ Cytotoxicity in HEK-293 cells is shown as CC_50_ (μM). ^c^ The selection index is given as SI.

**Table 3:** The SPR data for the binding of active compounds.

Compd.	k_a_ (M^−1^s^−1^)	k_d_ (s^−1^)	K_D_ (M)
**7a−Z**	3.28 × 10^5^	0.04	1.46 × 10^−7^
**7b−Z**	3908	0.28	7.34 × 10^−5^
**7c−Z**	6.56 × 10^4^	0.07	1.18 × 10^−6^
**7d−Z**	8.65 × 10^4^	0.02	3.07 × 10^−7^
**7e−Z**	4.34 × 10^4^	0.08	1.98 × 10^−6^
**7f−Z**	1.56 × 10^4^	0.03	2.19 × 10^−6^
**7g−Z**	5.01	0.13	2.70 × 10^−6^
**7h−Z**	2.94 × 10^5^	0.03	1.29 × 10^−7^
**13a**	1.32 × 10^4^	0.03	2.78 × 10^−6^
**13b**	5.23 × 10^4^	0.20	3.93 × 10^−6^
**13c**	3.95 × 10^4^	0.03	8.25 × 10^−7^
**13d**	5.67 × 10^4^	0.03	6.56 × 10^−7^
**13e**	5.03 × 10^4^	0.22	4.39 × 10^−6^
**13f**	1.78 × 10^5^	0.03	1.73 × 10^−7^
**13g**	6.59 × 10^4^	0.30	4.69 × 10^−6^
**13h**	1.19 × 10^4^	0.42	3.60 × 10^−5^
**13i**	1.94 × 10^4^	0.49	2.54 × 10^−5^
**13j**	8532	0.04	5.49 × 10^−6^
**ST−193**	8.32 × 10^4^	0.01	2.00 × 10^−7^
**LHF−535**	3.04 × 10^5^	0.03	1.14 × 10^−7^

## Data Availability

Not applicable.

## Supplementary Materials

The following supporting information can be downloaded at: https://www.mdpi.com/article/10.3390/molecules28041579/s1, Figures S1–S60: ^1^H and ^13^C NMR spectra of intermediates and final compounds.

## Sample Availability

Samples of the compounds **7a−7h(Z/E)** and **13a−13j** are available from the authors.
