# Supplementary material for: Design, Synthesis, and Biological Evaluation of Benzimidazole Derivatives as Potential Lassa Virus Inhibitors
Source: Molecules. 2023 Feb 7;28(4):1579. doi: 10.3390/molecules28041579 (PMC9963587; doi:10.3390/molecules28041579)
Supplement: Supplementary file 1 [file molecules-28-01579-s001.zip › molecules-2203317-supplementary.pdf]

Supplemental Material

Design, Synthesis, and Biological Evaluation of Benzimidazole Derivatives as Potential Lassa Virus Inhibitors

Jinwei Chen [1,2,†], Likun Xu [2,†], Baogang Wang [2], Dongna Zhang [2], Liangliang Zhao [2], Zhuchun Bei [2] and Yabin Song [1,2,*]

[1] School of Pharmacy, Anhui Medical University, Hefei 230032, China
[2] State Key Laboratory of Pathogen and Biosecurity, Beijing Institute of Microbiology and Epidemiology, Beijing 100071, China
* Correspondence: songyb@126.com or songyabin@bmi.ac.cn
† These authors contributed equally to this work.

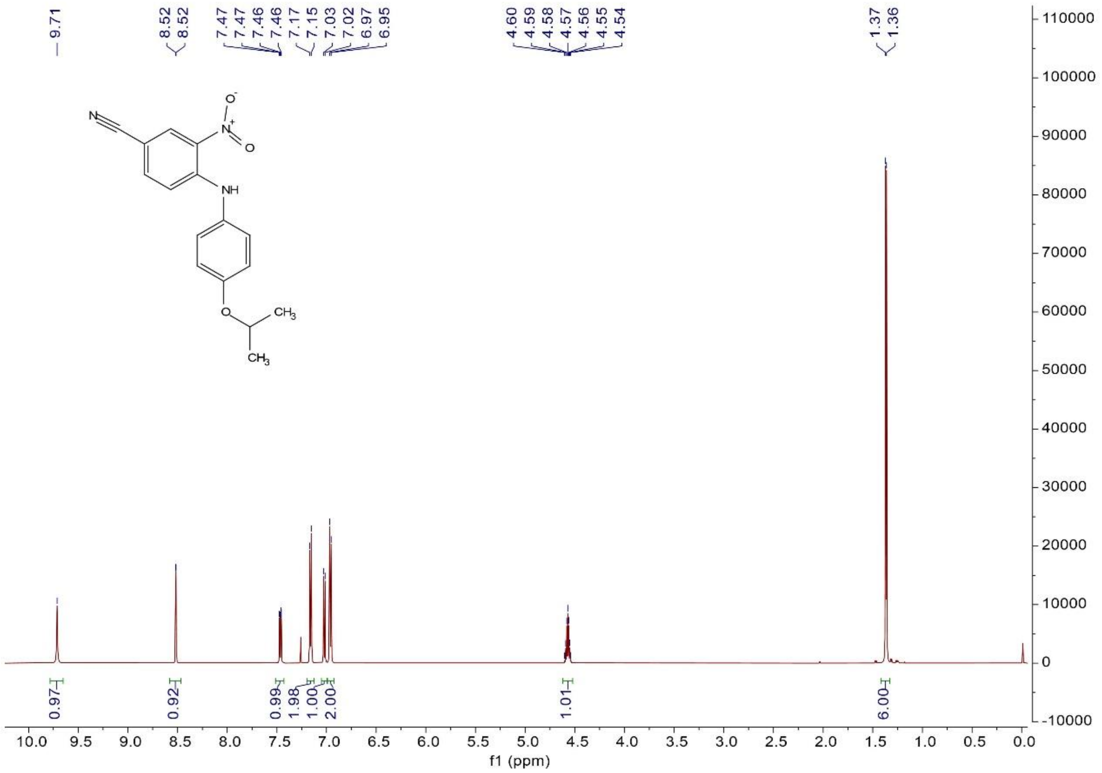

Figure S1. ^{1}H NMR of **3** in $CDCl_3$

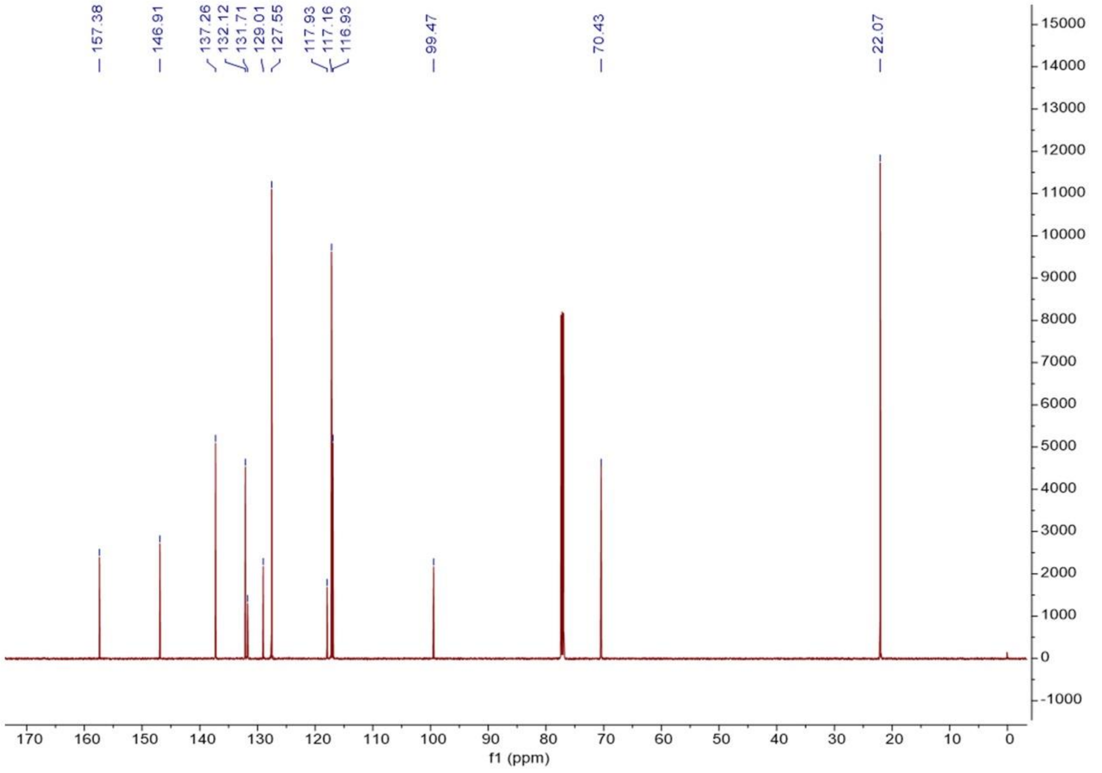

Figure S2. ^{13}C NMR of **3** in $CDCl_3$

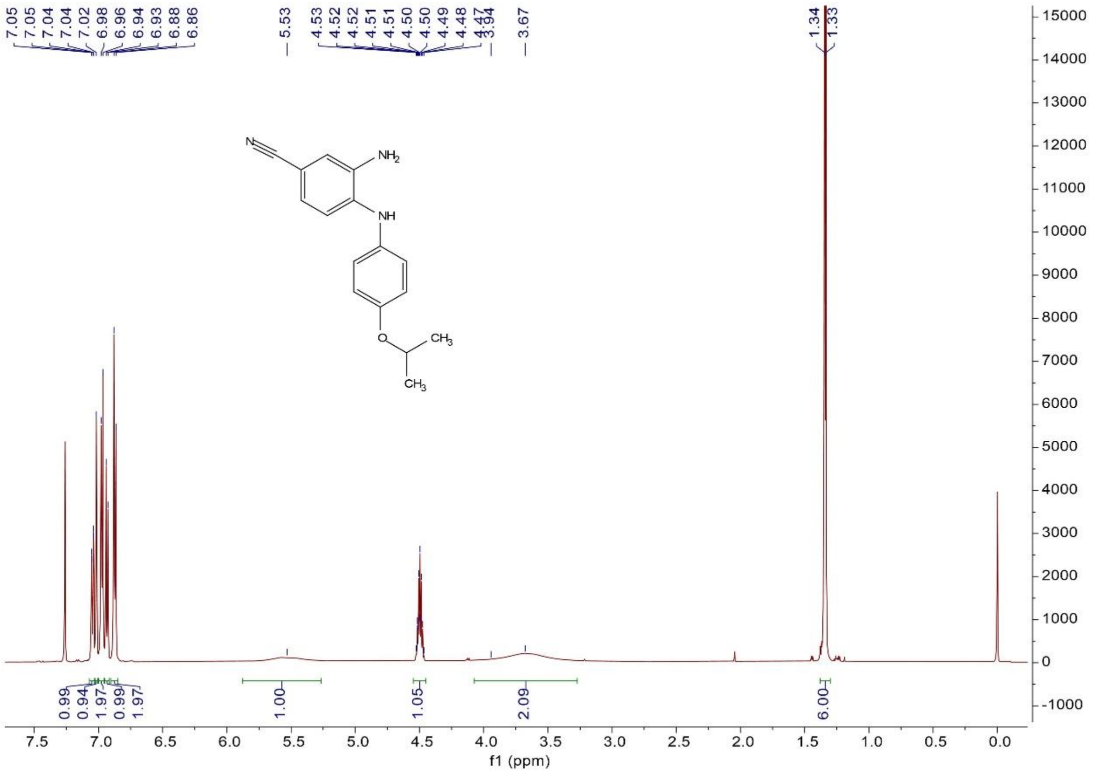

Figure S3. ^{1}H NMR of **4** in $CDCl_3$

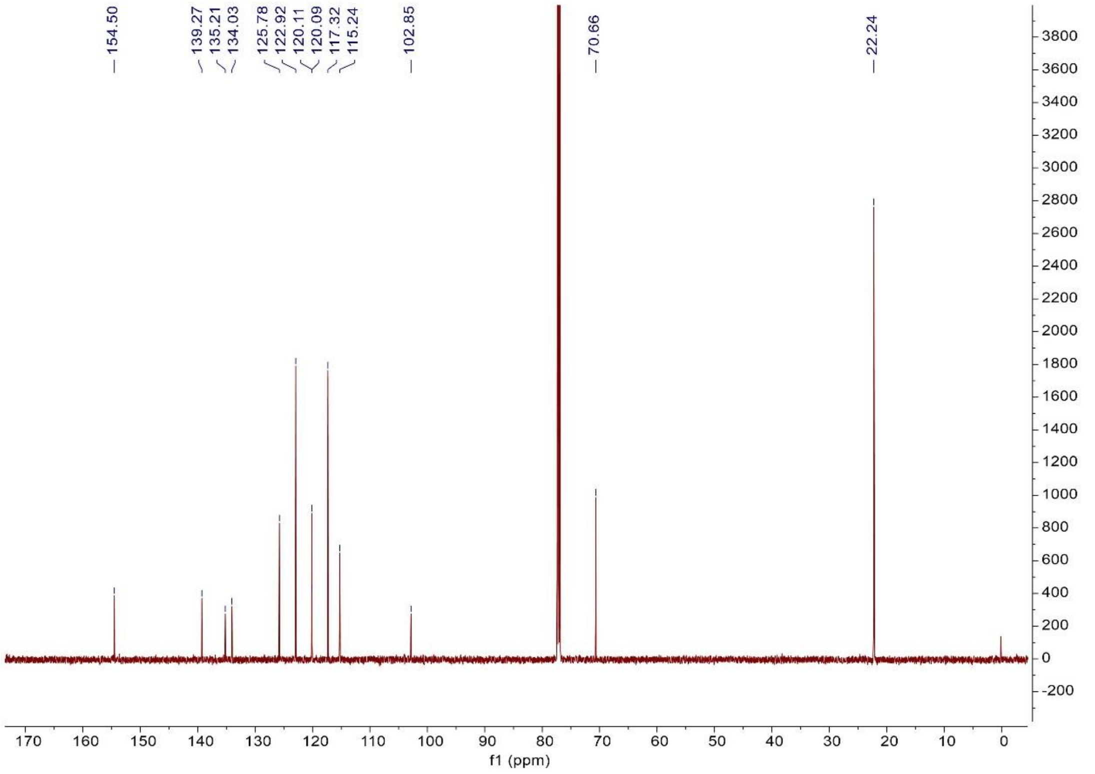

Figure S4. ^{13}C NMR of **4** in $CDCl_3$

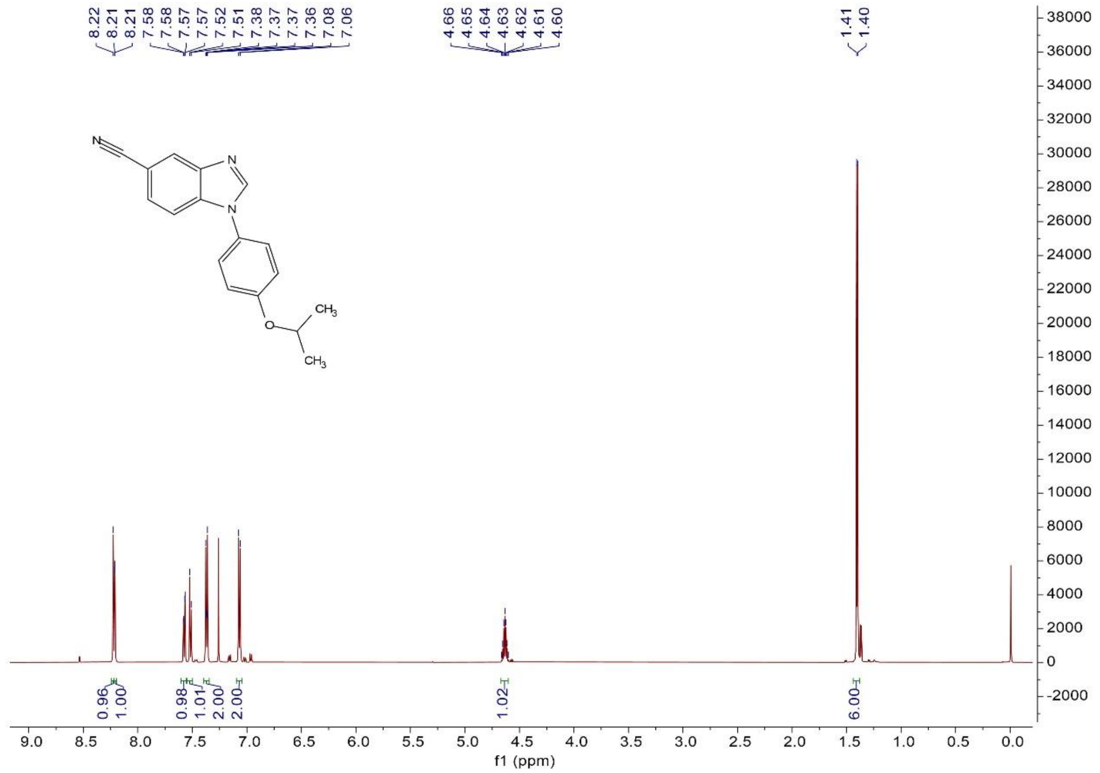

Figure S5. ^{1}H NMR of **5** in $CDCl_3$

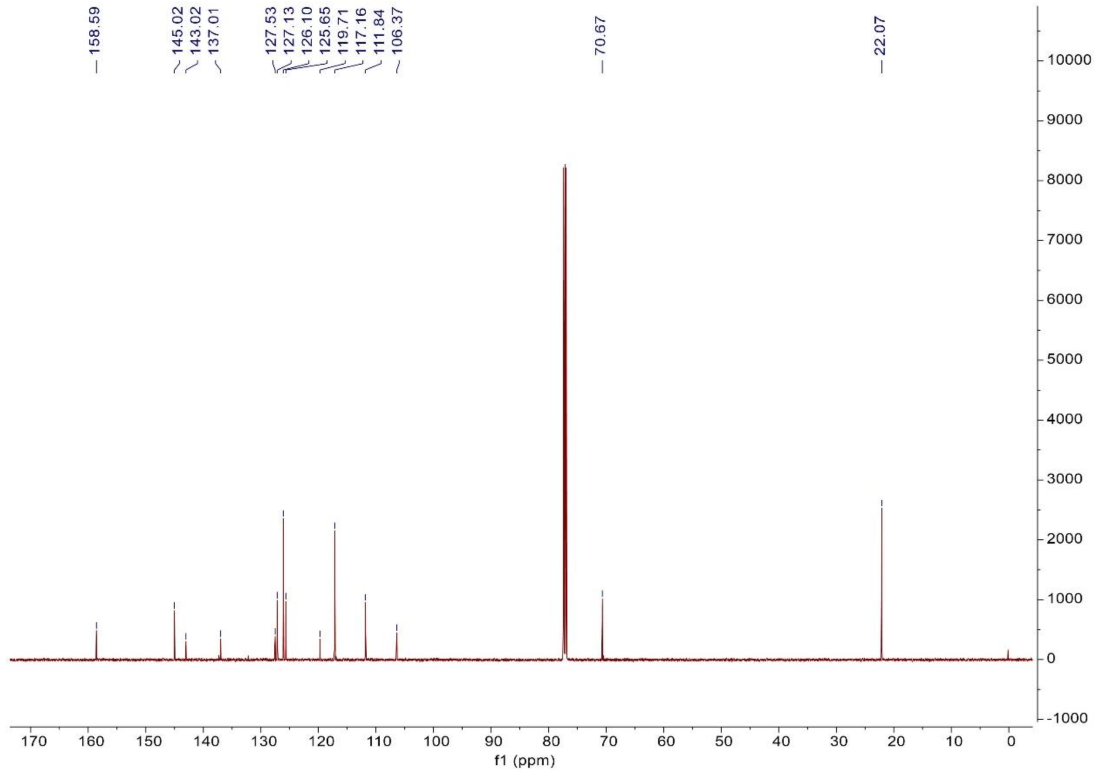

Figure S6. ^{13}C NMR of **5** in $CDCl_3$

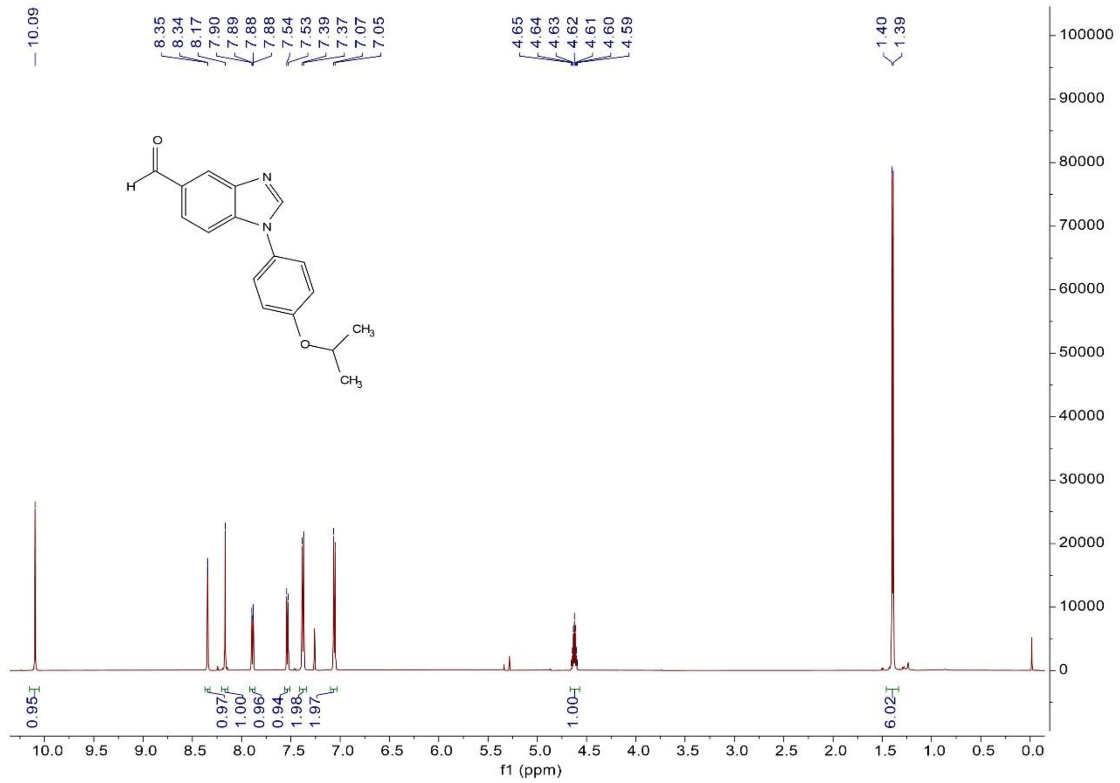

Figure S7. ^{1}H NMR of **6** in $CDCl_3$

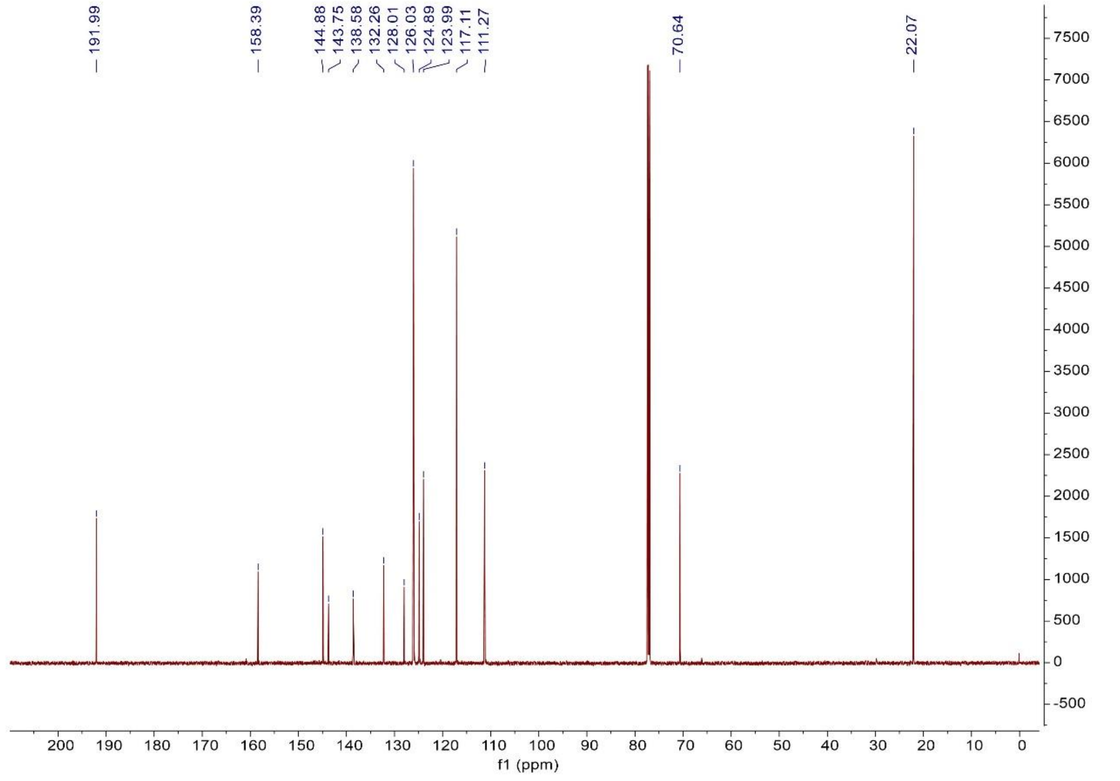

Figure S8. ^{13}C NMR of **6** in $CDCl_3$

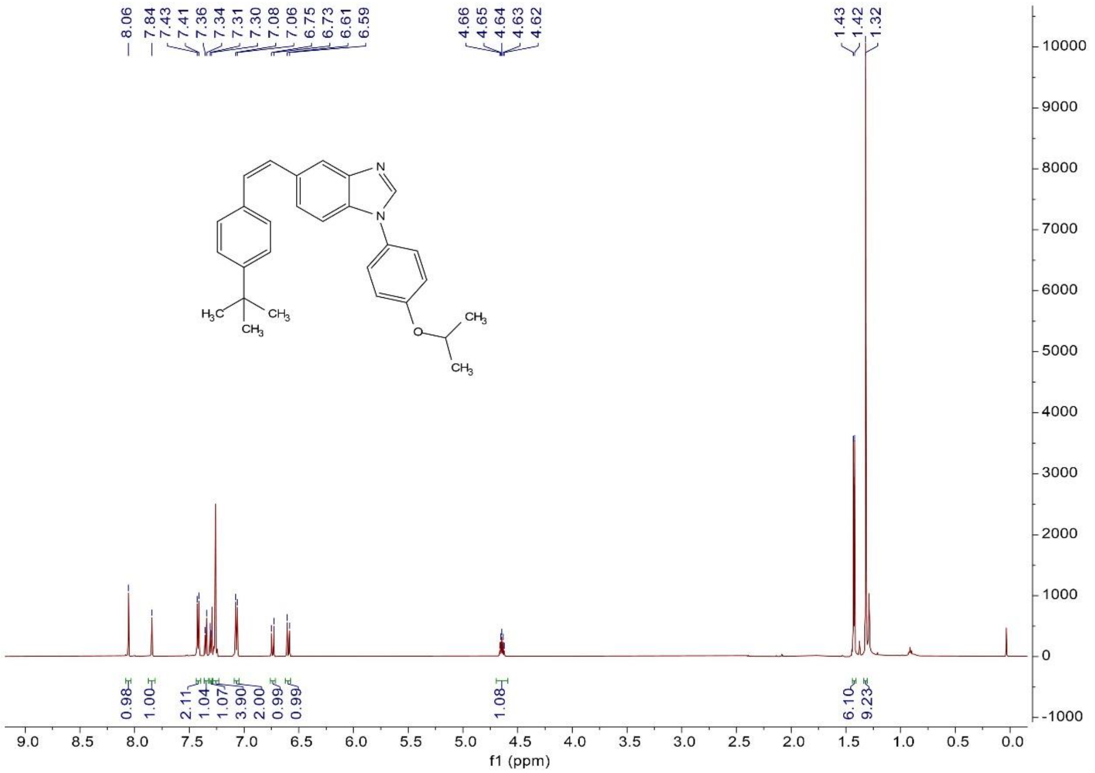

Figure S9. ^{1}H NMR of **7a-Z** in $CDCl_3$

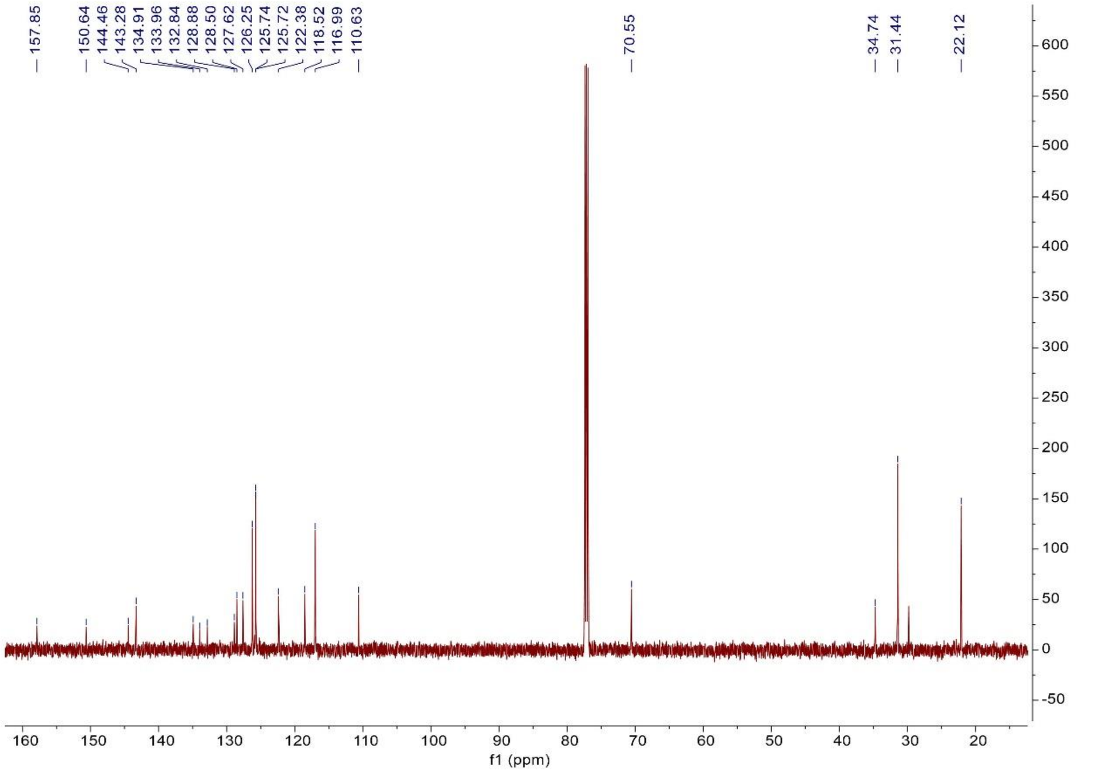

Figure S10. ^{13}C NMR of **7a-Z** in $CDCl_3$

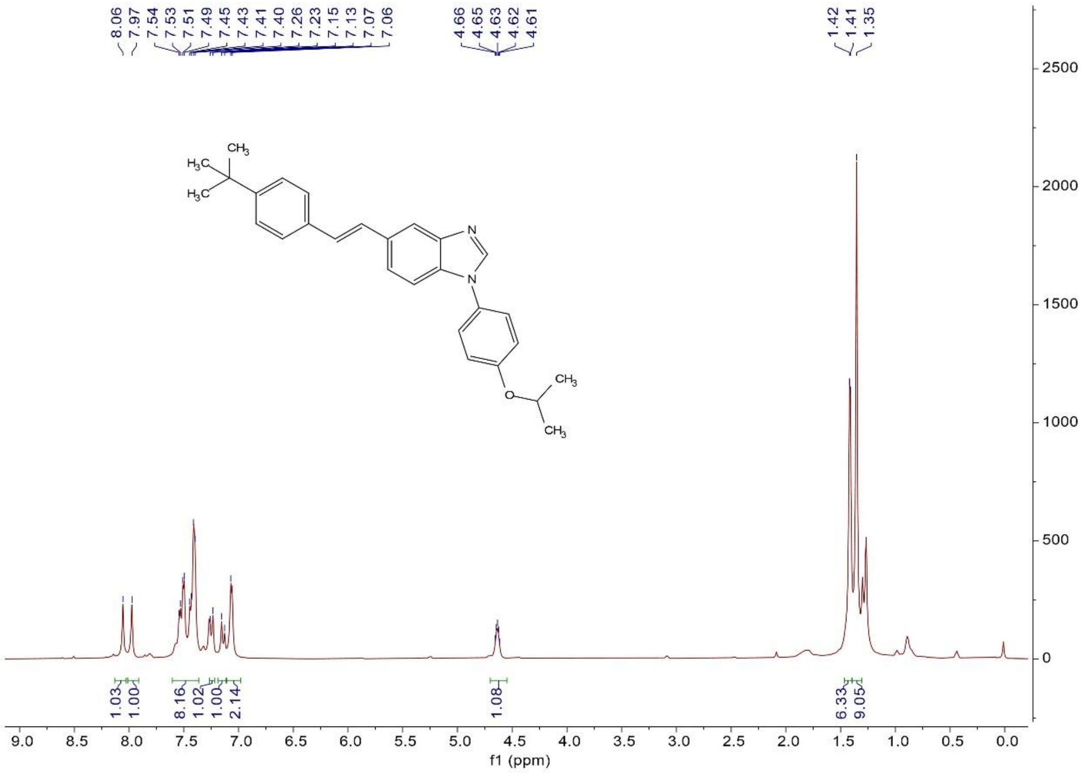

Figure S11. ^{1}H NMR of **7a-E** in $CDCl_3$

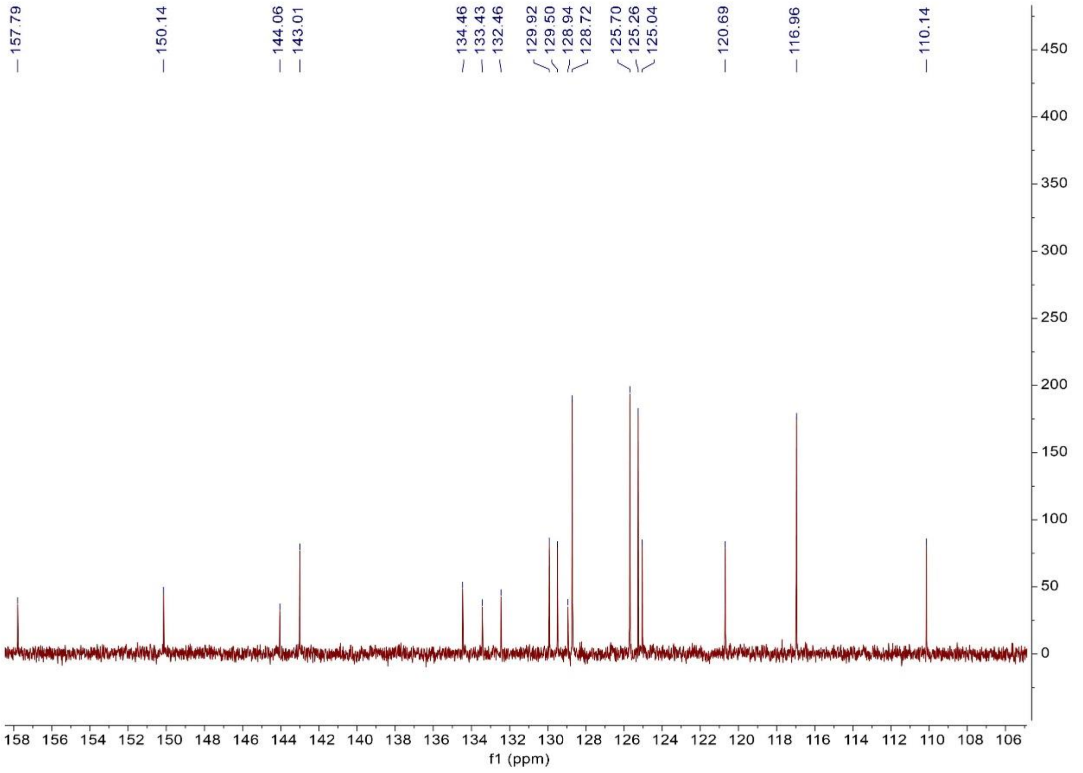

Figure S12. ^{13}C NMR of **7a-E** in $CDCl_3$

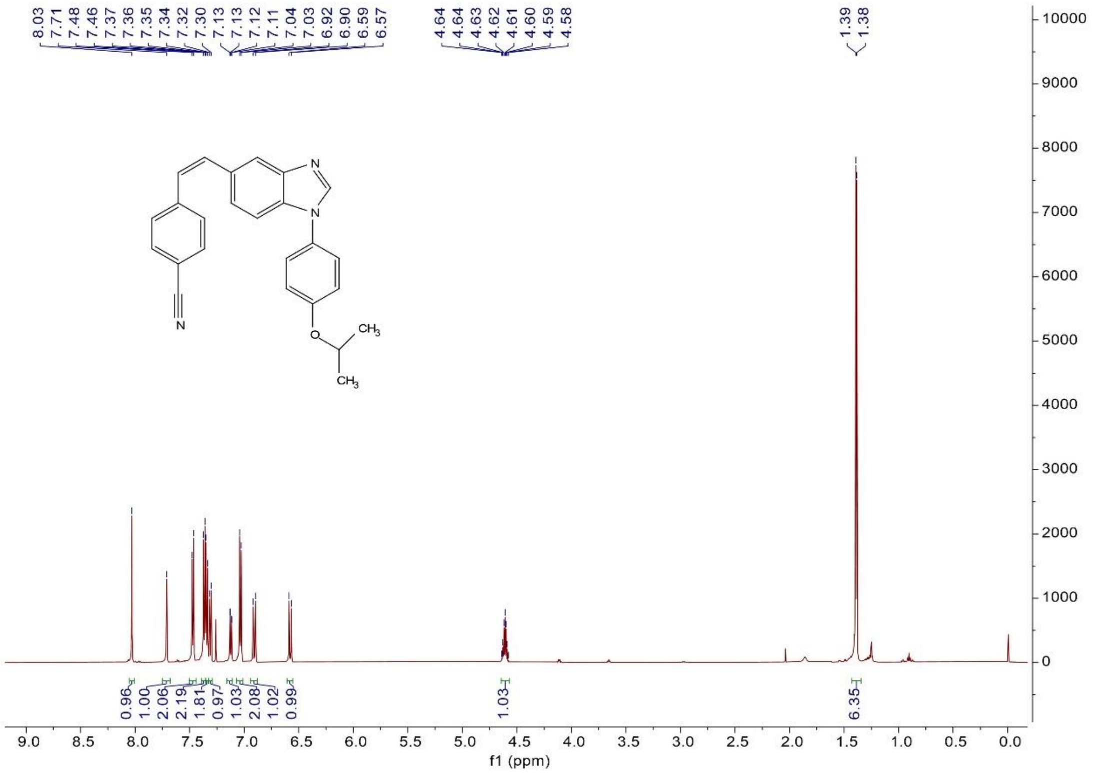

Figure S13. ^{1}H NMR of **7b-Z** in CDCl3

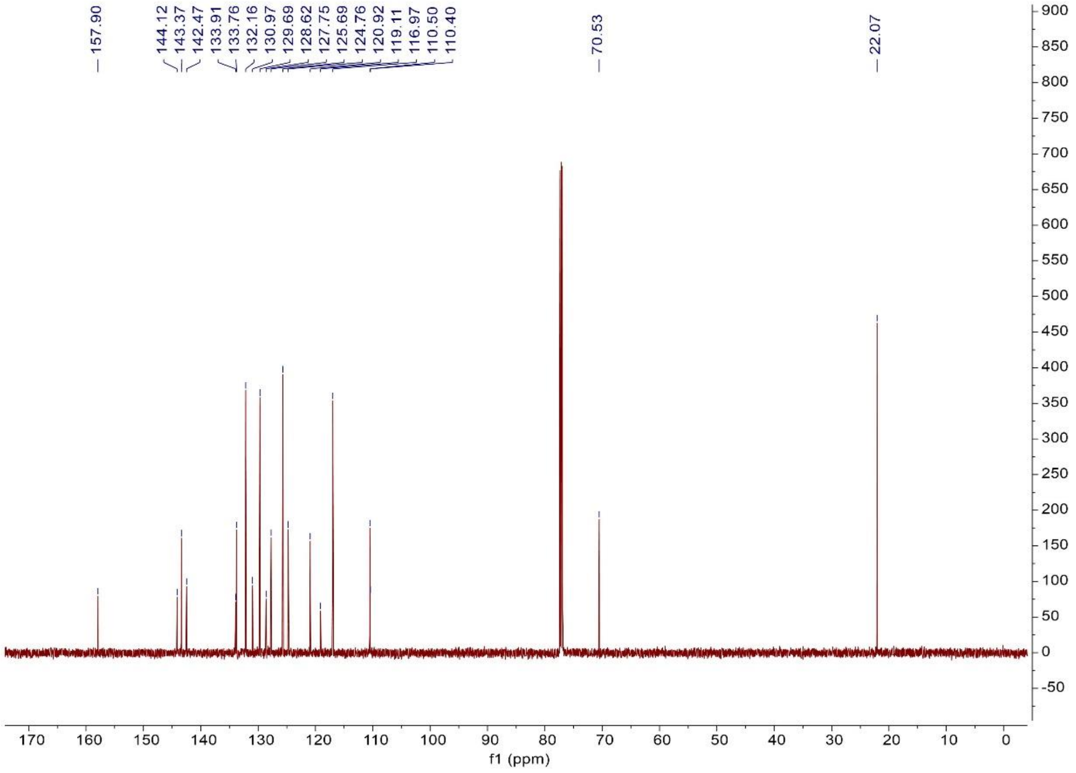

Figure S14. ^{13}C NMR of **7b-Z** in CDCl3

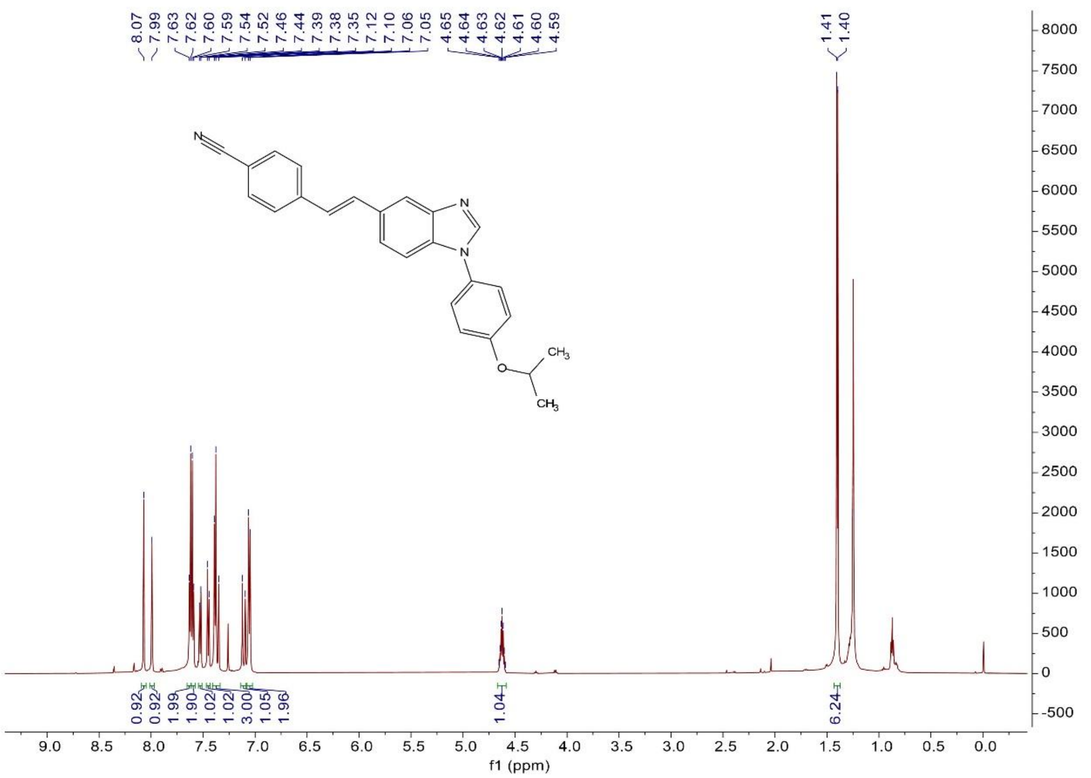

Figure S15. ^{1}H NMR of **7b-E** in $CDCl_3$

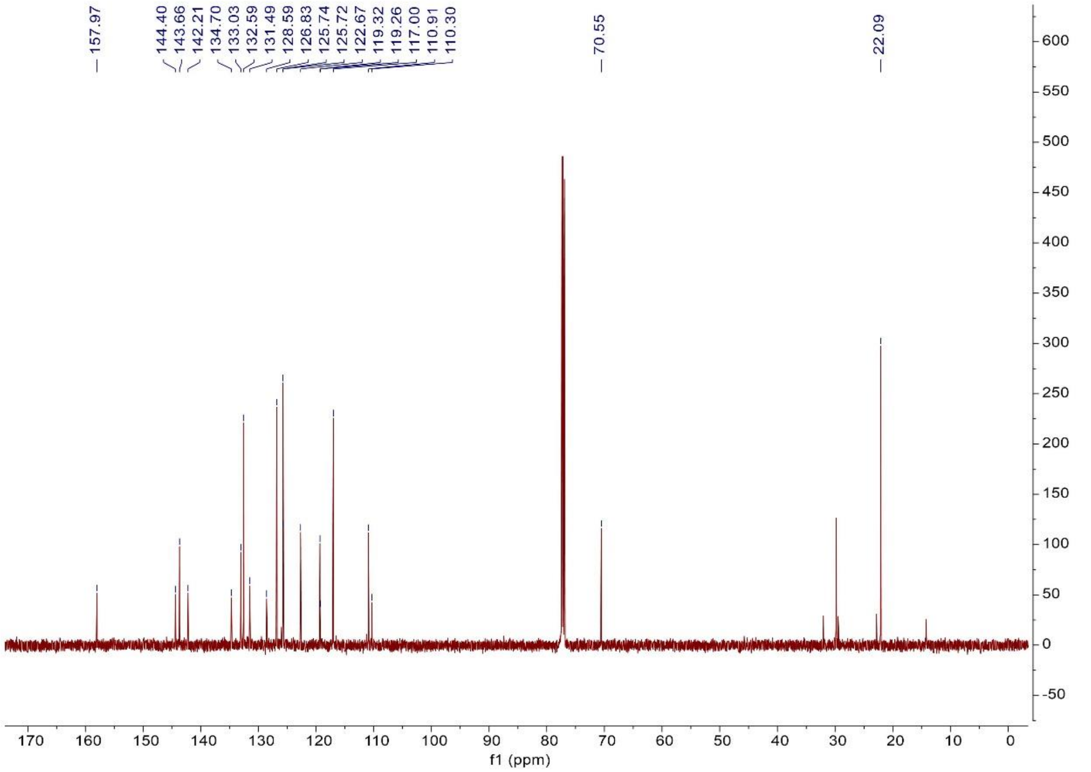

Figure S16. ^{13}C NMR of **7bE** in $CDCl_3$

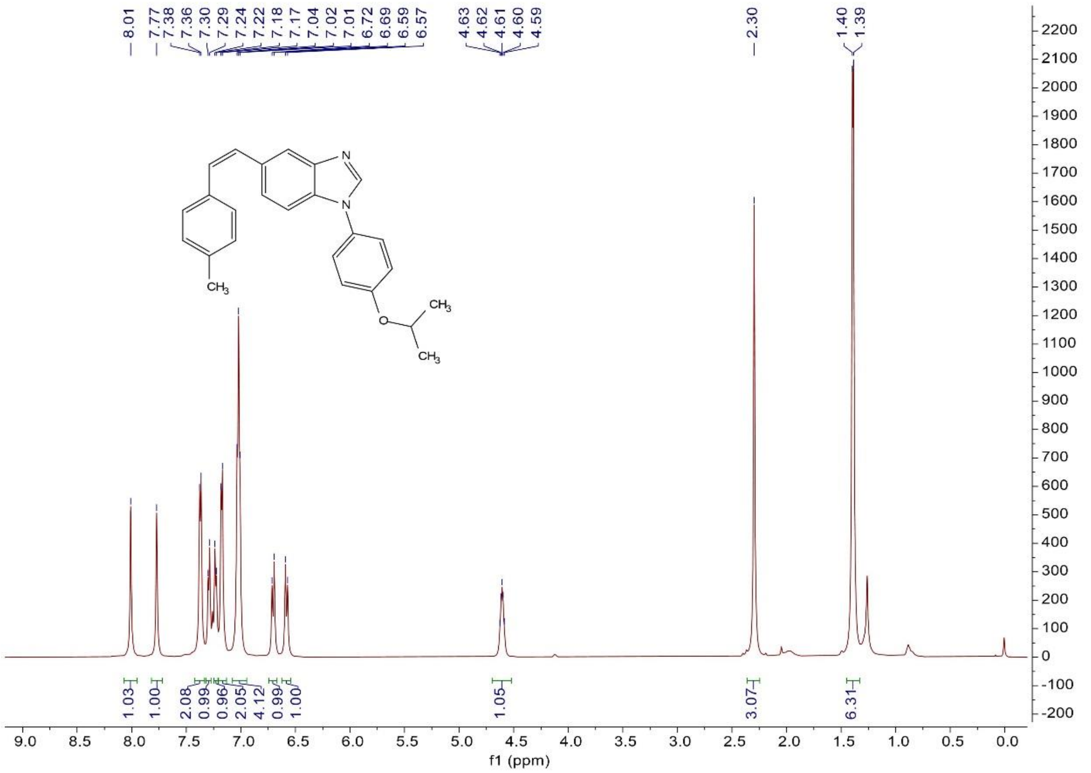

Figure S17. ^{1}H NMR of **7c-Z** in CDCl3

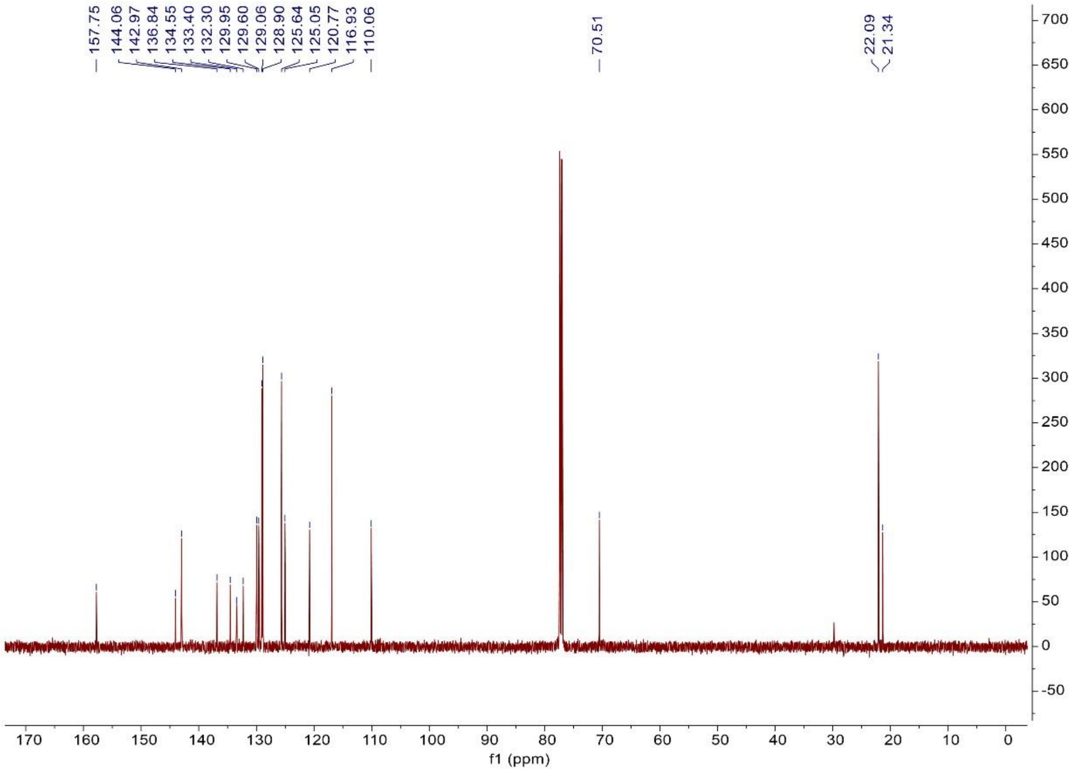

Figure S18. ^{13}C NMR of **7c-Z** in CDCl3

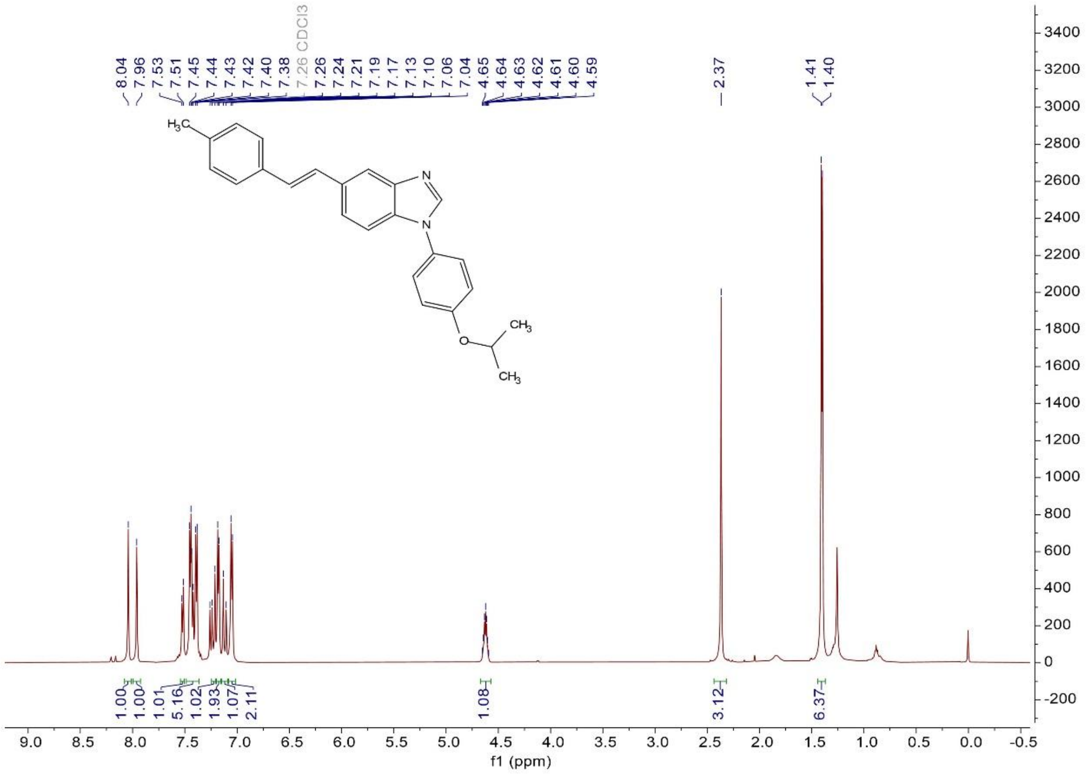

Figure S19. ^{1}H NMR of **7c-E** in $CDCl_3$

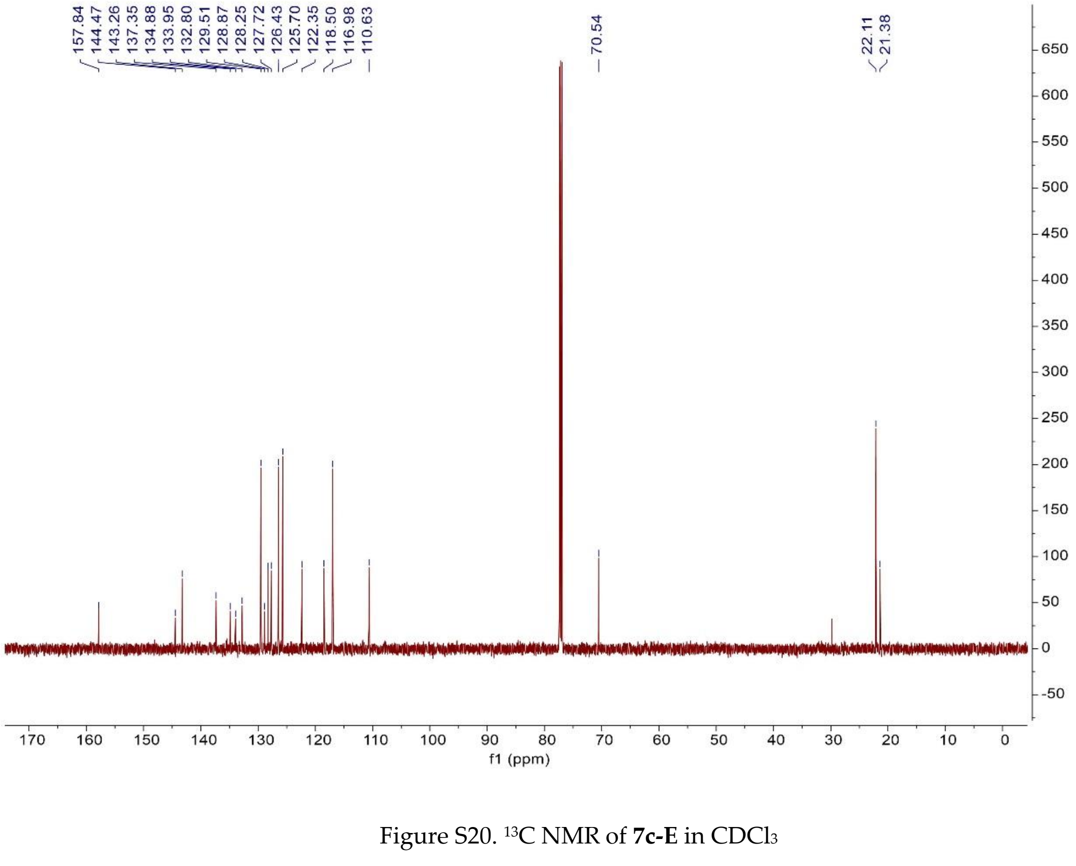

Figure S20. ^{13}C NMR of **7c-E** in $CDCl_3$

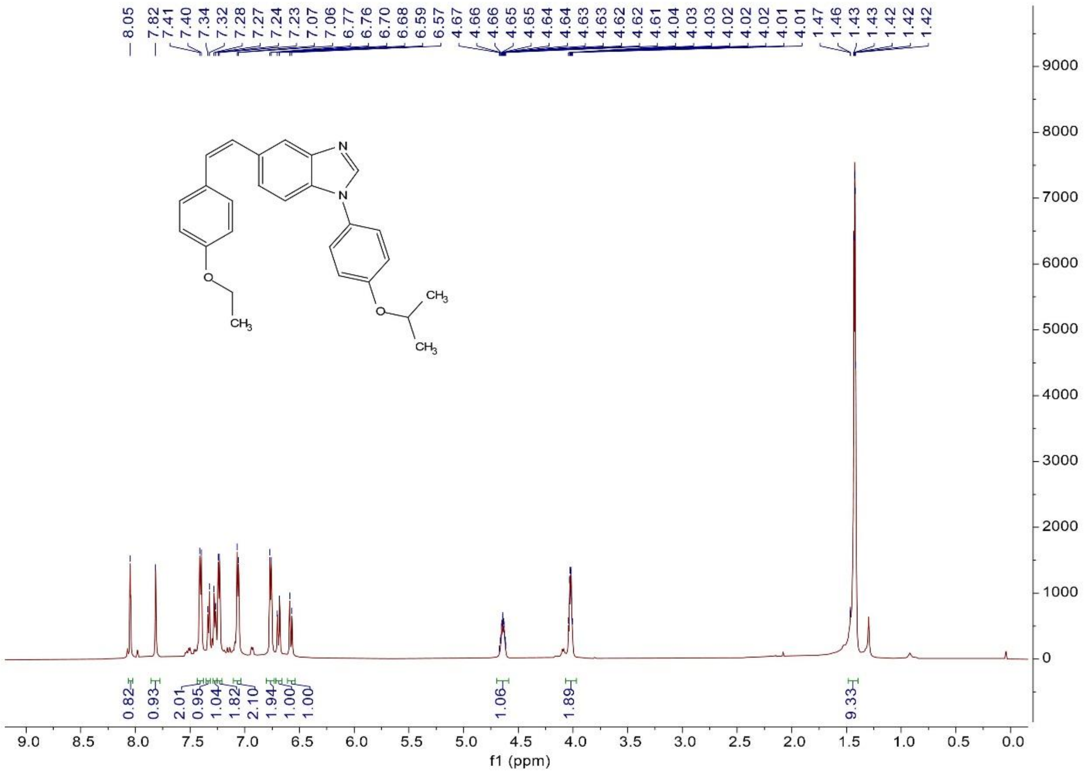

Figure S21. ^{1}H NMR of **7d-Z** in $CDCl_3$

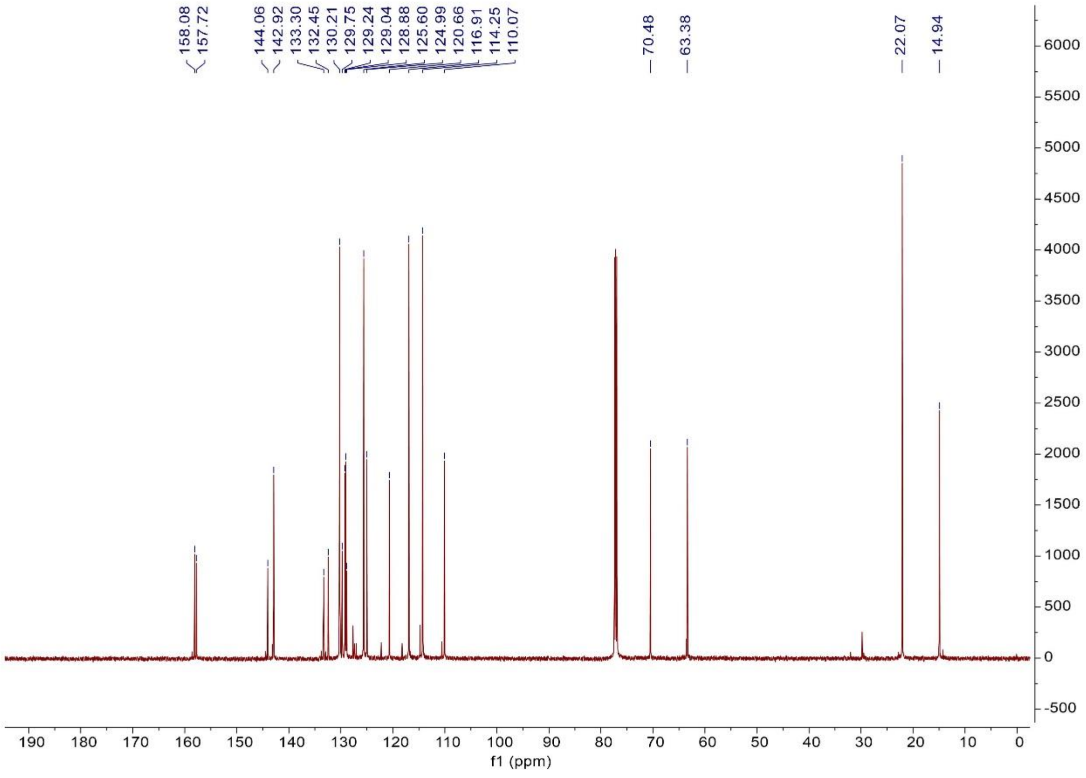

Figure S22. ^{13}C NMR of **7d-Z** in $CDCl_3$

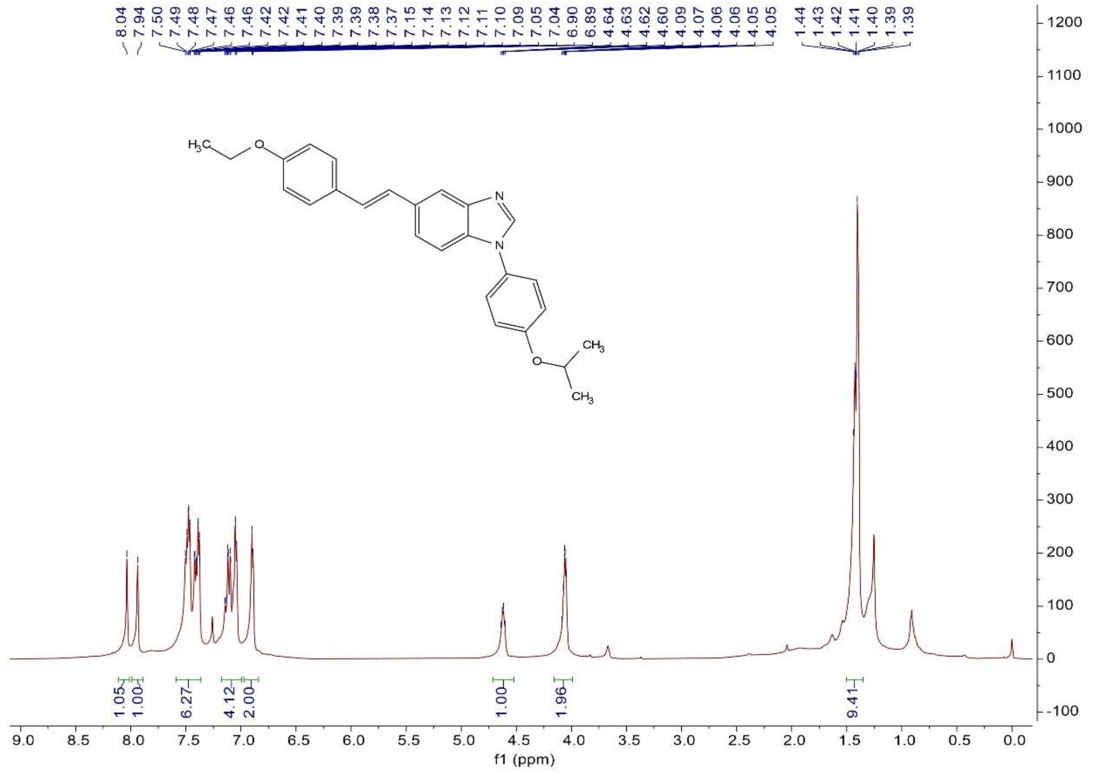

Figure S23. ^{1}H NMR of **7d-E** in $CDCl_3$

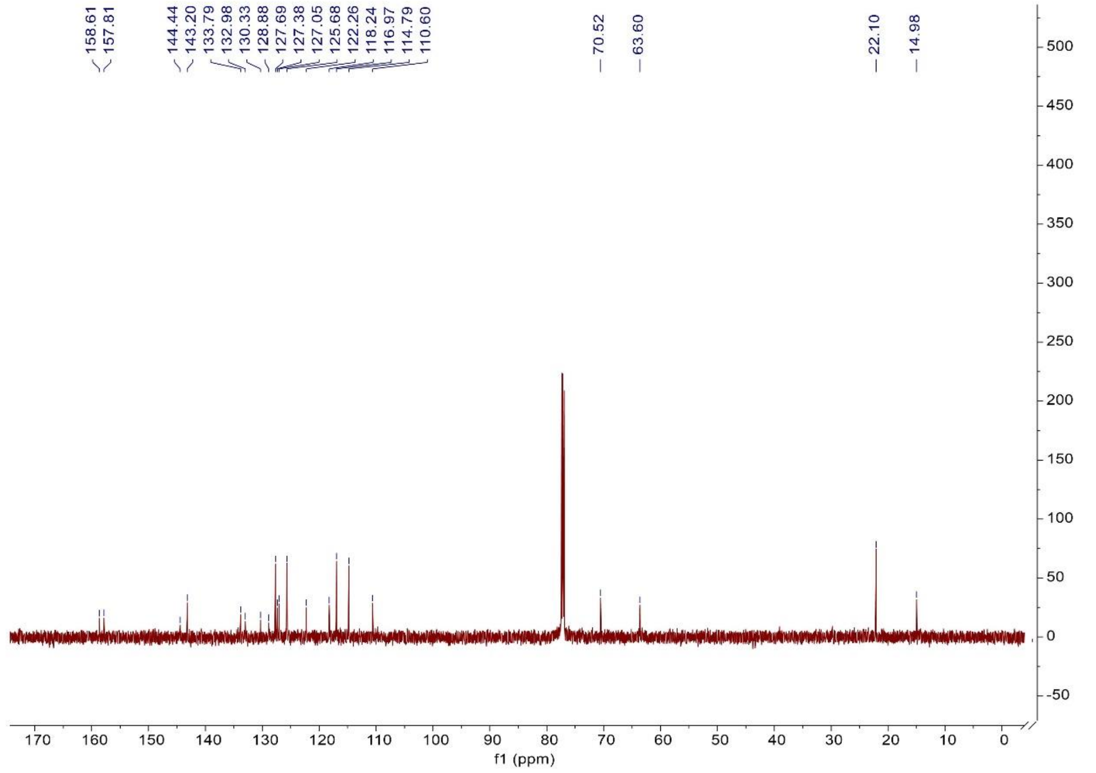

Figure S24. ^{13}C NMR of **7d-E** in $CDCl_3$

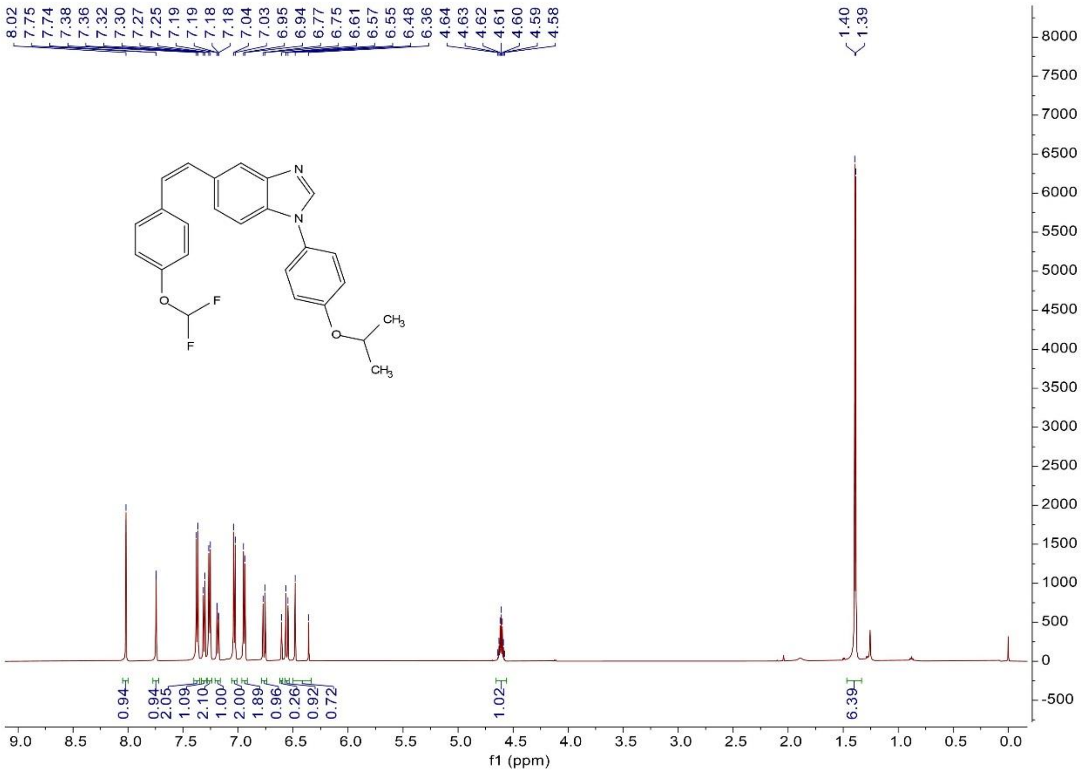

Figure S25. ^{1}H NMR of **7e-Z** in $CDCl_3$

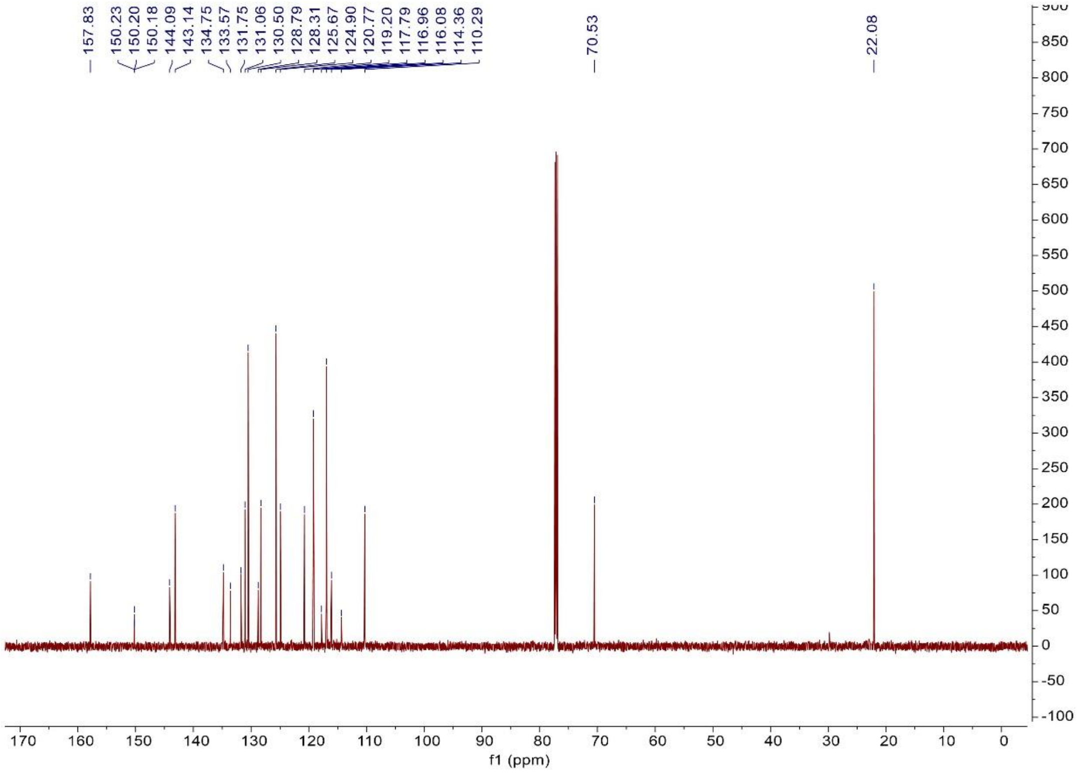

Figure S26. ^{13}C NMR of **7e-Z** in $CDCl_3$

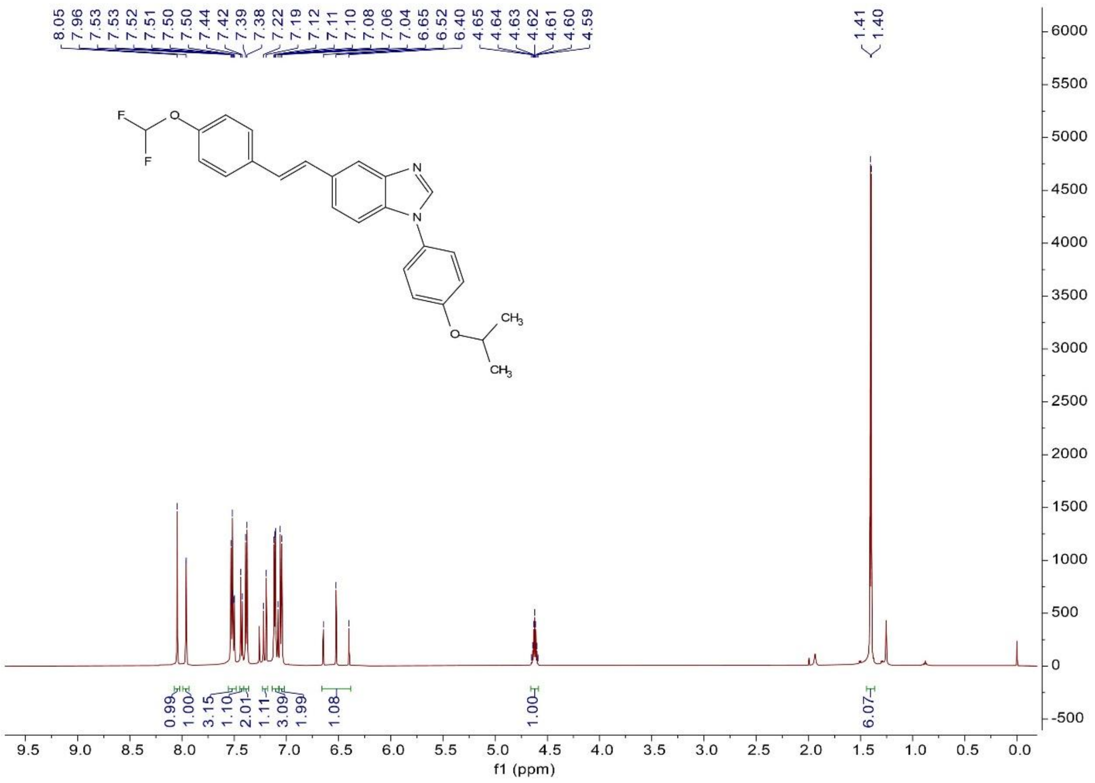

Figure S27. ^{1}H NMR of **7e-E** in $CDCl_3$

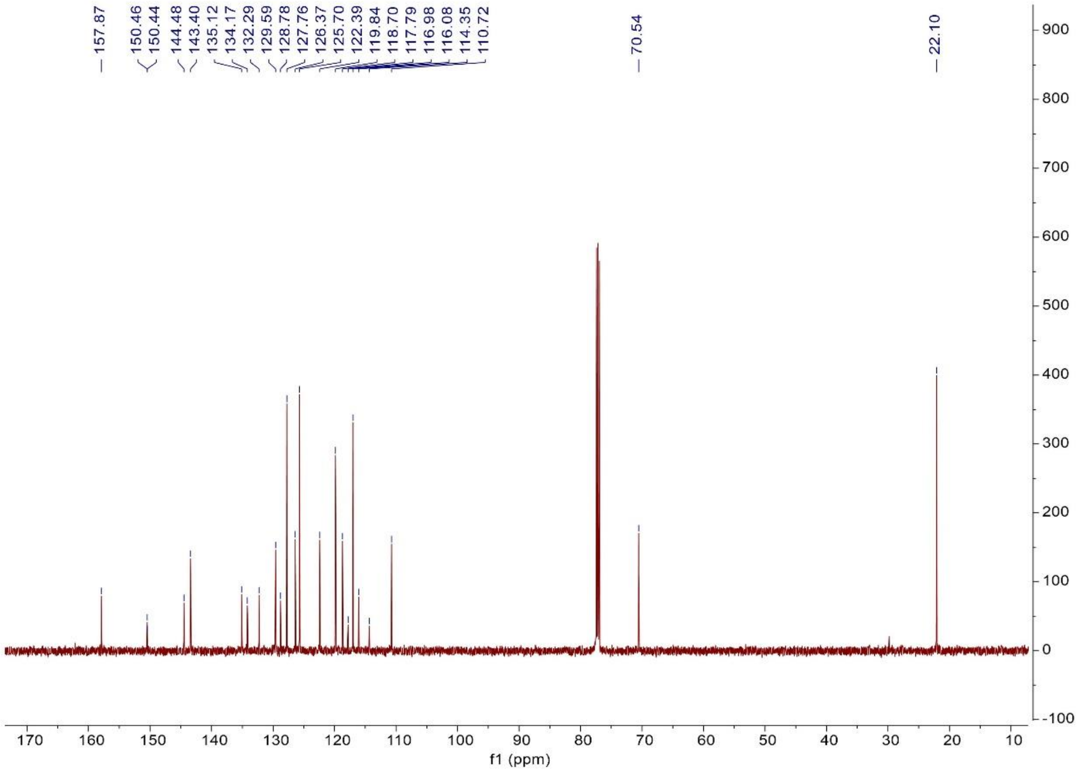

Figure S28. ^{13}C NMR of **7e-E** in $CDCl_3$

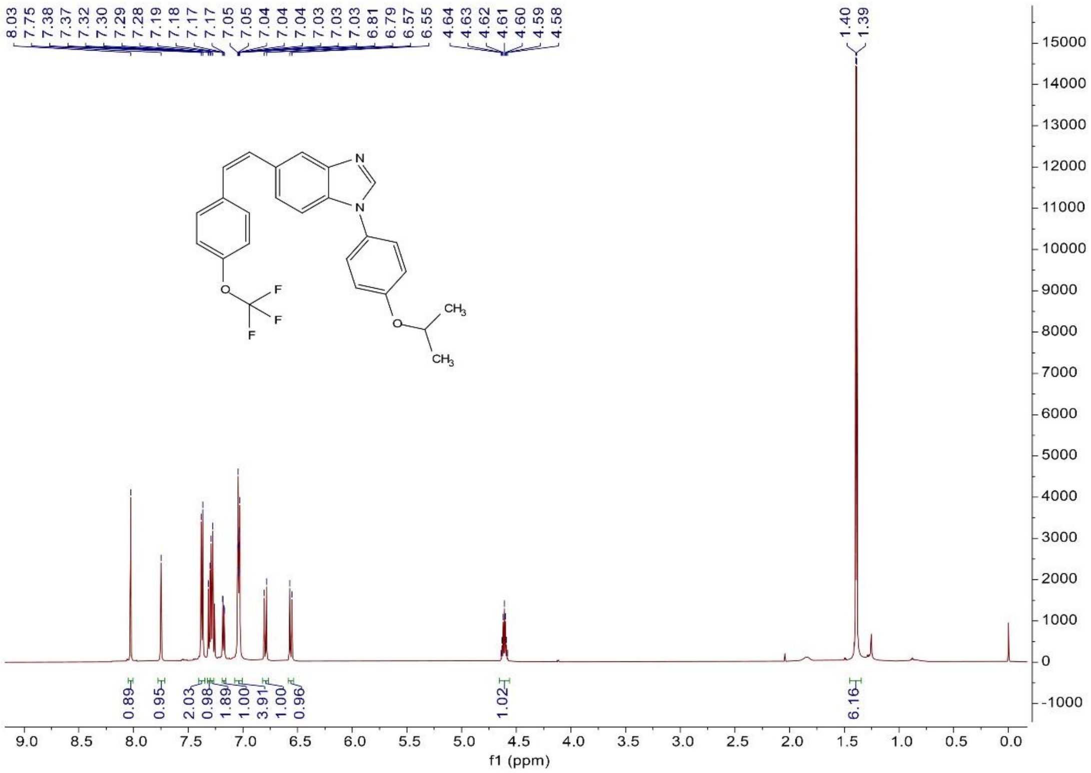

Figure S29. ^{1}H NMR of **7f-Z** in $CDCl_3$

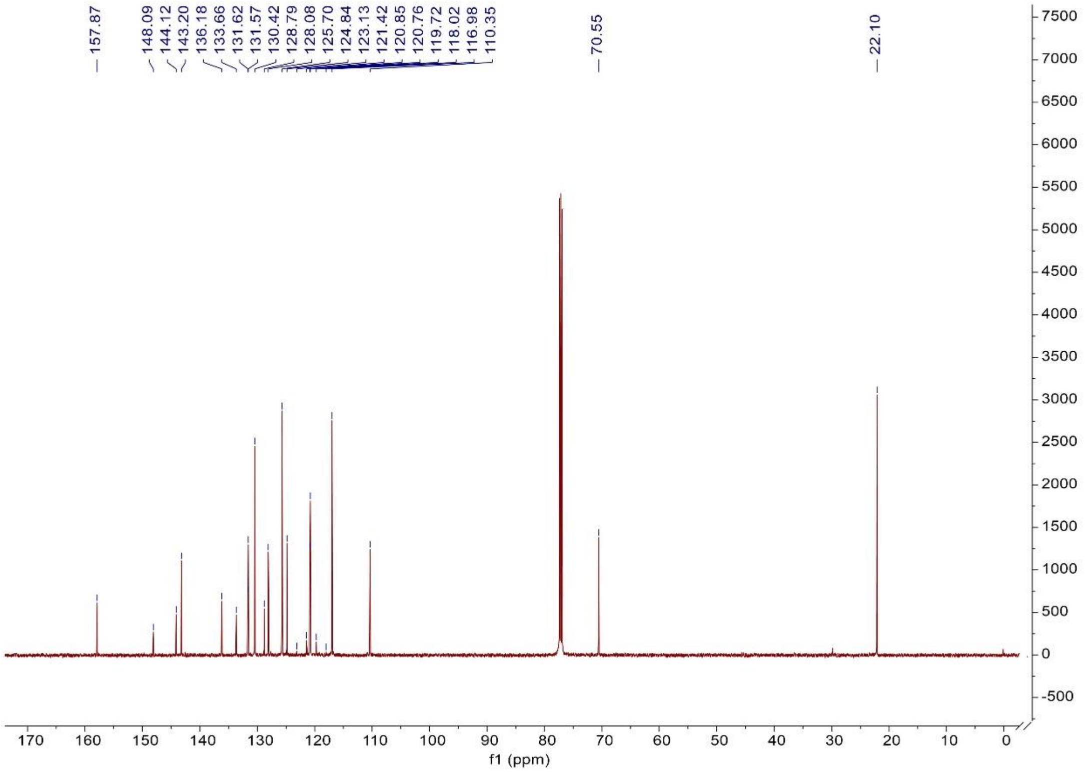

Figure S30. ^{13}C NMR of **7f-Z** in $CDCl_3$

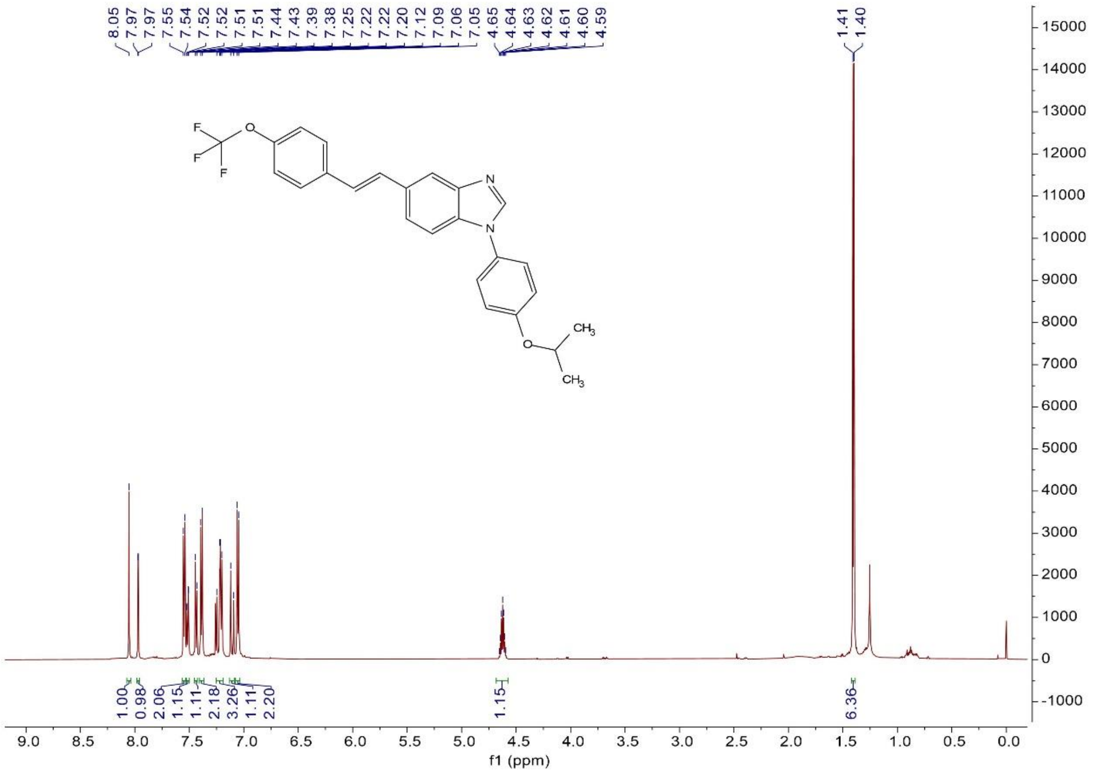

Figure S31. ^{1}H NMR of **7f-E** in CDCl3

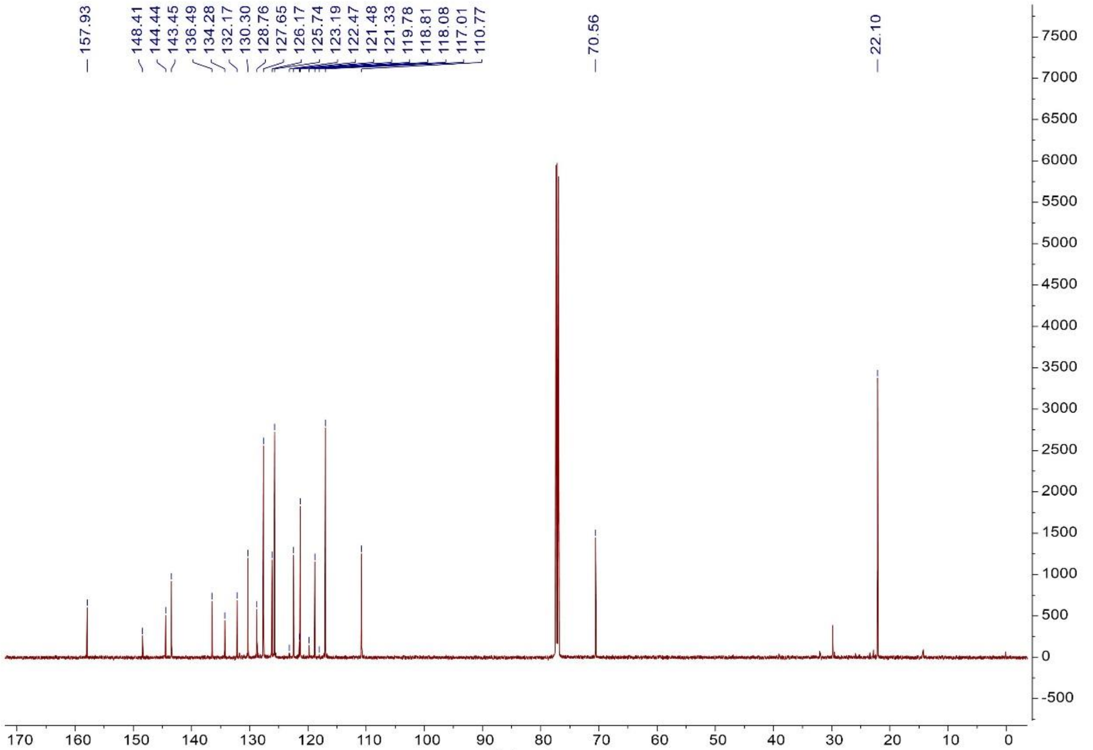

Figure S32. ^{13}C NMR of **7f-E** in CDCl3

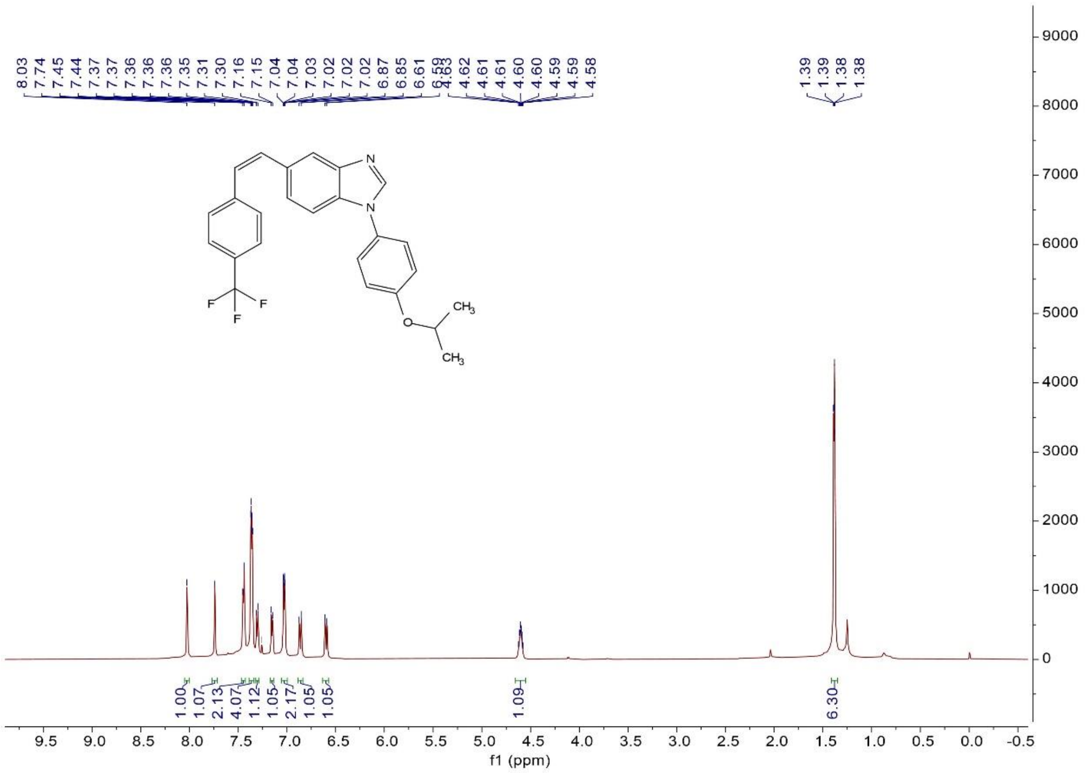

Figure S33. ^{1}H NMR of **7g-Z** in $CDCl_3$

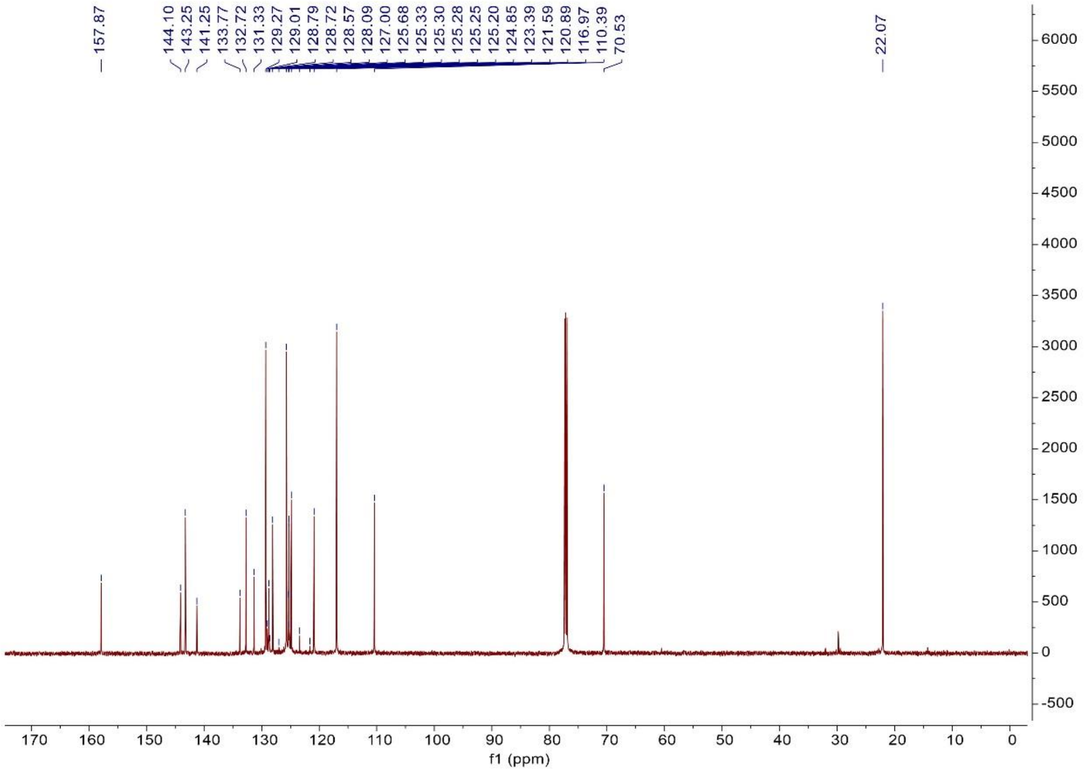

Figure S34. ^{13}C NMR of **7g-Z** in $CDCl_3$

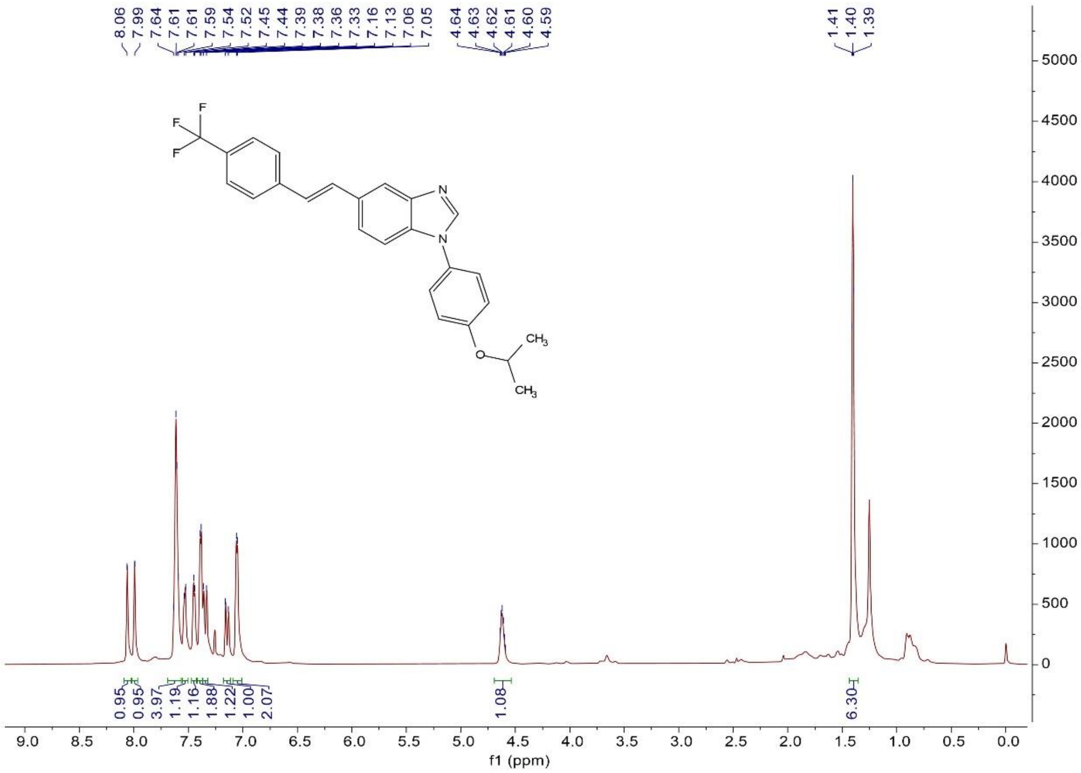

Figure S35. ^{1}H NMR of **7g-E** in $CDCl_3$

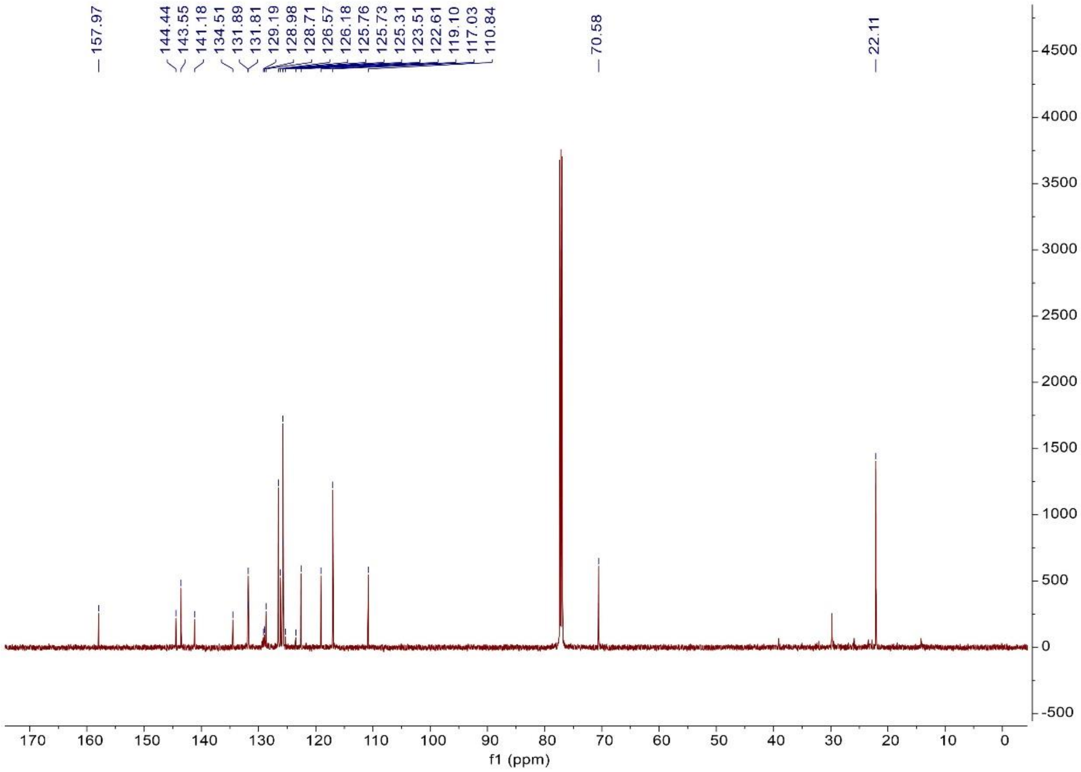

Figure S36. ^{13}C NMR of **7g-E** in $CDCl_3$

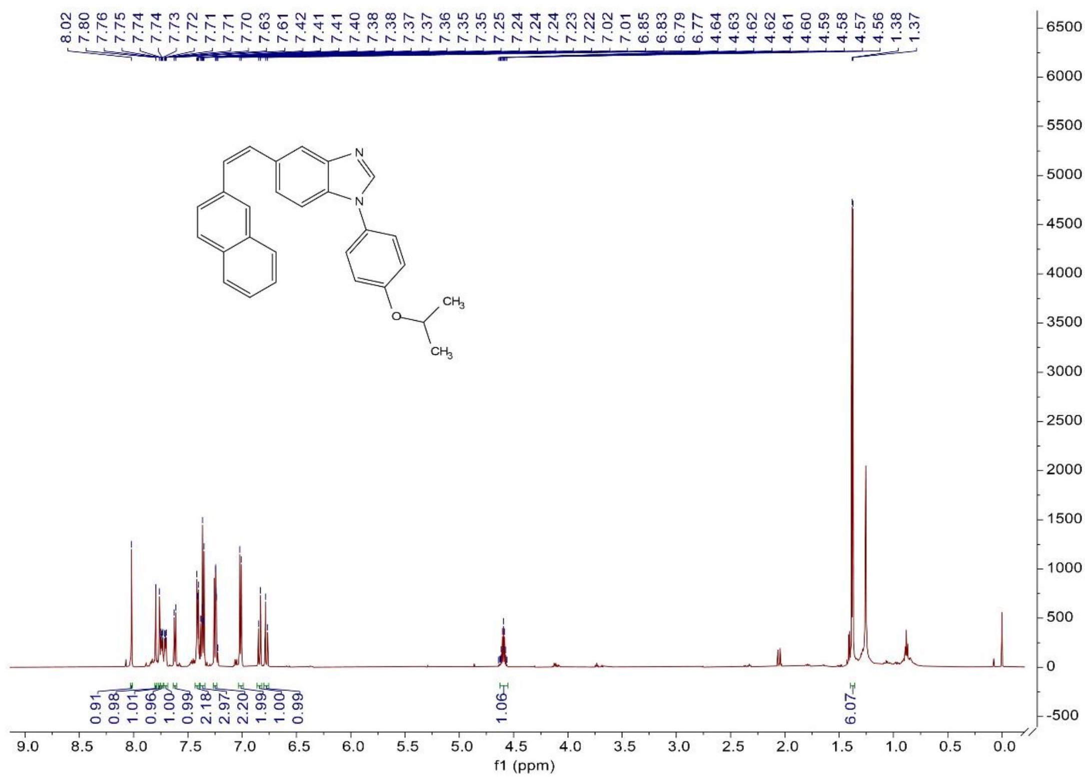

Figure S37. ^{1}H NMR of **7h-Z** in $CDCl_3$

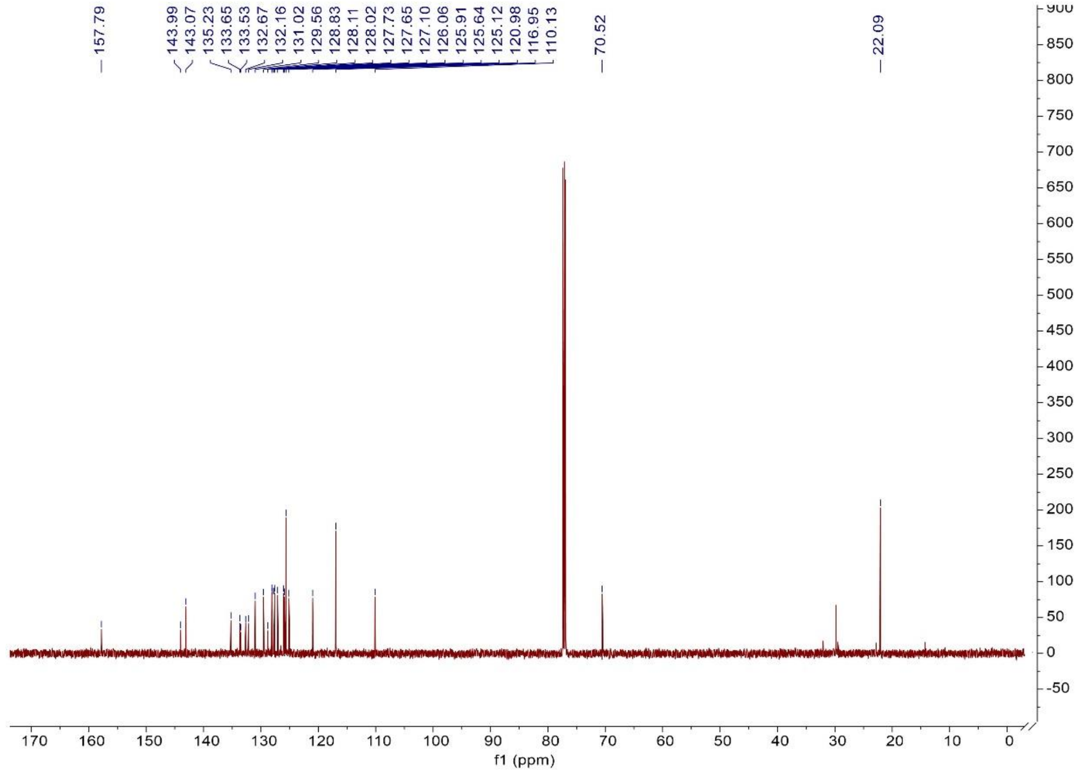

Figure S38. ^{13}C NMR of **7h-Z** in $CDCl_3$

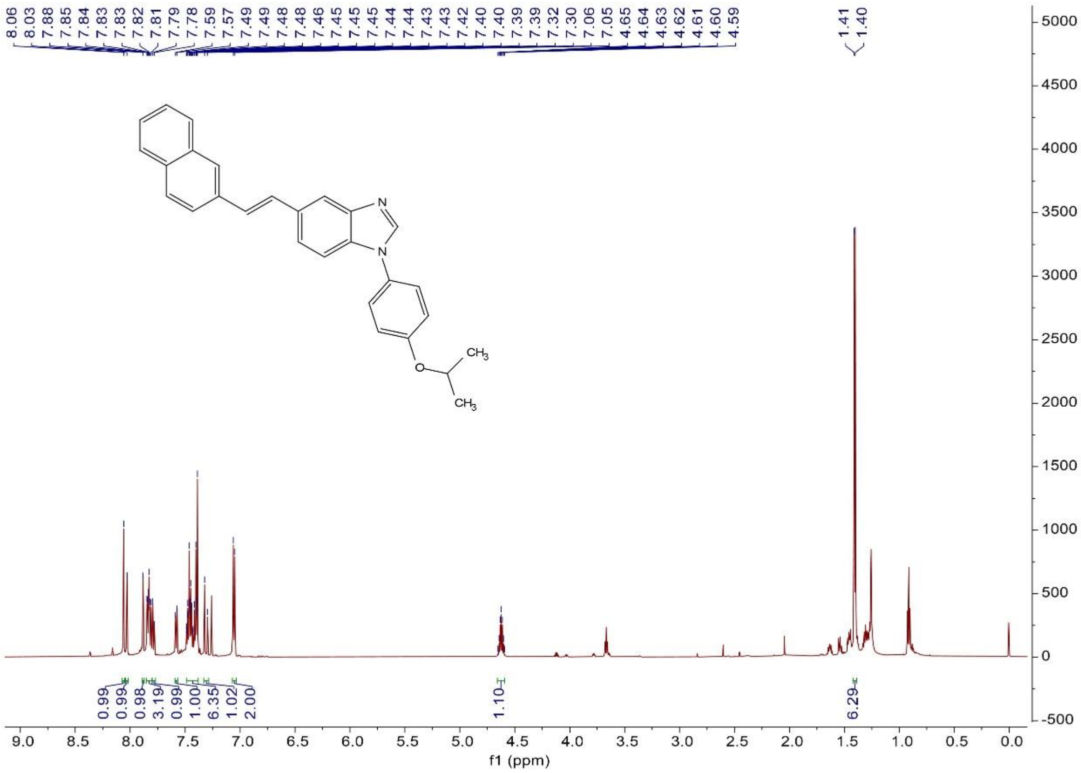

Figure S39. ^{1}H NMR of **7h-E** in $CDCl_3$

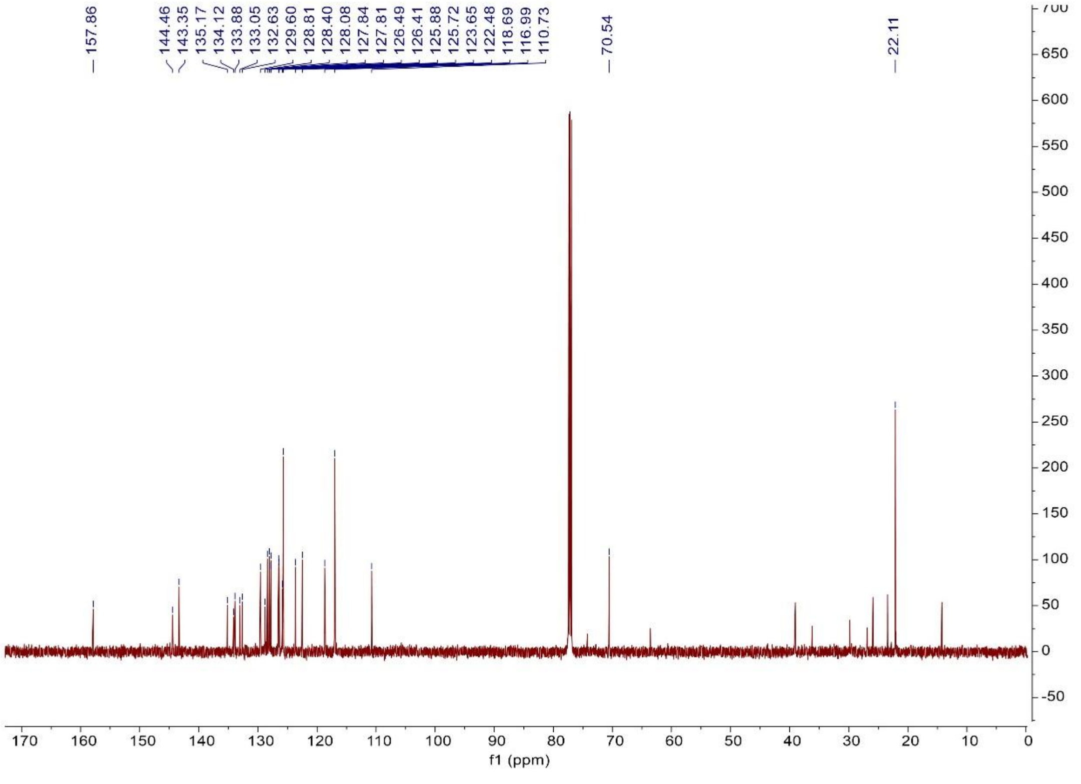

Figure S40. ^{13}C NMR of **7h-E** in $CDCl_3$

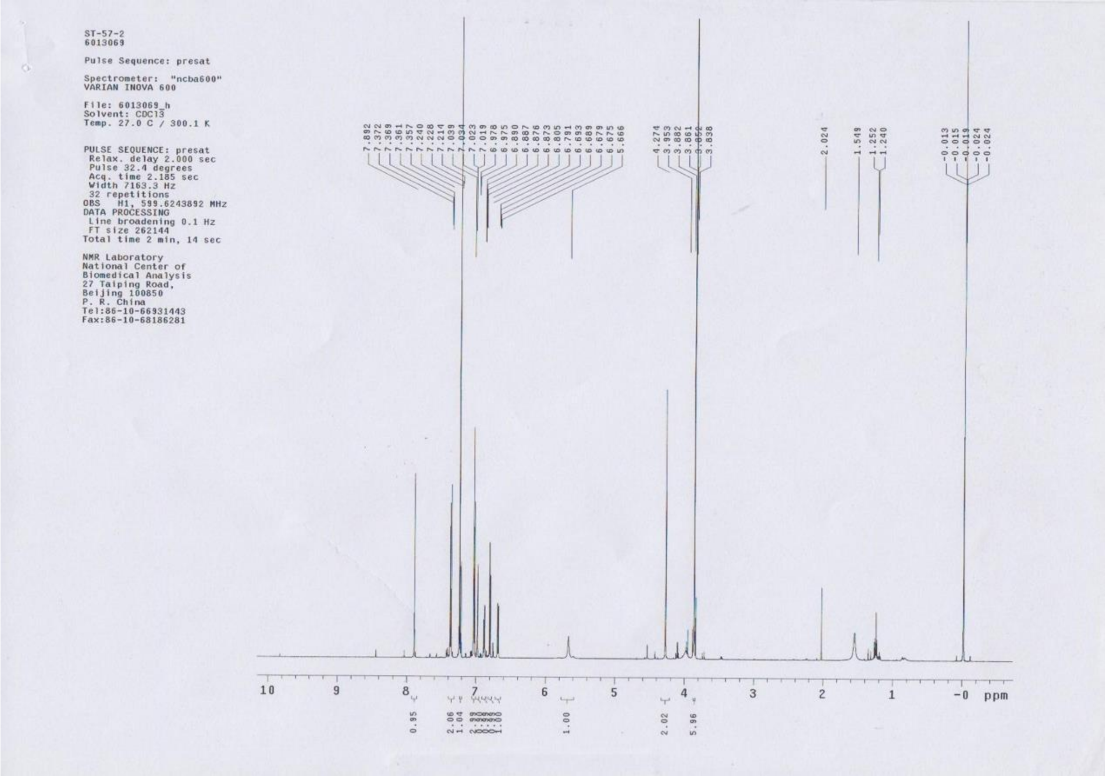

Figure S41. ^1H NMR of **13a** in $CDCl_3$

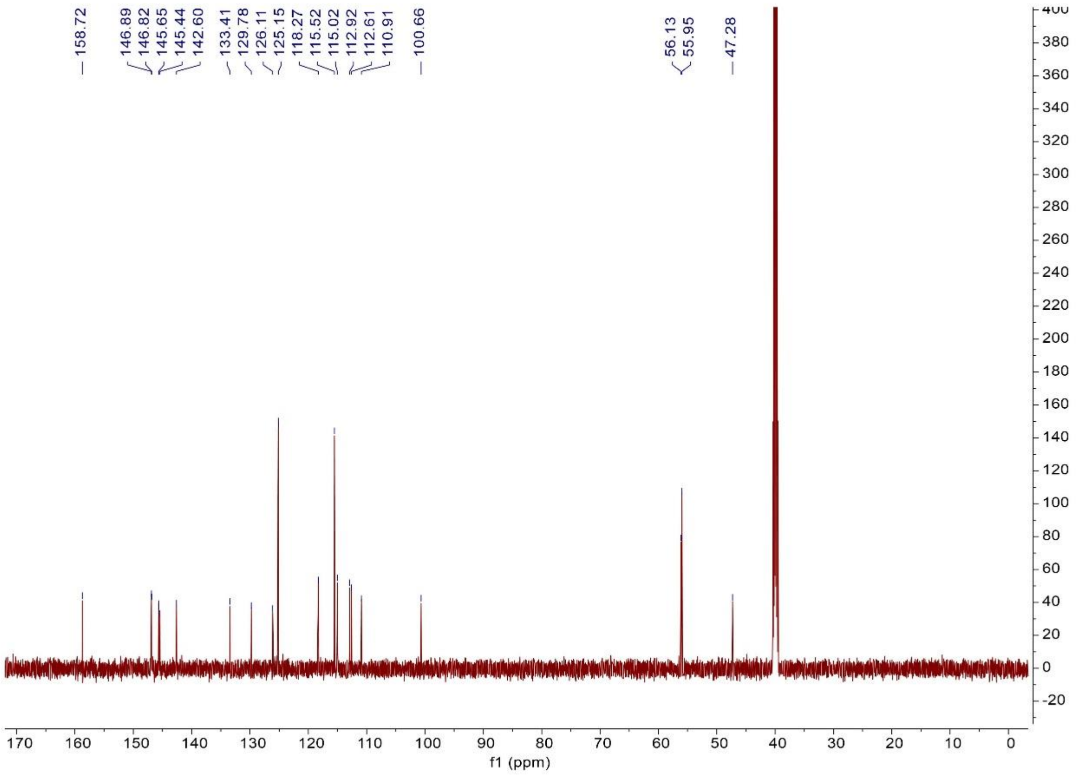

Figure S42. ^{13}C NMR of **13a** in DMSO-*d6*

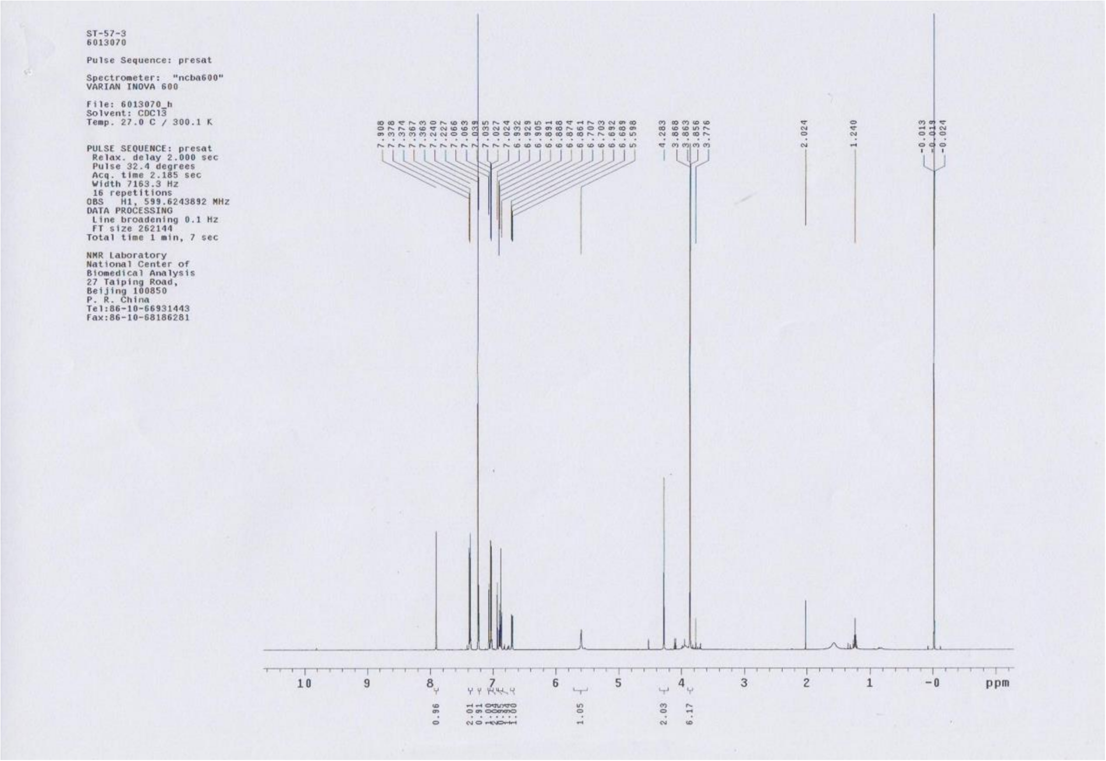

Figure S43. ¹H NMR of **13b** in $CDCl_3$

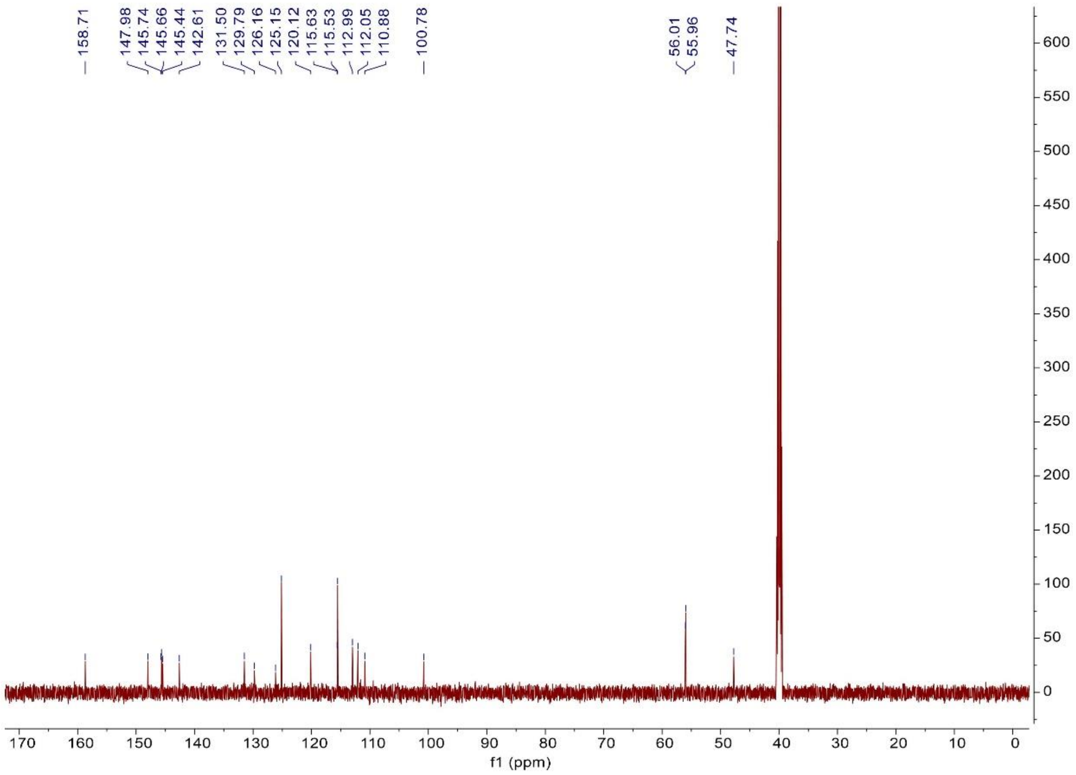

Figure S44. ^{13}C NMR of **13b** in DMSO-*d*6

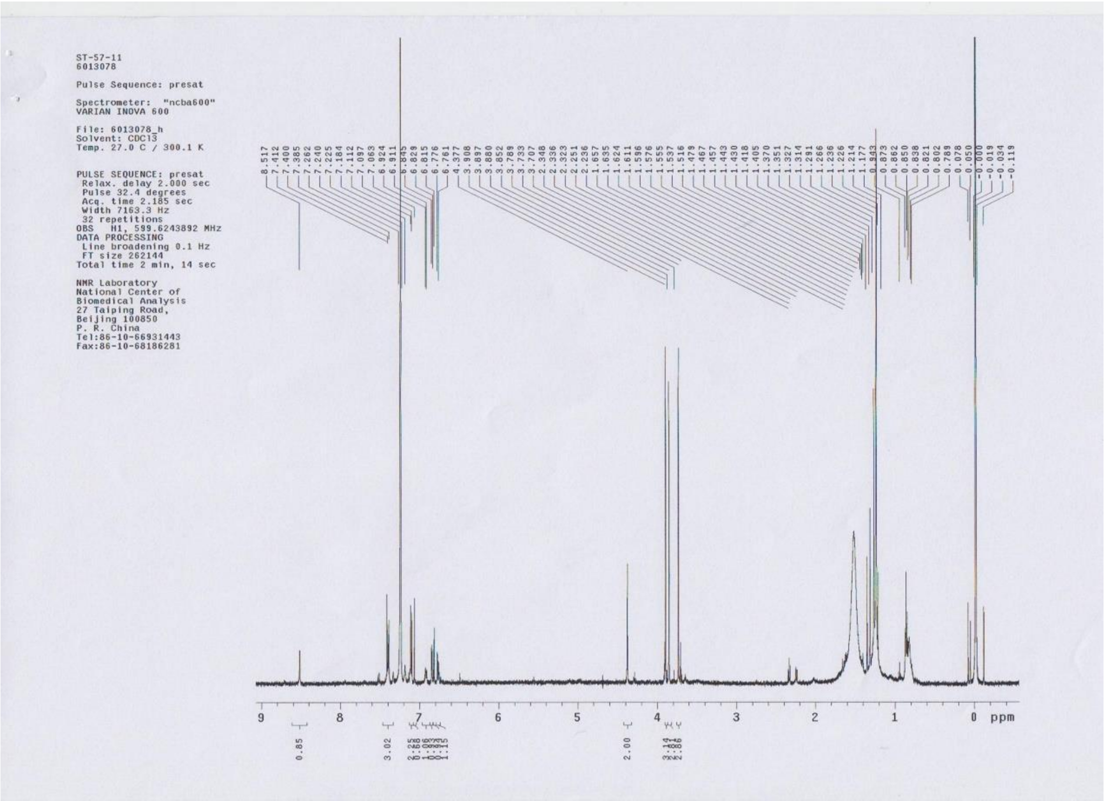

Figure S45. ^{1}H NMR of **13c** in $CDCl_3$

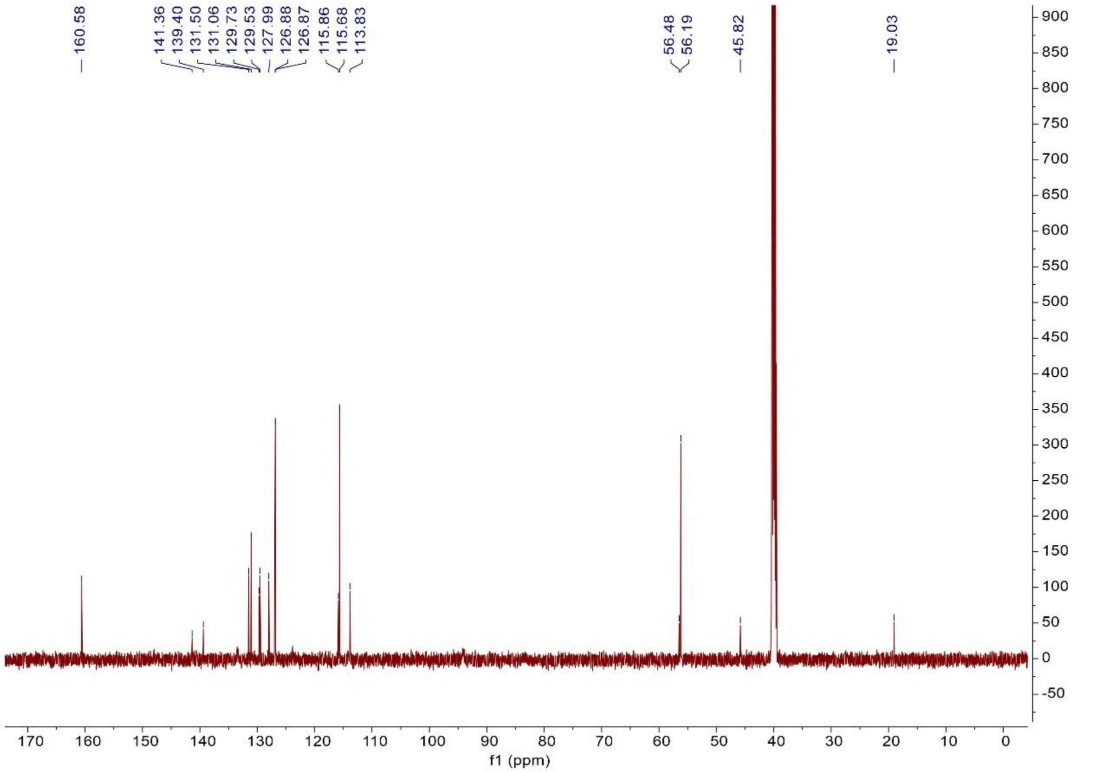

Figure S46. ^{13}C NMR of **13c** in DMSO-*d6*

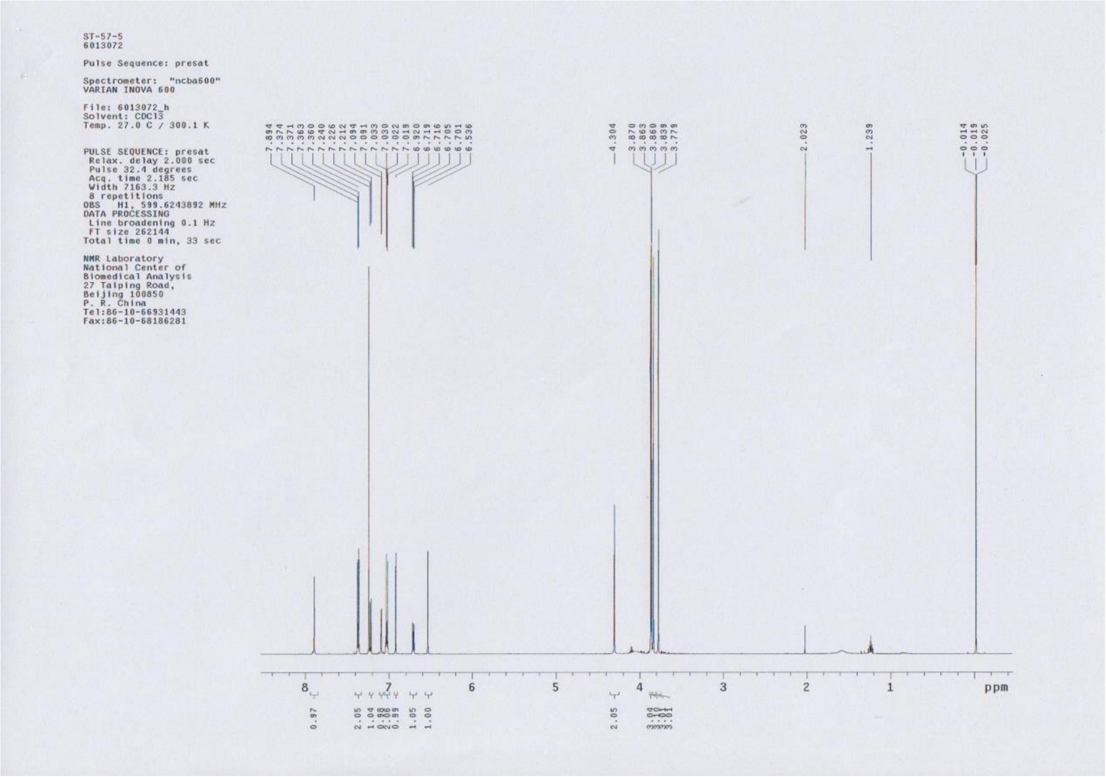

Figure S47. ^{1}H NMR of **13d** in $CDCl_3$

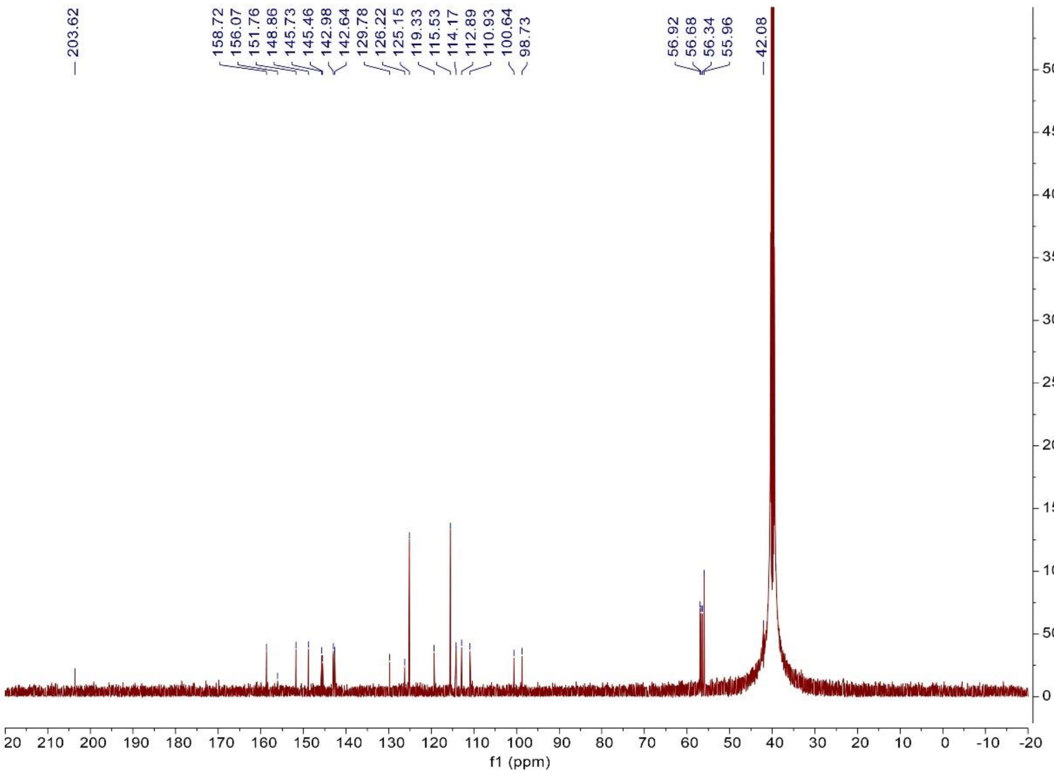

Figure S48. ^{13}C NMR of **13d** in DMSO-*d*6

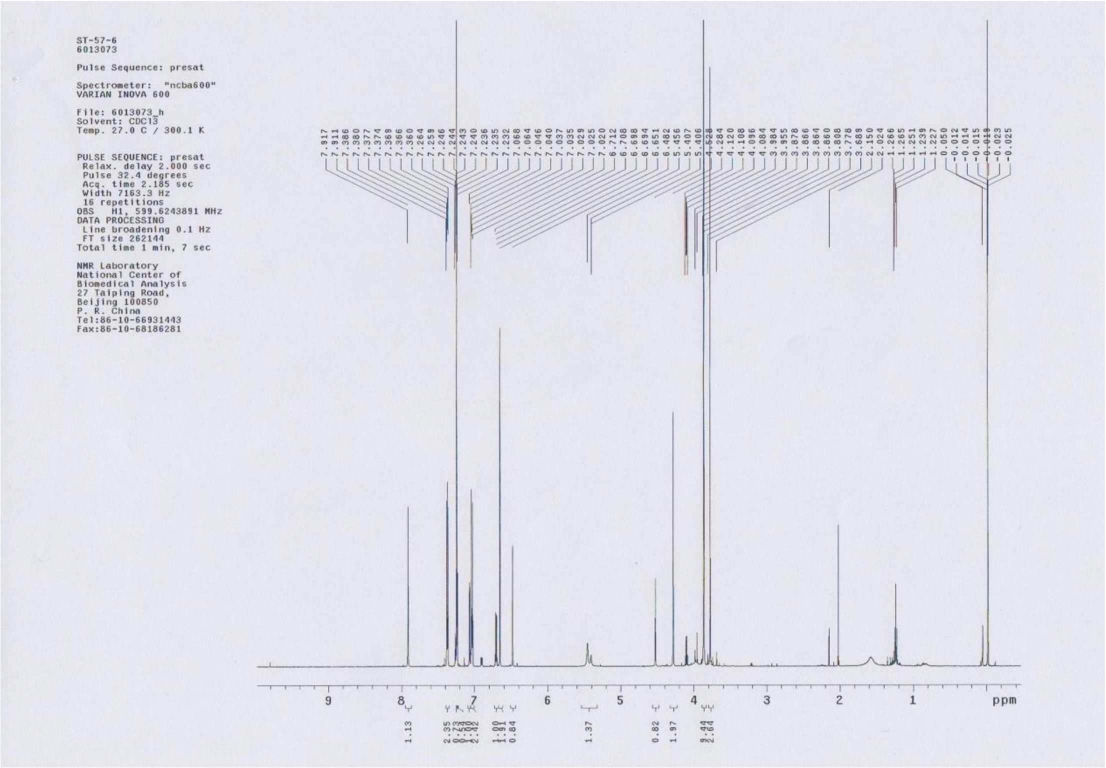

Figure S49. ^{1}H NMR of **13e** in $CDCl_3$

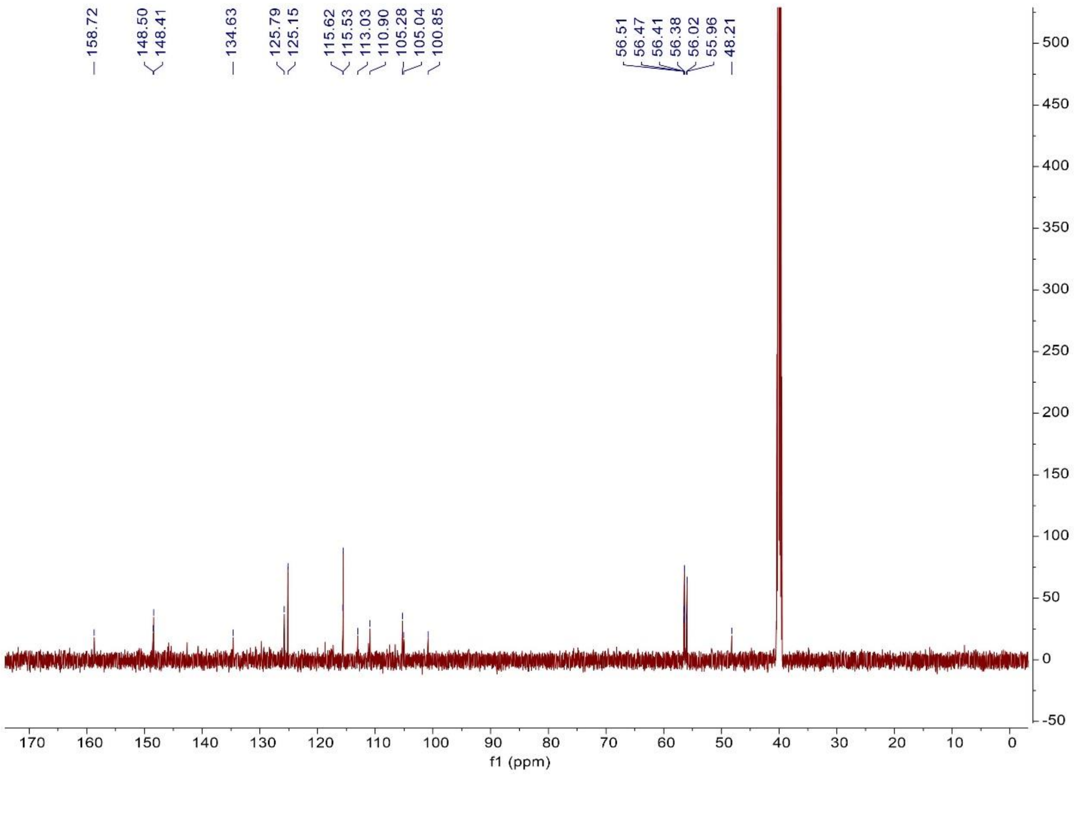

Figure S50. ^{13}C NMR of **13e** in DMSO-*d6*

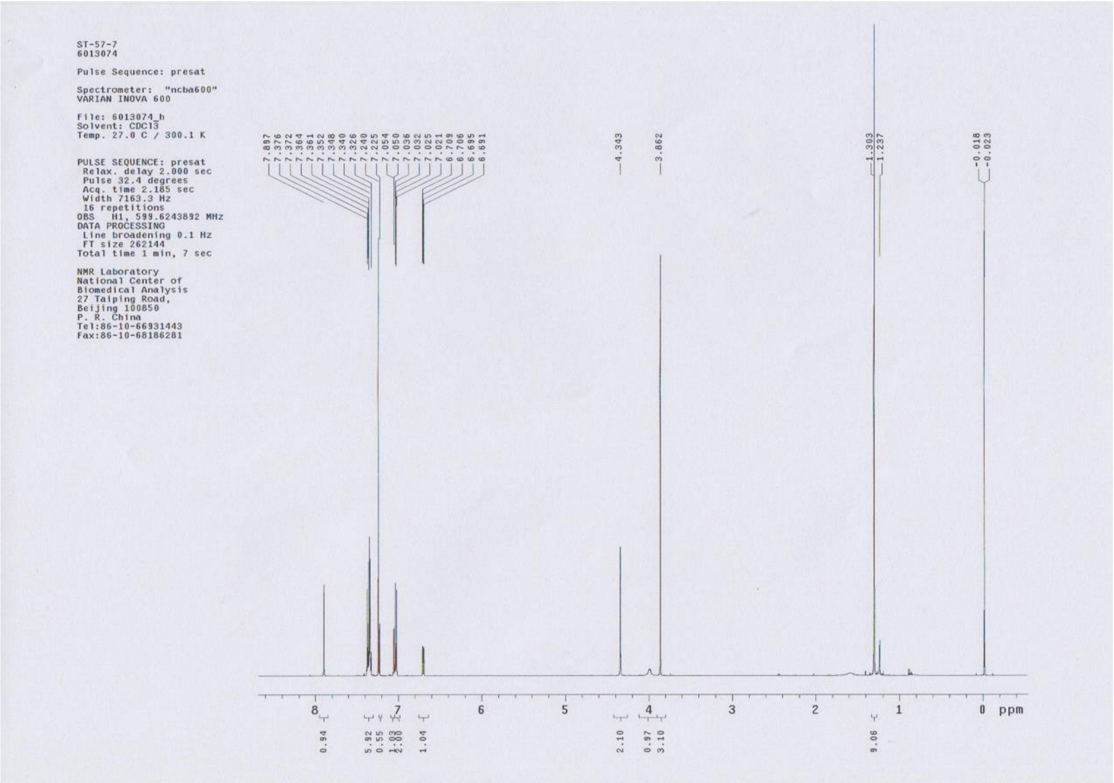

Figure S51. ^{1}H NMR of **13f** in $CDCl_3$

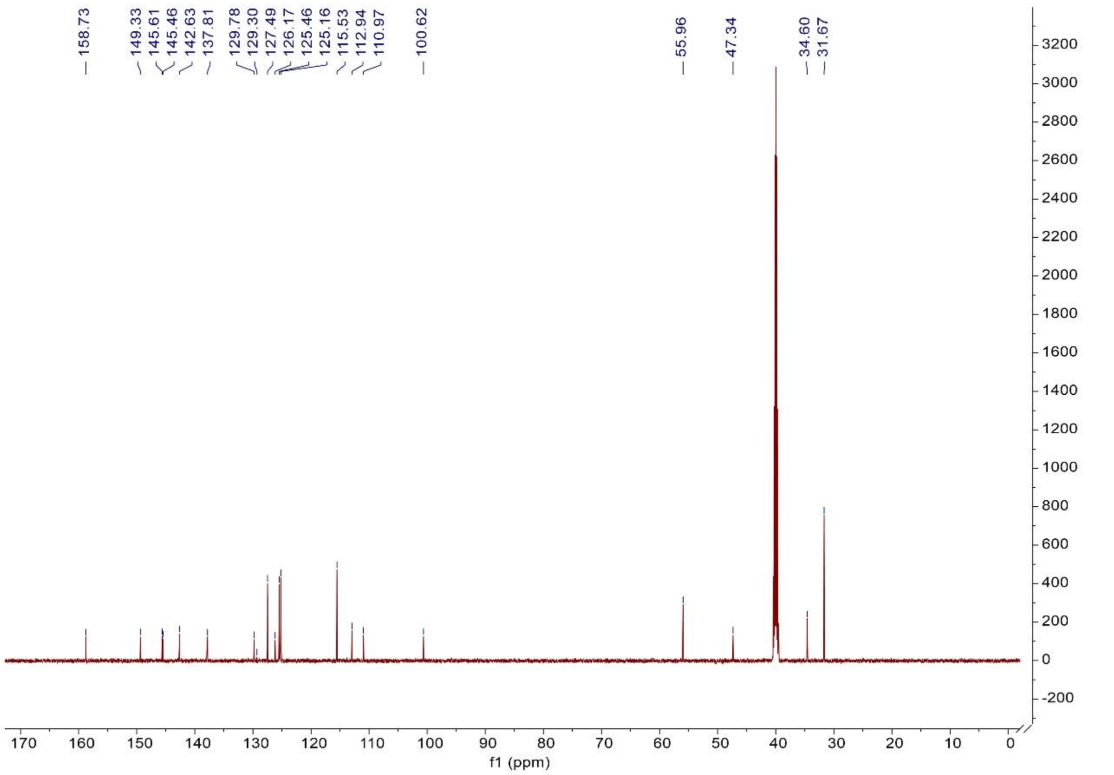

Figure S52. ^{13}C NMR of **13f** in DMSO-*d*6

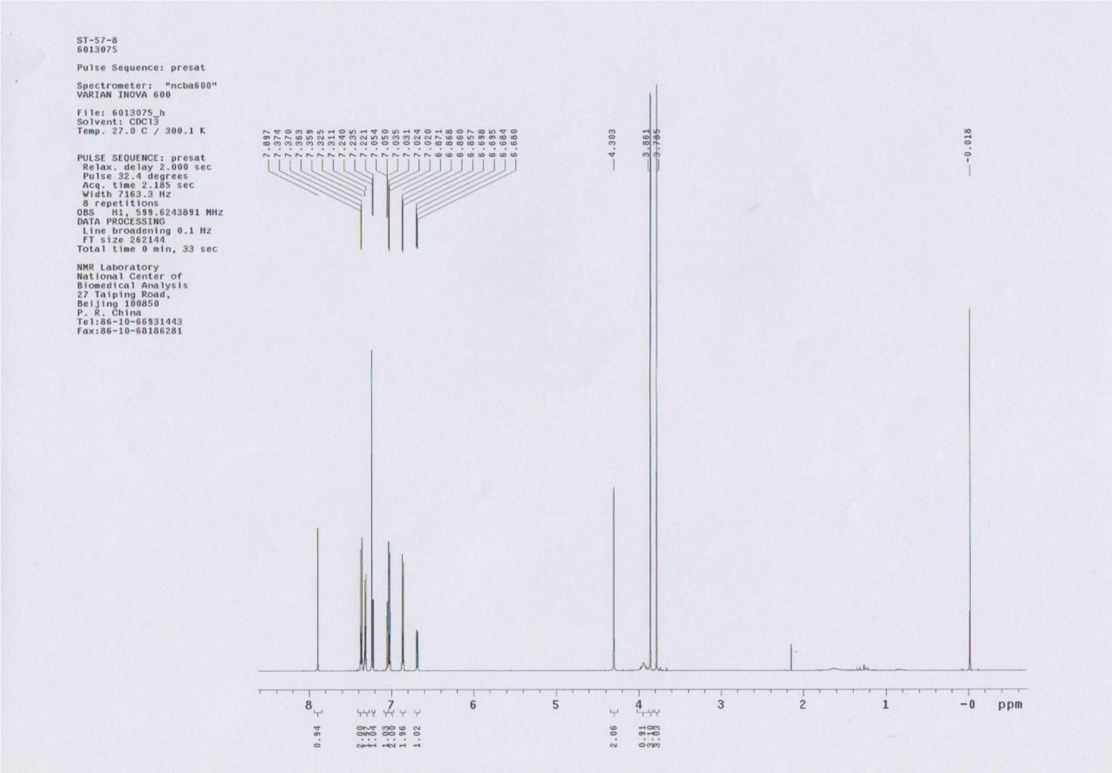

Figure S53. ¹H NMR of **13g** in $CDCl_3$

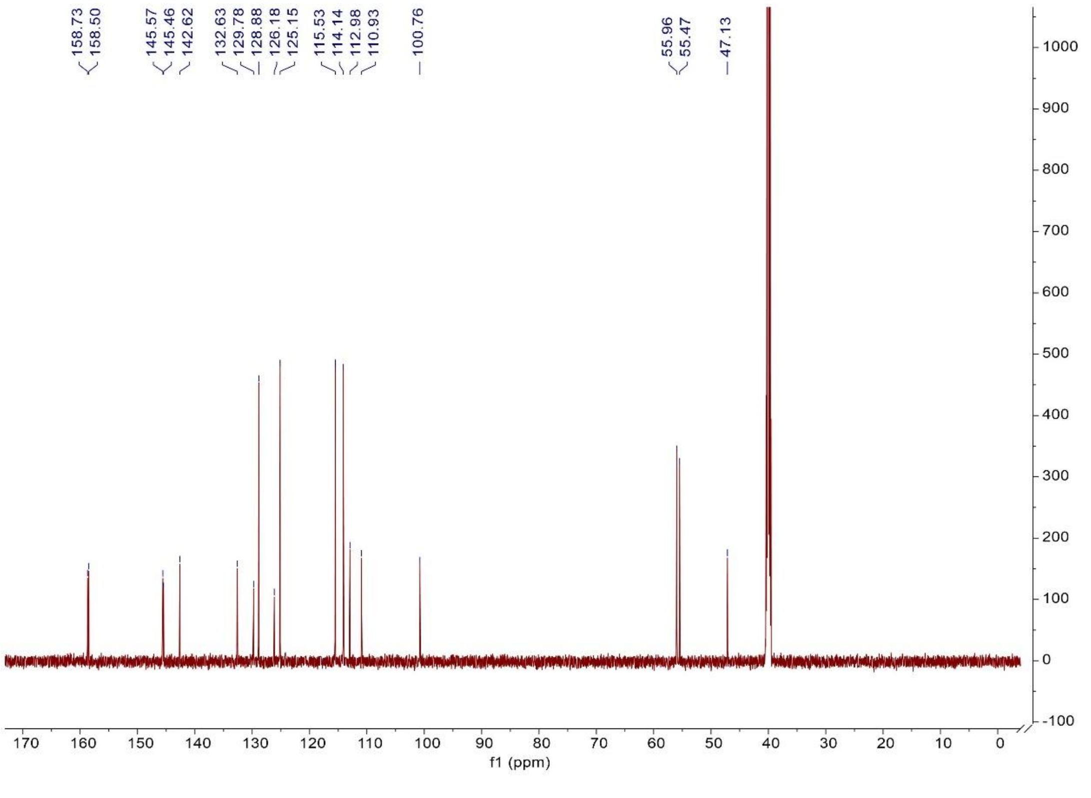

Figure S54. ¹³C NMR of **13g** in DMSO-*d6*

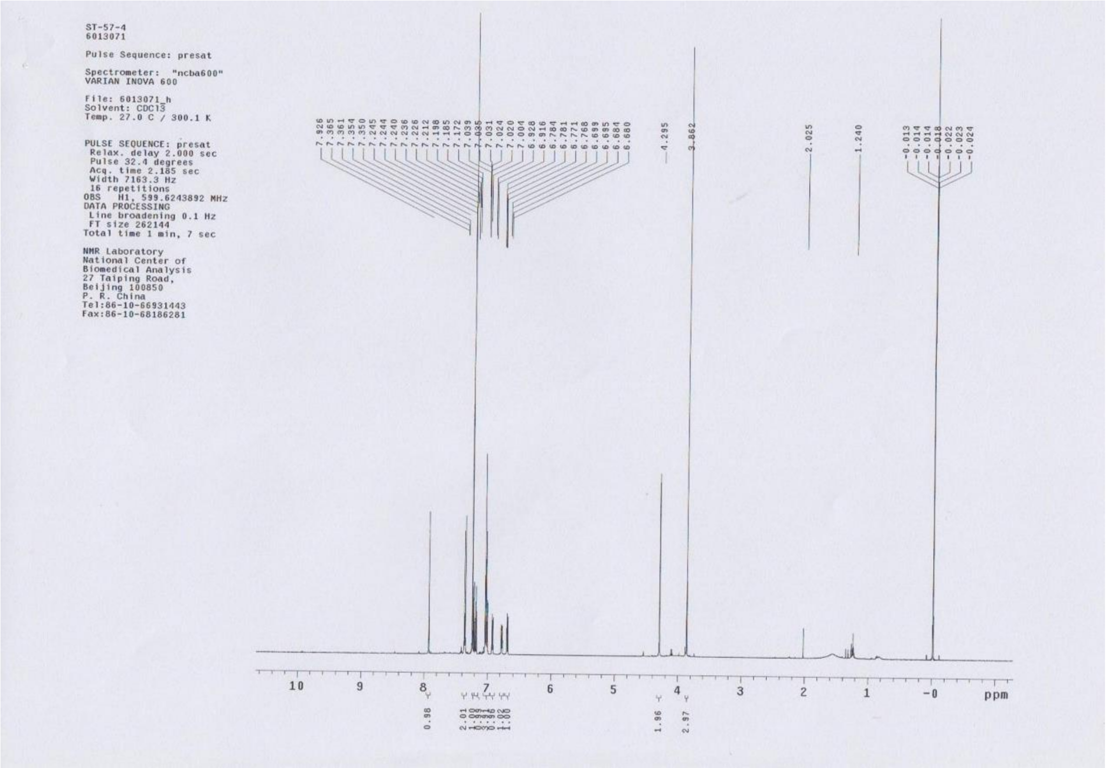

Figure S55. ^{1}H NMR of **13h** in $CDCl_3$

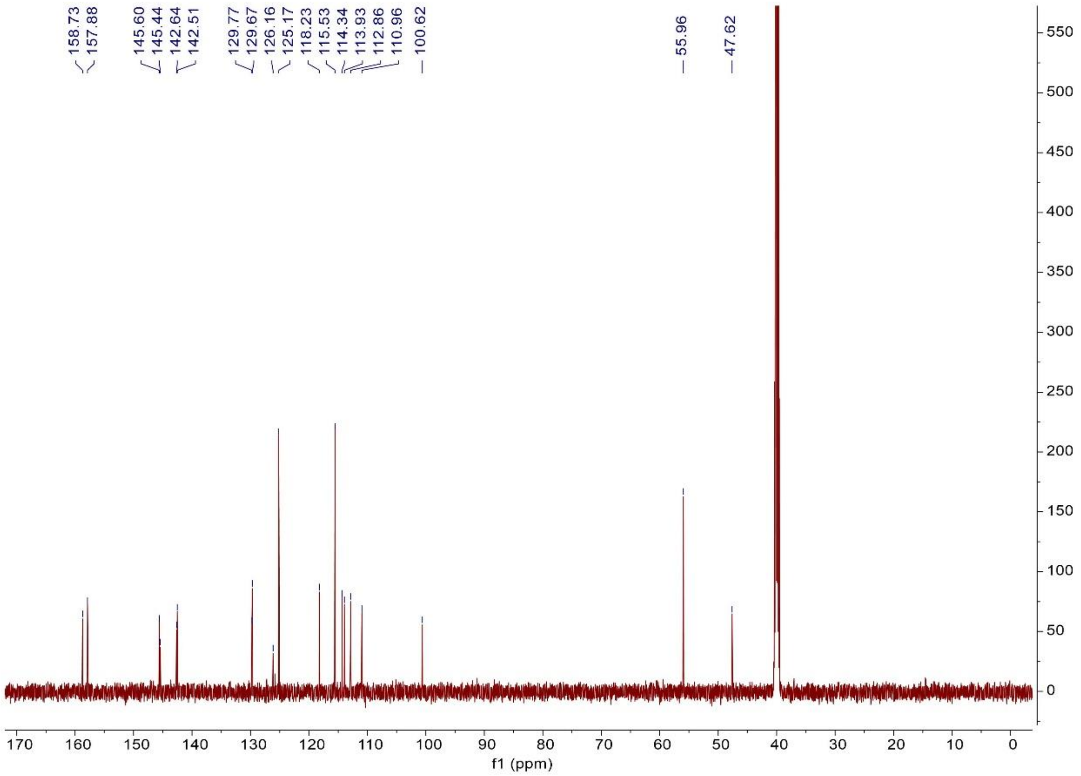

Figure S56. ^{13}C NMR of **13h** in DMSO-*d*6

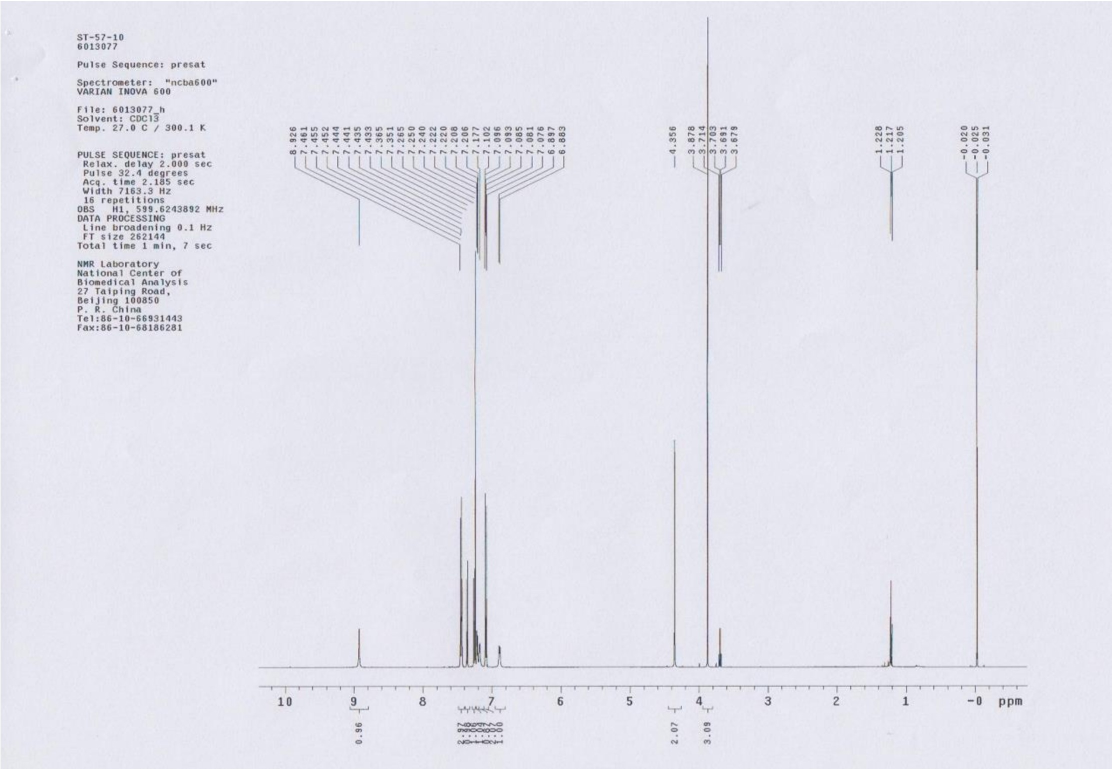

Figure S57. ^{1}H NMR of **13i** in $CDCl_3$

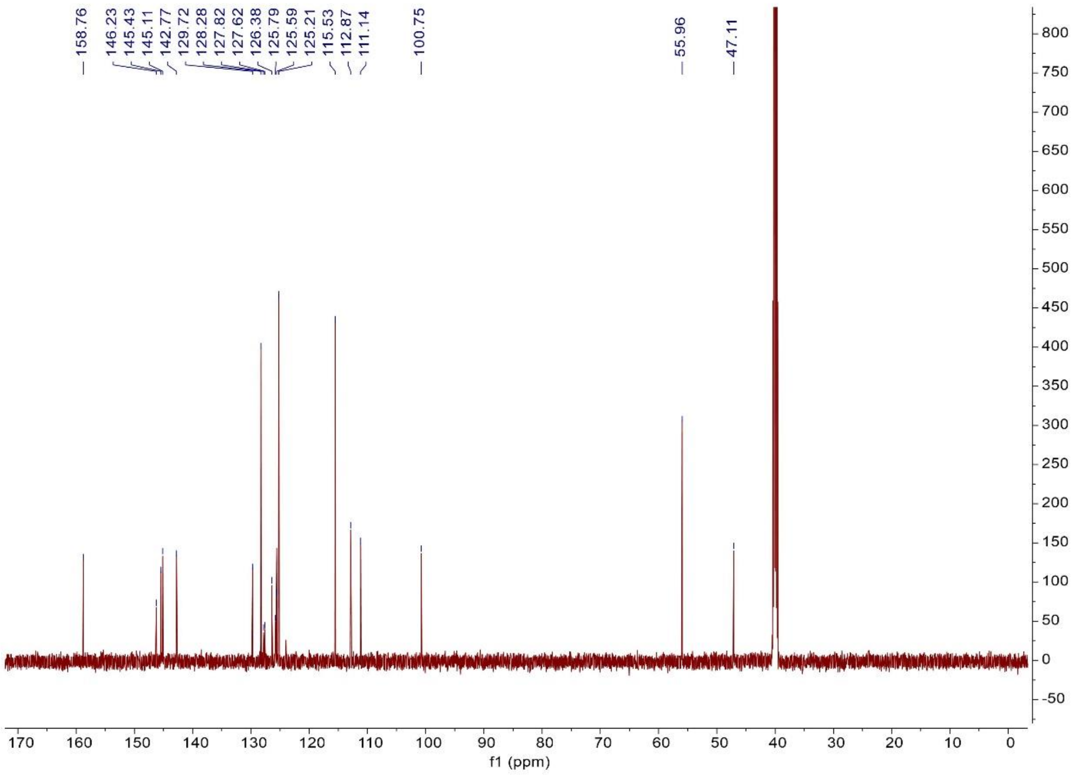

Figure S58. ^{13}C NMR of **13i** in DMSO-*d*6

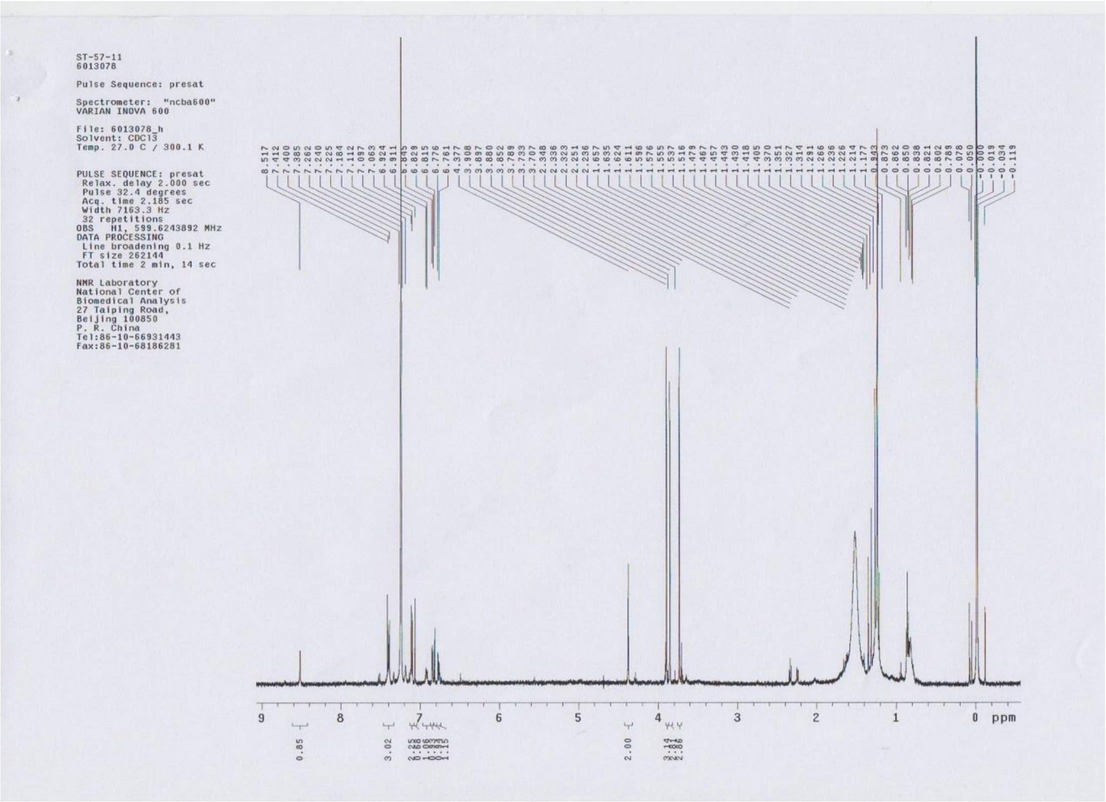

Figure S59. ^{1}H NMR of **13i** in $CDCl_3$

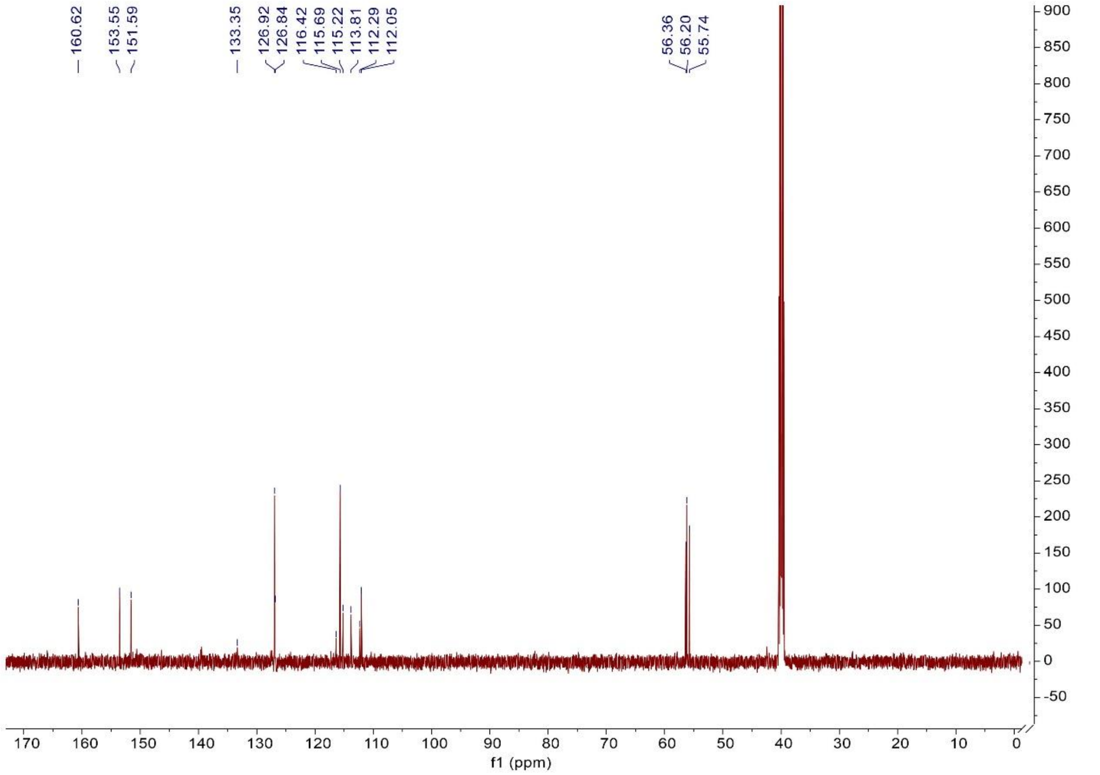

Figure S60. ^{13}C NMR of **13i** in DMSO-*d*6
